# Extracting tumor tissue immune status from expression profiles: correlating renal cancer prognosis with tumor-associated immunome

**DOI:** 10.18632/oncotarget.5052

**Published:** 2015-09-07

**Authors:** Omri Teltsh, Angel Porgador, Eitan Rubin

**Affiliations:** ^1^ The Shraga Segal Department of Microbiology, Immunology, and Genetics, Faculty of Health Sciences, The National Institute for Biotechnology in the Negev, Ben-Gurion University of the Negev, Beer Sheva, Israel

**Keywords:** cancer microenvironment, immunome, precision cancer medicine, RNAseq, TCGA

## Abstract

Investigating the expression of genes in cancer-associated immune cells (immunome) is imperative for prognosis prediction. However, evaluating the expression of immune-associated genes within cancer biopsy is subject to significant inconsistencies related to the sampling methodology. Here, we present *immFocus*, a method for extracting immune signals from total RNA sequencing of tumor biopsies, intended for immunity depiction and prognosis evaluation. It is based on reducing the variation which biopsy preparation adds to the apparent expression levels of immune genes. We employed *immFocus* to normalize gene expression with an immune index using data obtained from renal clear cell carcinoma biopsies. Genes that became less variable due to normalization were found to be preferentially immune-related. Moreover, immune-related genes tended to become more prognostic due to the normalization. These results demonstrate, for the first time, that whole transcriptome sequencing can be used for interrogation of a cancer immunome and for advancing immune-based prognosis.

## INTRODUCTION

Personalized medicine is a novel approach to patient care that is based on fitting a therapy to each patient. In precision cancer medicine, this means comprehensive evaluation of both tumor and patient properties, choosing a therapeutic agent with high likelihood to benefit the patient. Major efforts are underway to find specific markers that predict prognosis, as well as the benefit of a given therapy for an individual patient [1, 2]. This approach has been successful in identifying markers that have been associated with prognosis and the efficacy of several drugs. Most such markers are genetic and expressed by tumor cells [3, 4]; in the case of targeted therapies, patients often respond if their cancer carries a mutation in the targeted gene (e.g. L858R mutations or exon 19 mutations in the gene EGFR and Gefitinib). For other markers, the level of expression is the best indication for or against efficacy (e.g. high levels of HER2 expression and Herceptin).

While tumor genes have been the source of most markers reported so far, a heterogeneous network of stromal, endothelial, innate inflammatory cells and specific immune cells surround or lay within the malignant tumor nests. Intimate interactions between the different cells of the microenvironment and the malignant cells greatly affect tumor development [5]. Therefore, targeting both the malignant cells and their microenvironment is critically important to achieve effective tumor control and to restrain recurrent cancer and micrometastases. Particularly within the cancer microenvironment, the cancer-associated immunome is associated with cancer prognosis [6, 7]. Recently, various targeted immunotherapies have shown efficacy in cancer treatment. Specifically, molecules targeting the PD-1/PD-L1 and the CTLA4/B7 pathways have shown promising results [8]. However, accurately evaluating the expression of immune-associated genes within a cancer biopsy is subject to significant inconsistencies related to the biopsy sampling methodology.

Here, we report a new approach for the normalization of tumor expression profiles that emphasizes expression in the immunome rather than in the tumor cells. We developed *immFocus*, a method for normalizing the expression of immune-associated genes in order to investigate the function of the immune system within tumors. This method is based on the assumption that a large fraction of the variability in apparent expression of genes that are transcribed in the immunome of tumors (rather than the cancer cells) results from the fraction of immune cells that happen to be included in different tumor biopsies due to sample choice and random sampling. We thus propose that by controlling for this artificial variability we can obtain more accurate estimates of immune-related gene expression that in turn can be employed for the prediction of prognosis. To control for total immune cell contents, we calculated a normalization factor by averaging the expression level of a group of immune-correlated genes, whose expression was expected to correlate well with the total number of immune cells in a sample. If our hypothesis is correct, we expect this process to cause immune genes to show less variable and biologically more meaningful expression levels.

## RESULTS

This section is divided into two parts. In the first part, we describe the development of the normalization method, and in the second, we provide evidence that we are indeed detecting immune signals in tumor-derived expression profiles.

### The *immFocus* normalization method

**Figure 1:**
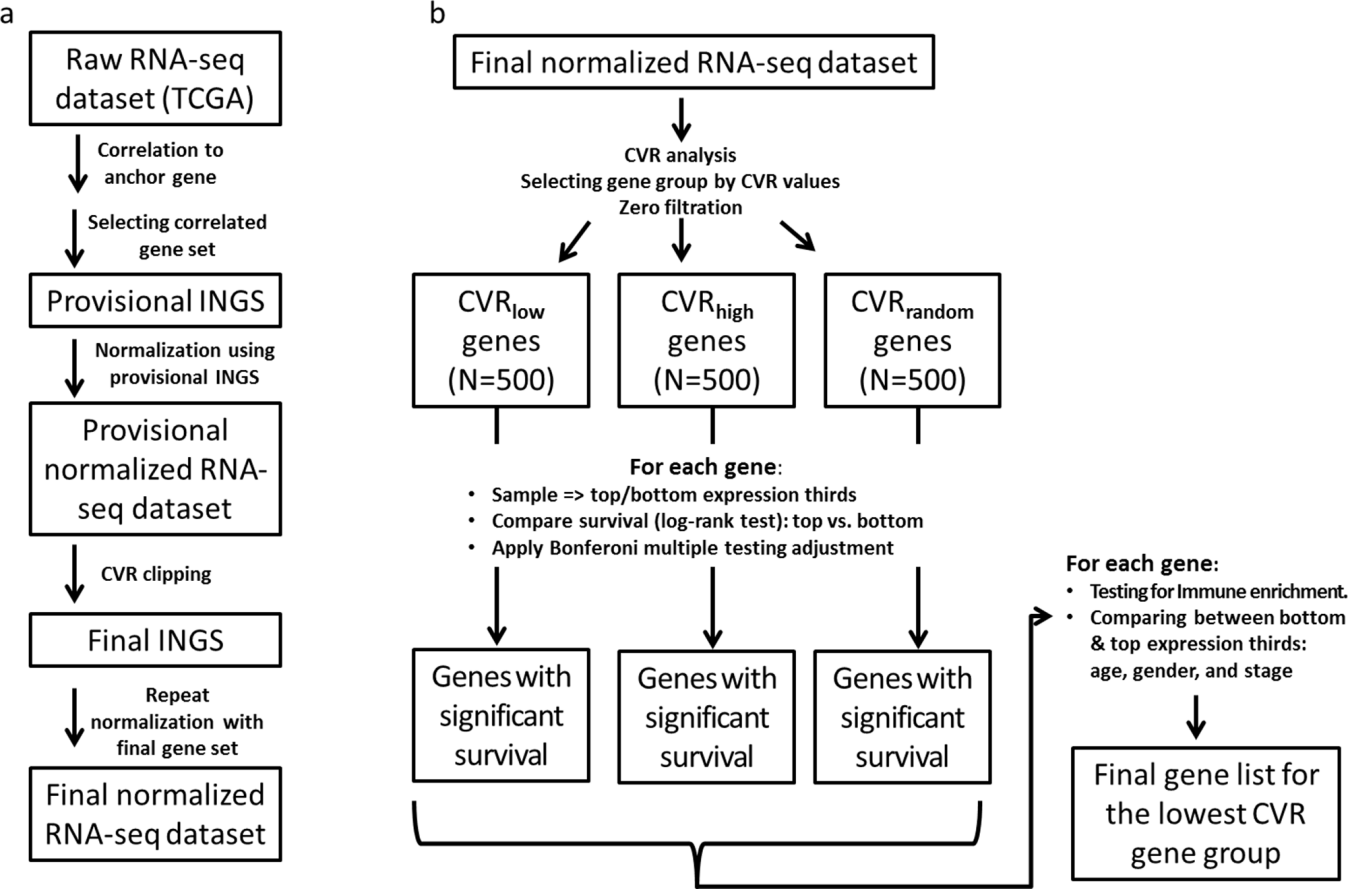
The INGS method for immune normalization from RNA-seq data Boxes represent data, and arrows represent processes. See Methods for a detailed description of each processing step. **a.** Immune normalization process. **b.** Evaluation of the impact INGS normalization has on variation in gene expression between samples and on the prognostic power of gene expression.

Here, we present an immune normalization method (Figure 1a and Methods). Briefly, this method is based on defining an immune-normalizing gene set (INGS) per cancer type, which is defined empirically using an expression measurement found by correlation with the expression of the gene PTPRC. Also known as “Leukocyte Common Antigen”, PRPRC is a tyrosine phosphatase that was shown to play a major role in several immune pathways. PTPRC was chosen since it is currently the best immune cells marker, ubiquitously and almost universally expressed in all types of immune cells and in few other cell types [9]. We note that this approach is likely to be cancer-type specific, as the distribution of immune cell types and their states can also be type specific. The results described are thus specific to the renal clear cell carcinoma (KIRC), which was chosen in *The Cancer Genome Atlas* (TCGA) for having the highest quality data, both in terms of the number of patients and information about survival.

The INGS definition process yielded a final list of 108 genes, of which at least 74 (71%) are annotated as immune-related (see Supplementary Table S1). Manual inspection using GeneCards [10] revealed that many (>95%) of the remaining genes are widely and specifically expressed in immune cells.

Given the INGS, gene expression levels were normalized by using the average expression of the INGS genes (see Figure 1a and Methods).

### *immFocus* normalization preferentially reduces the variation of immune gene expression levels

A good immune normalization factor is expected to reduce the variation in expression levels of immune-related genes by removing some of the noise contributed by immune sampling. In contrast, the variation in the apparent expression of non-immune genes should increase; as the *f*_INGS_ is unrelated to these genes, the normalization should have the effect of dividing gene expression levels by a random factor (which should add noise to the apparent expression measurement). From this observation, we derived a test for the performance of the *immFocus* normalization method: if our proposed approach indeed offers immune normalization, it should preferentially reduce the variation of immune genes and increase or, at the very least, have little impact on the variation of non-immune genes.

**Figure 2:**
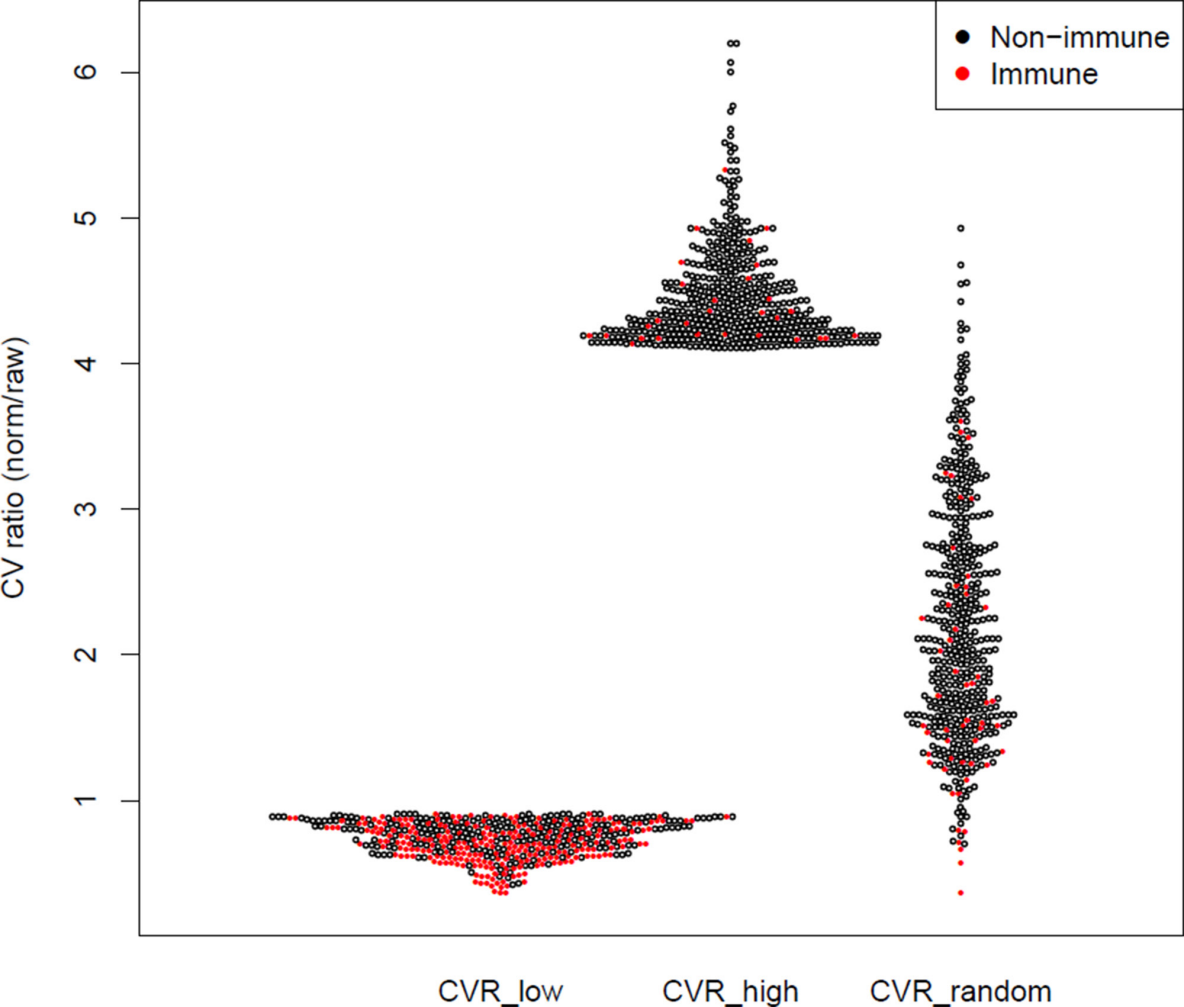
Immune enrichment and CVR values for the lowest, highest, and random CVR gene groups The CV ratio (CVR) of each gene (i.e. the ratio between the CV of normalized and raw measurements) is represented as a dot. Each CVR group contains 500 genes.

To test this prediction, we used the ratio between the coefficient of variation (CVR) before and after normalization to define three groups, each comprising 500 genes (Figure 1b): (1) CVR_low_, representing the genes most responsive to the *immFocus* normalization; (2) CVR_high_, representing the genes with the poorest response to *immFocus* normalization, and (3) CVR_random_, comprising genes randomly chosen regardless of CVR values (see Methods for more detail). As expected from the way these groups were compiled, they differed significantly in their average CVR values, from 0.737 ± 0.122 in the CVR_low_ group to 4.457 ± 0.347 in the CVR_high_ group (*p* < 0.0001, Student's *t*-test) and 2.208 ± 0.829 (*p* < 0.0001, Student's *t*-test) in the CVR_random_ group (Figure 2). The full gene lists are provided in Supplementary Tables S2, S3, and S4.

Comparison of the 3 CVR groups supports the prediction that *immFocus* reflects immune-related expression (Table 1 and Figure 2): at least 257 genes in the CVR_low_ group (51.4%) are annotated as immune related, compared to 29 in the CVR_high_ and 52 in the CVR_random_ sets (*p* < 0.0001, χ^2^ test). Furthermore, manual inspection of the remaining 243 genes from the lowest CVR group revealed that >70% of remaining genes can be clearly associated with immunity. Yet, these genes are not annotated as such in the databases used for immune enrichment examination, thus suggesting that the observed enrichment is an underestimate and the difference between the groups is even larger.

**Table 1:** Low CVR is Associated with Immune Enrichment and Higher Prognostic Power

	CVR_low_			CVR_high_			CVR_random_		
	Immune annotation	Non immune annotation	Total	Immune annotation	Non immune annotation	Total	Immune annotation	Non immune annotation	Total
Entire gene set	257	243	500	29	471	500	52	448	500
Survival* *immFocus*	41	25	66	0	0	0	5	13	18
Survival* raw	16	23	39	5	27	32	10	82	92

The counts represent the number of genes from each CVR group that met the mentioned criteria.

* The counts represent the number of genes whose expression was significantly associated with survival (log-rank test) following multiple testing adjustments (Bonferroni adjustment).

To conclude, genes for which the variation in expression, as reflected in their CV, was reduced by the *immFocus* normalization are more likely to be immune genes than genes for which variation did not decrease.

### Survival analysis

To further demonstrate that the *immFocus* method indeed uncovers true biological signals, we tested the association between gene expression and survival for all the genes in the CVR groups described above (CVR_low_, CVR_high_, and CVR_random_), with and without *immFocus* normalization. For this, a stratification-by-expression approach was used to define two sets of patients and to compare their expression (see Methods).

**Figure 3:**
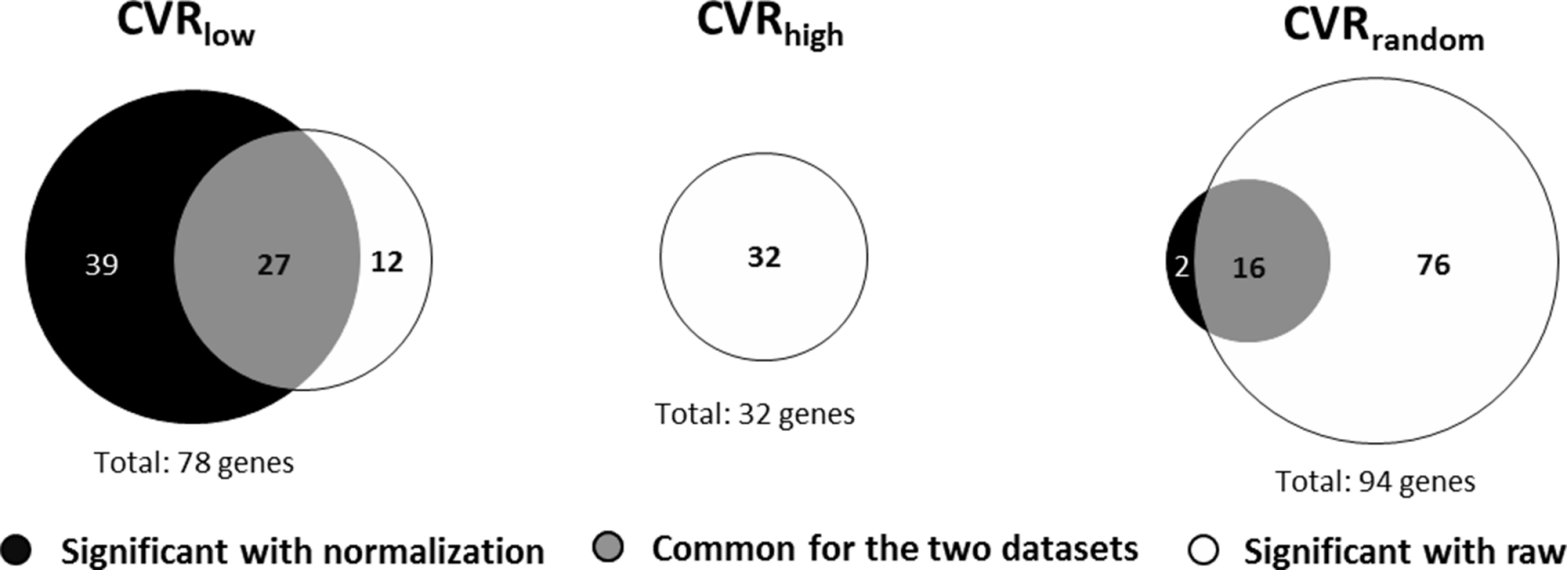
The number of genes predicting survival with and without *immFocus* normalization differs between CVR gene groups The number of genes with statistically significant association with survival is shown for the CVR_low_, CVR_high_, and CVR_random_ gene sets. The association is defined as the significance of the difference in survival between patients in the higher and lower tertile of each gene expression level. The tests for significance were adjusted for multiple testing using the Bonferroni correction.

If indeed *immFocus* normalization teases out immune signals that are relevant to survival, such signals should be strongest in the CVR_low_ group of genes, as this group contains the genes most responsive to the *immFocus* method. Indeed, *immFocus* increased the number of genes significantly associated with survival only for the CVR_low_ group (Table 1 and Figure 3), from 39 to 66. Moreover, only 27 of the genes associated with survival after *immFocus* normalization were also associated with survival without it, while 39 were only significant with the normalization. As also expected, CVR_high_ genes were poorly associated with survival without normalization, and no gene was associated with survival in this set after normalization.

It is interesting to note that the total number of survival-associated genes in the CVR_random_ group was somewhat higher than in the CVR_low_ group: a total of 94 genes were associated with survival in the first group (2 + 16 + 76) compared to 78 in the latter group (39 + 27 + 12). However, 76 (81%) of the CVR_random_ genes were associated with survival only in the absence of *immFocus* normalization, while just 2 (2%) genes were associated with survival only after normalization. In sharp contrast, 12 (15%) of the CVR_low_ genes were associated with survival only in the absence of *immFocus* normalization, while 39 (50%) genes were associated with survival only after normalization.

We further tested our results to see if *immFocus* normalization strengthened the association between gene expression and survival. For this purpose, we employed the log of odds (LOD) ratio of *p*-values for the association with and without normalization. Increased significance after normalization (i.e. lower *p*-value) would result in a negative LOD and decreased significance in a positive LOD. Our results (Table 2) clearly show a tendency toward increased significance of survival association for normalized genes in the low CVR group. A LOD ≤ −2 was obtained for 44 genes (i.e. >7-fold decrease in *p*-value due to *immFocus* normalization), compared to 12 genes with LOD ≥ 2 (i.e. 7-fold less significant association with survival after *immFocus* normalization). In comparison, when the same definitions were applied to the CVR_high_ and CVR_random_ groups, significance was mostly degraded by the *immFocus* normalization (32 degraded vs. 0 improved genes for the CVR_high_ group and 78 vs. 3 for the CVR_random_ group).

**Table 2:** The Effect of Normalization on the Prognostic Power of Expression Levels

Normalization effect on significance of survival difference	CVR_low_			CVR_high_			CVR_random_		
	immune	non immune	total	immune	non immune	total	immune	non immune	total
Improved (LOD ≤ −2)	31	13	44	0	0	0	1	2	3
Degraded (LOD ≥ 2)	2	10	12	5	27	32	5	73	78

The counts represent the number of genes from each CVR group that had markedly different prognostic power in the raw and normalized datasets. For (p-normalized/p-raw), a LOD ≤ −2 represents *a* >7-fold decrease in *p*-value due to *immFocus* normalization, and a LOD ≥ 2 represents >7-fold increase in *p*-value (7-fold less significant) for gene expression predicting survival after *immFocus* normalization. The prognostic power was taken to correspond to the significance of the difference in survival between the upper and lower tertiles (in terms of expression level) of each gene. The log-rank test was used to determine the significance, applying the Bonferroni adjustment for multiple testing. Note that to consider only prognostic genes in this analysis, only genes that showed significant prognostic power in the raw and/or the normalized expression datasets, after Bonferroni adjustment, were considered.

As a final test, we hypothesized that genes for which *immFocus* normalization strengthened the association with survival should be preferentially annotated as immune genes. Our finding supports this prediction: of the 44 genes with improved significance in the CVR_low_ group, 31 are immune annotated, compared to only 2/12 for the decreased significance genes (*p* = 0.001, Fisher exact test). For the two other groups, CVR_random_ and CVR_high_, few if any genes had improved significance, but the degraded significance genes tended to be non-immune (27 vs. 5 and 73 vs. 5, Table 2).

**Figure 4:**
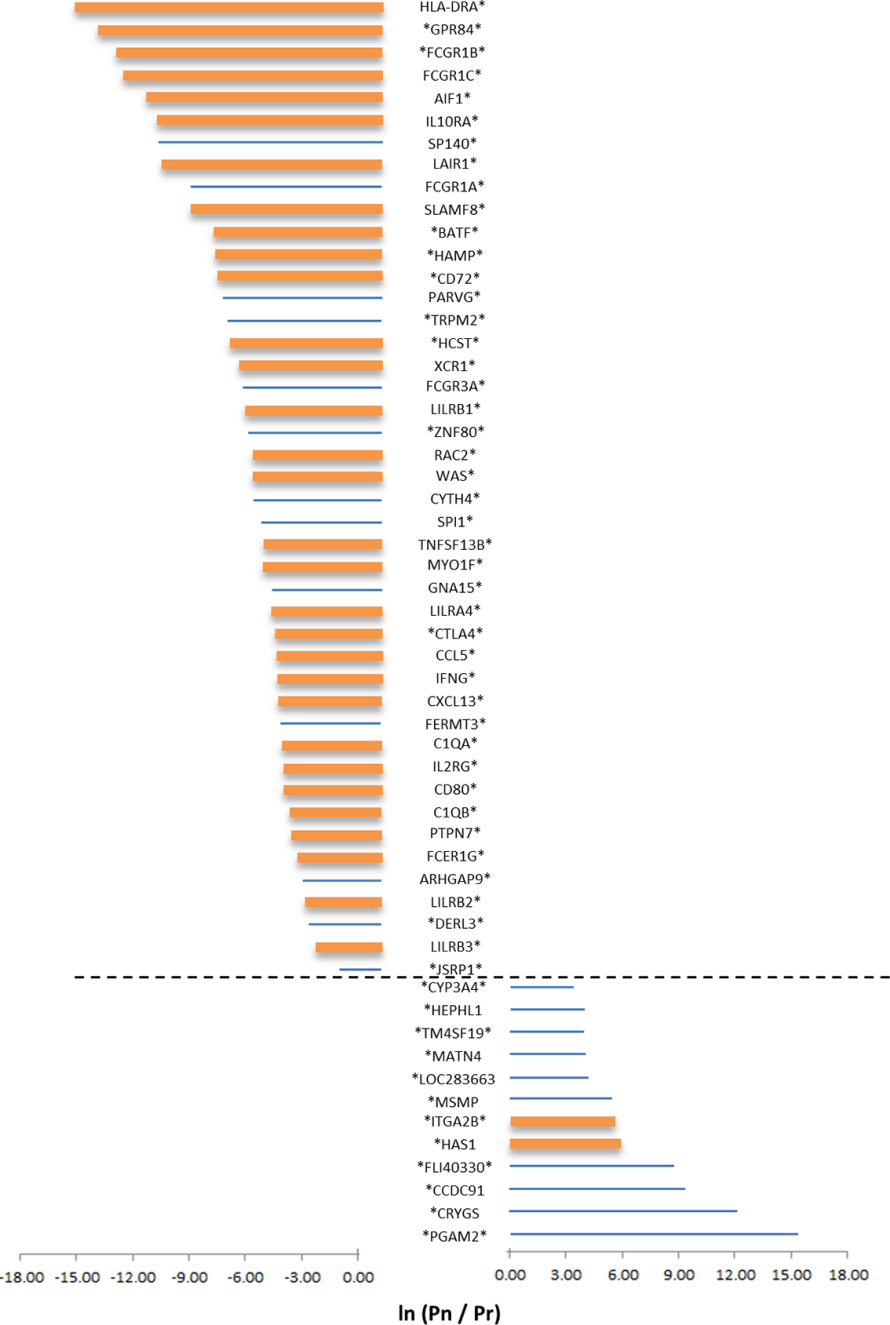
The impact of normalization on the prognostic power of gene expression Selected results are shown for genes from the CVRlow group for which a statistical difference in survival curves between patients with low or high expression value of the genes are observed, either in the normalized or in the raw expression data. Genes were further selected by choosing only genes with a large difference in survival (as reflected in the p-value of the log-rank test for difference in survival curves). Only genes for which the LOD is ≥ 2 or ≤ −2 were selected. Pn = the p-value of the log-rank test with normalized data; Pr = the p-value of the log-rank test for the raw dataset. Significant differences in survival, after multiple testing corrections, using the normalized or non-normalized expression values, is indicated by an asterisk (on the left for normalized data and on the right for raw data). Bar size represents the LOD value; bar color represents immune annotation of the gene (orange = annotated as immune related).

The top panel of Figure 4 details the 44 genes which belong to the CVR_low_ group. These genes were chosen since (i) their normalized expression was significantly associated (after multiple testing adjustment) with survival, and (ii) the survival association was >7-fold more significant following normalization than before (Figure 4, top). It is interesting to note that CTLA4, which encodes an immune-checkpoint inhibitory receptor, is part of this list. However, within this list, CTLA4 is far from being the highest ranking gene (Figure 4); thus, it is tempting to suggest that if CTLA4 is a successful drug target (i.e. blocking its protein products would greatly benefit survival), other genes that rank similarly or higher in their *immFocus* normalization response could also serve as intervention targets, with similar and perhaps even better impact on survival.

The bottom panel of Figure 4 details the 12 genes that belong to the CVR_low_ group, for which (i) raw expression was significantly associated (after multiple testing adjustment) with survival, and (ii) the survival significance was at least 7 times worse following normalization. In both panels of Figure 4, thick bars represent genes that are immune annotated. As aforementioned, immune-annotated genes are abundant in the top panel (31/44) and are rare in the bottom panel (2/12). As before, careful examination of the other 13/44 genes that are non-immune annotated revealed that 12/13 are associated with immunity (Supplementary Table S5).

To further examine the source of association between survival and *immFocus*-normalized expression, we examined the association of other parameters of each biopsy. Specifically, the association between expression and gender, and age and tumor stage was examined. A significant association between expression level and stage (I + II vs III + IV) was found for 30/44 *immFocus* responsive genes described above (Table 3). No significant association was detected between expression and age or gender (Supplementary Table S6).

**Table 3:** Differences in Stage Distribution of Expression-Stratified Populations

#	Direction with survival	Gene name	Lower expression		Higher expression		*p*-value for differences between Stages 1 + 2 vs. 3 + 4 (χ2)
			Stages 1 + 2	Stages 3 + 4	Stages 1 + 2	Stages 3 + 4	
1	+	HLA-DRA	85	87	115	57	0.066
2	−	GPR84	129	43	78	94	***p* < 0.0001**
3	−	FCGR1B	130	42	72	100	***p* < 0.0001**
4	−	FCGR1C	128	44	69	103	***p* < 0.0001**
5	−	AIF1	119	53	88	84	**0.044**
6	−	IL10RA	113	59	87	85	0.2772
7	−	SP140	118	54	75	97	***p* < 0.0001**
8	−	LAIR1	122	50	81	91	***p* < 0.0001**
9	−	FCGR1A	132	40	72	100	***p* < 0.0001**
10	−	SLAMF8	122	50	88	84	**0.0132**
11	−	BATF	126	46	69	103	***p* < 0.0001**
12	−	HAMP	133	39	77	95	***p* < 0.0001**
13	−	CD72	124	48	75	97	***p* < 0.0001**
14	−	PARVG	113	59	82	90	**0.0484**
15	−	TRPM2	114	58	91	81	0.6864
16	−	HCST	124	48	81	91	***p* < 0.0001**
17	+	XCR1	86	86	116	56	0.066
18	−	FCGR3A	116	56	93	79	0.6644
19	−	LILRB1	121	51	86	86	**0.0088**
20	−	ZNF80	111	61	80	92	**0.0484**
21	−	RAC2	113	59	74	98	***p* < 0.0001**
22	−	WAS	117	55	77	95	***p* < 0.0001**
23	−	CYTH4	114	58	84	88	0.0704
24	−	SPI1	120	52	83	89	**0.0044**
25	−	TNFSF13B	126	46	72	100	***p* < 0.0001**
26	−	MYO1F	115	57	88	84	0.1936
27	−	GNA15	109	63	89	83	1
28	+	LILRA4	92	80	115	57	0.6776
29	−	CTLA4	115	57	81	91	**0.0132**
30	−	CCL5	122	50	76	96	***p* < 0.0001**
31	−	IFNG	121	51	76	96	***p* < 0.0001**
32	−	CXCL13	128	44	72	100	***p* < 0.0001**
33	−	FERMT3	112	60	86	86	0.2816
34	−	C1QA	114	58	84	88	0.0704
35	−	IL2RG	125	47	77	95	***p* < 0.0001**
36	−	CD80	108	64	89	83	1
37	−	C1QB	116	56	87	85	0.0924
38	−	PTPN7	125	47	73	99	***p* < 0.0001**
39	−	FCER1G	126	46	80	92	***p* < 0.0001**
40	−	ARHGAP9	120	52	85	87	**0.0088**
41	−	LILRB2	106	66	89	83	1
42	−	DERL3	119	53	79	93	***p* < 0.0001**
43	−	LILRB3	117	55	80	92	**0.0044**
44	−	JSRP1	122	50	78	94	***p* < 0.0001**

The counts represent the distribution of patients who were diagnosed with the mentioned pathological stages. Values in the column “direction with survival”: “−” = more survivors in the low-expression third. “+” = more survivors in the higher-expression third. Significance values in the right column are shown after multiple testing correction and the p-values in bold were significant after correction.

**Figure 5:**
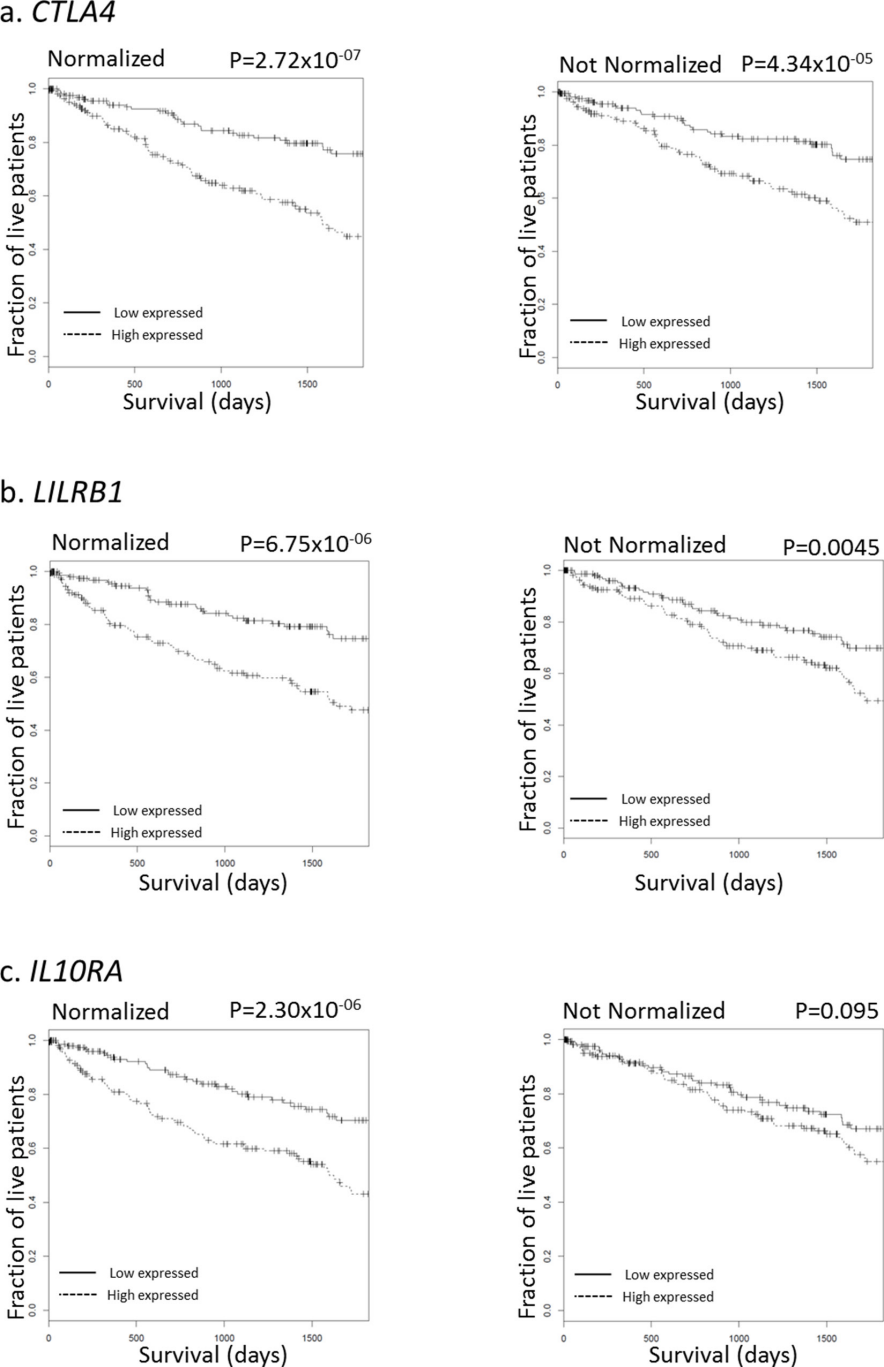
Survival curves for selected genes, with and without *immFocus* normalization For each gene, two subgroups of patients were defined by selecting the patients from the lowest and highest tertiles of that gene's expression level. The survival of the patients from the two groups was compared, both graphically with the Kaplan-Meyer plot and statistically using the log-rank test. Left column: using *immFocus* normalized expression levels. Right column: using raw expression values.

Figure 5 shows normalization-mediated survival prediction improvement for 3 genes (from the 44-gene list) that represent different outcomes. These genes were chosen because (i) a statistically significant difference in survival (with multiple testing adjustment) was found between high-expressing and low-expressing samples in both raw and normalized data, but with a better significance for normalized data (CTLA4 panel, Figure 5a); (ii) the difference in survival was significant in both raw and normalized data, but after adjusting for multiple testing, the difference was only significant for normalized data (LILRB1 panel, Figure 5b); and (iii) the difference in survival was not significant in raw data, but significant in normalized data even after multiple testing adjustment (IL10RA panel, Figure 5c).

## DISCUSSION

Cancer therapy in general, and personalized/precision cancer therapy in particular, should consider the tumor microenvironment and particularly the cancer-associated immunome that is intimately associated with cancer prognosis and its sensitivity to different therapeutic regimens [2, 5, 11–15]. The *immFocus* approach was designed to help shed new light on immune-associated genes. Specifically, we have identified a set of genes whose immune normalized RNA-based expression within the tumor microenvironment is correlated with survival. There appear to be two main principles for elucidating relevant immune-associated genes in the cancer immunome:

- Quantitation of cancer immunome RNA expression in a specific biopsy by employing the immFocus normalization method, which reduces the noise added to the apparent expression level by immune sampling—The *immFocus* method is based on defining a set of immune-associated genes per cancer type with a relatively universal and consistent expression. This set is defined empirically by detected correlated expression with a common immune-related gene known as PTPRC. In the current study, we employed the lowest common denominator to attain a broad spectrum of significant genes within the cancer immunome; by starting with a gene, which is expressed abundantly on most if not all immune cells, we were not focusing on any particular subpopulation of such cells. However, *immFocus* can be extended to study specific subsets of the cancer immunome by generating a normalization set correlated with specific markers of an immune-cell subpopulation. This approach could be employed for cases in which the presence and/or absence of a specific immune cell subtype is reported to be imperative in a cancer immunome associated with survival [16].
- Picking the genes with narrower spread in expression levels following the normalization using expression profiles from clinical samples—For this, we proposed to use the CV ratio (CVR) between the normalized and raw data. In accordance, low CVR indicates the reduction of expression spread for a specific gene, which should be associated with successful normalization. Indeed, the majority of genes that manifested reduced CVR are associated with the immune system.

The results presented in this work clearly suggest that the *immFocus* approach teases biologically significant immune signals from whole-tumor expression profiles. First and foremost, we showed that the majority of the normalization-responsive genes (in terms of CVR) are more likely to be immune associated, as compared to genes that are not responsive. Second, we showed that genes most responsive to normalization are more likely to be associated (positively or negatively) with survival. Finally, we show that those survival-associated genes that were favorably affected by the normalization (in terms of survival association strength) are almost exclusively immune genes.

For the favorably affected genes, we can define 3 different outcomes with regard to prediction of survival and the statistical significance. Considering adjustment for multiple testing, normalization of expression-mediated significance of prediction to 33/44 genes, while only 11/44 were significant without normalization. Overexpression of 41/44 genes is significantly associated with higher virulence of the cancer. The other 3/44 genes, HLA-DRA, XCR1, and LILRA4, manifest the opposite phenotype: overexpression is significantly associated with higher survival. Interestingly, these 3 are also the only genes from the 44-gene list on which overexpression is associated with survival, rather than with cancer virulence. It is worth noting that XCR1 and LILRA4 are expressed by conventional or plasmacytoid DC, and their expression is correlated with induction of potent CTL activity and with activation of an ITAM-mediated signaling pathway [17, 18]. MHC class II expression including HLA-DR is directly associated with positive anti-tumor response [19, 20].

We further explored the other 41/44 genes whose overexpression is associated with cancer virulence. Following Database for Annotation, Visualization, and Integrated Discovery (DAVID) annotation, we identified 21/41 genes that encode for proteins associated with the cell membrane. Immune checkpoints refer to a plethora of inhibitory pathways hardwired into the immune system that are crucial for maintaining self-tolerance and modulating the duration and amplitude of physiological immune responses in peripheral tissues in order to minimize collateral tissue damage [21]. Therefore, these 21 genes could be candidates for immune-checkpoint receptors. CTLA4 and PD-1 are two leading immune-checkpoint receptors with clinically-approved anti-CTLA4 and anti-PD-1 drugs [22–26]. CTLA4 did appear in the final 44-gene list; CTLA4 expression inducing suppression was reported for tumor-draining lymph nodes, but was also reported for cancer microenvironment. *E.g*. higher expression of CTLA4 predicts worse survival in Non-Small-Cell Lung Cancer [27]. PD-1 did not appear in the final 44-gene list: the CVR of PD-1 was 0.75, and its LOD score was well below −2 (−3.27). Yet, its significance of survival prediction for KIRC did not reach the *p*-value threshold following multiple testing adjustment. This result could indicate that in KIRC, PD-1 is less influential as an immune-checkpoint inhibitor and/or enlightens the physiological significance of the other 21 candidates for immune-checkpoint receptors in KIRC. Should we chose to perform a less-stringent adjustment for multiple testing (*e.g*. false discovery rate (FDR) method), PD-1 would be in the final gene list as a gene that its higher expression is associated with cancer virulence. Notably, our method also did not report PDL-1, the ligand for PD-1. In the cancer microenvironment, both cancer cells and antigen-presenting immune cells could express PDL-1. Frequently, genes that are expressed by either tumor cells or immune cells did not pass the first threshold of *immFocus*, which is a significantly reduced CVR. This is consistent with our approach, since normalizing PDL-1 expression according to the level of cancer immunome should not reduce its CV since it is likely to be expressed on tumor cells in some or most samples.

As expected, the bulk of the cell-surface proteins encoded by these 21 genes were already reported to be involved in suppression of immunity; CTLA4 is the dominant representative of this group, but also LAIR1, LILRB1, LILRB2, IL10RA, and others appear in the list. One of the 21 proteins associated with cell membrane was CD80, which is a ligand for both activating CD28 and inhibitory CTLA4 immune receptors. Yet, CTLA4 binds to the B7 family molecules CD80 and CD86 with higher affinity than CD28 [28]. Moreover, expression of CTLA4 and CD80 was directly correlated. Unexpectedly, IFNγ also appears in the 41-gene list whose overexpression is associated with cancer virulence. However, a recently published paper showed that in clear cell renal cell carcinoma, IFNγ expression in T cells purified from the microenvironment is associated with poor prognosis [29]. The *immFocus* approach resulted in the same conclusion for IFNγ, but without the need to specifically test it in T cells purified from the cancer microenvironment. Purifying infiltrating immune subsets and phenotyping them for evaluating cancer immunome of each specific patient can be performed only in advanced clinical institutes, while analyzing RNA expression from a formalin-fixed paraffin-embedded biopsy or a resected tumor can be performed as external central service. Thus, *immFocus* approach could be applied in a broad spectrum of clinical institutes.

Why higher expression of the immunomodulator IFNγ is associated with cancer virulence for this type of kidney cancer is not clear. Cytokine-based immunotherapy with either IFN-α or high-dose interleukin (IL)-2 is a valid treatment option for renal cell carcinoma (RCC). On the basis of its similarity to IFN-α, the immunomodulator IFN-γ was evaluated in several clinical trials for RCCs. A large multicenter phase III trial using IFN-γ as a monotherapy for RCCs was conducted, but this trial found no significant difference between IFN-γ and placebo in overall response rates, time to disease progression, or median survival [30]. Though IFN-γ modulates T cell function, it also modulates MDSC function that suppress immune responses within the cancer microenvironment. RCC is frequently infiltrated with tumor-associated macrophages including MDSC [31].

When we further explored cellular immune distribution of the 44-gene list, we could define representation for most prominent immune cell subsets. Yet, in accordance with the presence of tumor-associated macrophages discussed above, the Gran/Mono cell subset had the highest representation. Novershtern et al. published gene sets that are positively or negatively associated with the development of human hematopoietic immune cell subsets [32]. For hematopoiesis of most immune subsets, the 44-gene list (Figure 4, top panel) included a similar small number of genes that were reported to be involved either of the induction or suppression of subset development [32]. Noticeably, for the genes reported to be involved in the induction of Gran/Mono development [32], 14 appeared in the 44-gene list. Strikingly, for the genes suppressing Gran/Mono development [32], none appeared in the list. An almost universal feature of tumor progression is the activation of abnormal myelopoiesis and the recruitment of immature myeloid cells into tissues [33]. Our results clearly support this known recruitment of newly generated Gran/Mono cells and further indicate that the expression of 14 genes associated with the induction of Gran/mono cell myelopoiesis in the cancer microenvironment are associated with cancer virulence.

The difference in survival based on immune-related expression levels could result directly from the state of the immunome, or could reflect other differences in the tumor that impact both survival and the immunome. While no association between expression and gender or age was found (Supplementary Table S6), a significant association between expression and the distribution of stages (stages 1–2 vs. 3–4) was found for 30 out of the 44 (66%) genes that responded positively to *immFocus* normalization (in terms of survival association) (Table 3). This suggests that the immunome-based difference in survival can be explained, at least in part, by different immune responses at different tumor stages. In fact, this finding could be key to understanding the immune-tumor interactions: these 30 genes might be useful for defining stage-associated differences in the cancer immunome. Moreover, the observed association with survival could be explained, either in part or in full, by stage-specific immune response.

For the remaining 14 genes, no statistically significant association was found between expression and stage. Interestingly, 3/14 genes were the same: HLA-DRA, XCR1, and LILRA4. As discussed above, their overexpression is associated with survival. The overexpression of the remaining 11/14 genes was significantly associated with higher virulence of the cancer. These genes could be considered as better candidates for stage independent “suppressive immunity driver” genes, i.e. immune-associated genes whose overexpression drives stage-independent cancer virulence and thus could be targets for cancer therapeutics. Interestingly, 9/11 were cell-surface proteins and thus could be defined as promising therapeutic targets for the blockade of immune-checkpoint receptors in KIRC [21].

We are not the first to use expression profiles to estimate the immune fraction of a tumor biopsy from expression data. Yoshihara et al describe a method that is based on choosing “known” immune gene sets, and averaging their expression to estimate immune cell content [34]. Our work greatly expands on their work: we utilize a direct method to derive the gene expression signatures from the data rather than rely on generic knowledge of immune cells, as we expect cancers with abnormal immune regulation to present non-canonical patterns. In addition, we continue to utilize an estimated immune cells content to normalize the expression level of all genes, to discover which immune genes are most prominent in the tumors, and to demonstrate their relevance to tumor biology and survival.

It is interesting to note that a rather large number of genes are associated with survival without normalization (Figure 3). This is not surprising; Venet, Dumont and Detours (2011) show that more than 50% of the transcriptome differ between breast cancers tumors that differ in their cell proliferation phenotype. They go on to show that this phenotype is in turn associated with prognosis [35]. The high proportion of genes that are associated with survival in our analysis prior to normalization may thus reflect a similar property of renal cancer tumors, namely an altered expression of many genes in tumors due to some underlying phenotype that also affects prognosis. It is not impossible that the improvement we observe in the prognostic power of selected (mostly immune-related) genes is also the result of some underlying phenotype, although it is reasonable to expect immune response to be more complexly controlled by the tumor. However, even if this improved prognostic power reflects an immune response to a common phenotype, it could be very useful: exploring immune-effecting phenoytpes *in situ* without the *immFocus* approach is extremely difficult.

The current study is exploratory, and involves only a single cohort (from TCGA). To validate the clinical value of the proposed methods it should be validated with independent data sets, or better still through a prospective study. However, correlations with otherwise unrelated features are observed: a clear association was found between normalized expression and immunity and/or survival. It is difficult to contemplate a cohort-specific bias that will result with such associations. As a result we conclude that at least in this cohort of patients, the proposed normalization process helps tease out immune signals from the expression profiles. Obviously, further research is required to assess the generality and clinical applicability of this approach.

To summarize, altogether, our results indicate that *immFocus* preferentially teases out clearer signals for immune-related genes and could be employed for better characterization of differential immune status in cancer stages and for the elucidation of promising targets for cancer therapeutic approaches based on the blockade of immune checkpoints.

## MATERIALS AND METHODS

### TCGA samples

Level 3 RNA-Seq-V2 data (Illumina HiSeq RNA-Seq platform, Illumina, Inc.; San Diego, CA, USA) and corresponding clinical data were downloaded from the TCGA Data Portal (https://tcga-data.nci.nih.gov/tcga/) [36] in August 2014. A total of 480 patients from the renal clear cell carcinoma (KIRC) study were analyzed. Only patients with samples from primary solid tumors were considered. RNA-Seq-V2 results were quantified through RNA-Seq by Expectation-Maximization (RSEM) [37] using the “rsem.gene.normalized_results” file type.

### *immFocus* normalization

The normalization is based on defining an immune-normalizing gene set (INGS), using this set to estimate the fraction of immune cells in the sample and to adjust the measured expression with this fraction.

Defining INGS: A preliminary INGS was defined by using PTPRC as an anchor, choosing all the genes with highly correlated expression (R^2^ > 0.5) to PTPRC. The CVR of each gene in this provisional INGS was then calculated (using the normalization methods described below), and genes with CVR > 0.8 were omitted from the set, yielding a new smaller set which served as the final INGS.

Normalizing gene expression levels: Given the INGS, a biopsy-specific immune normalization factor (*f*_INGS_) was calculated using the averaged expression of the INGS genes. The expression level of each gene *i* was than normalized by dividing the raw expression level by *f*_INGS_. For genes included in the INGS, self-normalization was avoided by calculating a special normalization factor for every gene *i* including *f*_INGS_(*i*), which averaged all the genes in the INGS except for gene *i* as the biopsy-specific immune normalization factor for gene *i*.

### CVR gene group selection

Similarly to Yap et al. 2004 [38], a CVR value was calculated for each gene using the ratio between the coefficient of variation of raw and normalized expression levels. With this statistic, 3 groups of genes were defined (Figure 1b): (1) CVR_low_ - the 500 genes with the lowest CVR values, representing the genes most responsive to the *immFocus*; (2) CVR_high_ - the 500 genes with the highest CVR, representing the genes with the poorest response to *immFocus* normalization, and (3) CVR_random_ - a sample of 500 genes randomly chosen regardless of CVR values.

### Survival analysis

For each studied gene, patients were stratified into two groups: the “high expression” group, containing the top tertile in terms of this gene's expression, including the patients with the highest expression levels of that gene; and the “low expression” group, containing the bottom tertile, i.e. those with the lowest expression of that gene. Survival of patients in the low and high expression groups was compared using the Kaplan–Meier estimator [39] to visualize survival kinetics and the log-rank test for significance of the difference (see the statistics section below for additional details). Significance was estimated using the Bonferroni adjustment for multiple testing. The effect of normalization on significance was calculated as the log-odds (LOD), using a natural base: ${\text{LOD}}_{g}=\text{In}\frac{{p^{\prime }}_{g}}{p_{g}}$ were *g* denotes a gene, *p_g_* denotes the results of the log-rank test for difference in survival using the raw expression levels for gene *g* and *p^′^_g_* denotes the same test results using normalized expression values.

### Immune enrichment

Genes were annotated as immune related if (1) they had been found to be associated with the term “immune response” using the Database for Annotation, Visualization, and Integrated Discovery (DAVID) (http://david.abcc.ncifcrf.gov/) [40] and AmiGO2 (http://amigo.geneontology.org/amigo) to search for assignment, or (2) if they had been found in the immunogenetic-related information source (IRIS) [41].

### Comparing the clinical parameters of “high expression” and “low expression” sets

Differences in clinical parameters between gene-expression subsets (high expression and low expression) were performed similarly to the survival examination (based on upper and lower gene expression), replacing survival time with other variables in the subsequent analyses. We tested the parameters: age, gender, and pathological stage distribution. In order to avoid small numbers, pathological stages 1 & 2 were combined into one group and stages 3 & 4 in another. For details regarding the specific statistical test we used, see the next section.

### Statistical analyses

All the data preprocessing and mentioned analyses except for functional analysis were performed in the R statistical environment (http://www.r-project.org). The Pearson product-moment correlation coefficient was used to measure the correlation between PTPRC and other gene expression. The “raster” package was used to calculate the coefficient of variation (CV). The “beeswarm” package was used to visualize the connection between the CVR and the immune enrichment results. The “survival” package was used to calculate and plot Kaplan–Meier survival curves. Overall survival was examined for significance using the log-rank test. Multiple testing corrections were performed using the Bonferroni adjustment. Thus, the initial significant threshold *p*-value (*p* = 0.05) was divided by the number of examined genes. The difference between the average age of patients from different tertiles was examined using Student's *t*-test, and the differences between the gender and pathological distributions for the same patients were examined using a chi-square test.

## SUPPLEMENTARY TABLES




