# Supplementary material for: Extracting tumor tissue immune status from expression profiles: correlating renal cancer prognosis with tumor-associated immunome
Source: Oncotarget. 2015 Sep 7;6(32):33191–205. doi: 10.18632/oncotarget.5052 (PMC4741758; doi:10.18632/oncotarget.5052)
Supplement: Supplementary file 1 [file oncotarget-06-33191-s001.pdf]

# SUPPLEMENTARY TABLES

## Supplementary Table S1: S_PTCORE gene list

| # | Gene name | CVR | Immune response* | # | Gene name | CVR | Immune response* |
|---|---|---|---|---|---|---|---|
| 1 | NCKAP1L | 0.365 | Y | 55 | ZNF831 | 0.616 | N |
| 2 | CD53 | 0.365 | Y | 56 | SLAMF6 | 0.617 | Y |
| 3 | LCP1 | 0.375 | Y | 57 | SP140 | 0.621 | N |
| 4 | GPR65 | 0.404 | Y | 58 | CD2 | 0.621 | Y |
| 5 | HLA-DPA1 | 0.410 | Y | 59 | SH2D1A | 0.623 | Y |
| 6 | MS4A6A | 0.410 | Y | 60 | CD80 | 0.624 | Y |
| 7 | SNX20 | 0.420 | N | 61 | LOC100233209 | 0.625 | N |
| 8 | IKZF1 | 0.430 | Y | 62 | RASSF5 | 0.625 | N |
| 9 | CD86 | 0.430 | Y | 63 | TRAF3IP3 | 0.626 | N |
| 10 | EVI2B | 0.430 | N | 64 | CLEC7A | 0.629 | Y |
| 11 | HLA-DRA | 0.434 | Y | 65 | RGS18 | 0.630 | N |
| 12 | VAV1 | 0.445 | Y | 66 | SLA2 | 0.634 | Y |
| 13 | SASH3 | 0.457 | Y | 67 | ITK | 0.635 | Y |
| 14 | FCGR3A | 0.464 | N | 68 | PTPN22 | 0.640 | Y |
| 15 | AOAH | 0.473 | N | 69 | MNDA | 0.640 | Y |
| 16 | TLR8 | 0.475 | Y | 70 | SLFN12L | 0.642 | N |
| 17 | PTPRC | 0.480 | Y | 71 | CD244 | 0.645 | Y |
| 18 | CTSS | 0.487 | Y | 72 | ICOS | 0.656 | Y |
| 19 | IL10RA | 0.490 | Y | 73 | CXCR2P1 | 0.661 | N |
| 20 | IL12RB1 | 0.497 | Y | 74 | UBASH3A | 0.661 | N |
| 21 | CCR5 | 0.498 | Y | 75 | CD84 | 0.667 | Y |
| 22 | ARHGAP30 | 0.506 | N | 76 | CD8A | 0.668 | Y |
| 23 | C17orf87 | 0.509 | N | 77 | PYHIN1 | 0.669 | N |
| 24 | NLRC4 | 0.533 | Y | 78 | CD4 | 0.673 | Y |
| 25 | SPN | 0.540 | Y | 79 | TIGIT | 0.673 | N |
| 26 | FYB | 0.549 | Y | 80 | LTA | 0.675 | Y |
| 27 | GPR141 | 0.550 | N | 81 | GZMK | 0.677 | Y |
| 28 | DOCK2 | 0.553 | Y | 82 | WDFY4 | 0.677 | N |
| 29 | SLA | 0.553 | N | 83 | GPR18 | 0.680 | N |
| 30 | BTK | 0.554 | Y | 84 | SAMSN1 | 0.681 | Y |
| 31 | GBP5 | 0.555 | Y | 85 | HCLS1 | 0.689 | N |
| 32 | CD3G | 0.560 | Y | 86 | IL16 | 0.689 | Y |
| 33 | ITGAL | 0.561 | Y | 87 | CD226 | 0.697 | Y |
| 34 | LAPTM5 | 0.562 | N | 88 | SAMHD1 | 0.698 | Y |

(*Continued*)

| # | Gene name | CVR | Immune response* | # | Gene name | CVR | Immune response* |
|---|---|---|---|---|---|---|---|
| 35 | CIITA | 0.566 | Y | 89 | LILRB1 | 0.708 | Y |
| 36 | CYBB | 0.570 | Y | 90 | TNFSF13B | 0.719 | Y |
| 37 | P2RY10 | 0.571 | N | 91 | HLA-DMB | 0.726 | Y |
| 38 | CRTAM | 0.573 | Y | 92 | DTHD1 | 0.727 | N |
| 39 | CD200R1 | 0.575 | Y | 93 | GRAP2 | 0.734 | Y |
| 40 | EOMES | 0.581 | Y | 94 | PLEK | 0.740 | N |
| 41 | MSR1 | 0.582 | Y | 95 | PIK3CG | 0.746 | N |
| 42 | HLA-DOA | 0.587 | Y | 96 | TOX | 0.759 | N |
| 43 | IKZF3 | 0.592 | Y | 97 | ABCD2 | 0.773 | N |
| 44 | TLR7 | 0.595 | Y | 98 | CECR1 | 0.773 | N |
| 45 | TNFSF8 | 0.596 | Y | 99 | IL2RB | 0.776 | Y |
| 46 | LCP2 | 0.599 | Y | 100 | GIMAP2 | 0.781 | N |
| 47 | THEMIS | 0.606 | Y | 101 | TNFRSF9 | 0.797 | Y |
| 48 | GPR174 | 0.607 | N | 102 | CASP1 | 0.834 | Y |
| 49 | CXCR6 | 0.608 | Y | 103 | GVIN1 | 0.879 | N |
| 50 | TRAT1 | 0.608 | Y | 104 | MS4A7 | 0.918 | Y |
| 51 | LCK | 0.609 | Y | 105 | LPXN | 0.955 | Y |
| 52 | RHOH | 0.610 | N | 106 | TLR2 | 0.959 | Y |
| 53 | CD96 | 0.611 | N | 107 | TLR1 | 0.967 | Y |
| 54 | CCR2 | 0.614 | Y | 108 | STAT1 | 1.095 | Y |

*Gray background – genes that are associated with the Immune system.

**Supplementary Table S2: Survival analysis results for Lowest CVR genes group.**

**Supplementary Table S3: Survival analysis results for highest CVR genes group.**

**Supplementary Table S4: Survival analysis results for random CVR genes group.**

## Supplementary Table S5: Possible connection between genes and immunity or cancer

| # | Gene name | Expression in GeneCard | References regarding connection to immunity/cancer | Notes |
|---|---|---|---|---|
| 1 | SP140 | B cells, T cells, NK cells, kidney (basal expression). | http://www.ncbi.nlm.nih.gov/pubmed/19861957 http://www.ncbi.nlm.nih.gov/pubmed/22235315 http://www.ncbi.nlm.nih.gov/pubmed/24635044 | SP140 is a putative tumor suppressor gene previously associated with chronic lymphocytic leukemia/small lymphocytic lymphoma. SP140 is identified as highly specific autoantigen in primary biliary cirrhosis (PBC). SP140 and CD84 were up-regulated in chronic Kawasaki disease cases. |
| 2 | FCGR1A | Myeloid, Monocytes, kidney (basal expression) | http://www.ncbi.nlm.nih.gov/pubmed/23293080 http://www.ncbi.nlm.nih.gov/pubmed/20805361 | The high-affinity human IgG receptor FcγRI (CD64) promotes IgG-mediated inflammation, anaphylaxis, and antitumor immunotherapy. Cytokine-induced immune complex binding to the high-affinity IgG receptor, FcγRI, in the presence of monomeric IgG. |
| 3 | PARVG | T cell Lymphocytes, T cell Leukemia, Myeloid leukimia, Kidney (basal expression) | http://www.ncbi.nlm.nih.gov/pubmed/16517730 | The gamma-parvin-integrin-linked kinase complex is critically involved in leukocyte-substrate interaction. |
| 4 | TRPM2 | Dentritic cells, Kidney (basal expression) | http://www.ncbi.nlm.nih.gov/pubmed/25236871 http://www.ncbi.nlm.nih.gov/pubmed/24756715 http://www.ncbi.nlm.nih.gov/pubmed/24462864 | Transient receptor potential melastatin subfamily member 2 cation channel regulates detrimental immune cell invasion in ischemic stroke. Activation of TRPM2 leads to increases in intracellular Ca(2+) levels, which can serve signaling roles in inflammatory and secretory cells through release of vesicular mediators (e.g., cytokines, neurotransmitters, insulin) and in extreme cases can induce apoptotic and necrotic cell death under oxidative stress. TRPM2 contributes to LPS/IFNγ-induced production of nitric oxide via the p38/JNK pathway in microglia. |
| 5 | FCGR3A (CD16a) | NK cells (very high), Monocytes, Myeloid, Dentritic cells, Kidney (basal expression) | http://www.ncbi.nlm.nih.gov/pubmed/17875730 http://www.ncbi.nlm.nih.gov/pubmed/25550889 | FCGR3A (CD16a) is a promising target for therapy to enhance the cytotoxicity of Immune cells. |
| 6 | ZNF80 | Lymph node, Immune cells and kidney (basal expression). | - | There are no Relevant refrences |
| 7 | CYTH4 | Myeloid, Monocytes, Dentritic cells (high), NK cells, T cells, kidney (basal expression). | http://www.ncbi.nlm.nih.gov/pubmed/23046560 | CYTH4 showed increase in its expression following bacterial infection in milk somatic and white blood cells of goat. |

(*Continued*)

| # | Gene name | Expression in GeneCard | References regarding connection to immunity/cancer | Notes |
|---|---|---|---|---|
| 8 | SPI1 | Myeloid, Monocytes, Dentritic cells, kidney (basal expression). | http://www.ncbi.nlm.nih.gov/pubmed/24504023 http://www.ncbi.nlm.nih.gov/pubmed/23252456 http://www.ncbi.nlm.nih.gov/pubmed/21807633 | Dual regulation of SPI1 transcription factor by heat shock factor 1 (HSF1) during macrophage differentiation of monocytes. Binding sites for the ETS domain transcription factors ELK1, ELK4, and SPI1 were enriched in promoter regions of genes upregulated in the presence of a tumor. |
| 9 | GNA15 | Myeloid, Monocytes, Dentritic cells, kidney (basal expression). | http://www.ncbi.nlm.nih.gov/pubmed/20150327 | GNA15 signaling has been analyzed in the context of hematopoiesis. |
| 10 | FERMT3 | Myeloid, Monocytes, Dentritic cells,NK cells, B lymphoblast, Tcells, kidney (basal expression). | http://www.ncbi.nlm.nih.gov/pubmed/22139635 http://www.ncbi.nlm.nih.gov/pubmed/22431571 | mutations in FERMT3 is suggested as one of the causes for Leukocyte adhesion deficiencies syndrome. |
| 11 | ARHGAP9 | Myeloid, Dentritic cells,NK cells, T cells, kidney (basal expression). | http://www.ncbi.nlm.nih.gov/pubmed/11396949 http://www.ncbi.nlm.nih.gov/pubmed/19911011 | ARHGAP9 is suggested to be involved in regulating adhesion of hematopoietic cells to extracellular matrix. The Ala370Ser polymorphism in the ARHGAP9 gene is associated with coronary artery spasm. These data suggest that the polymorphism of ARHGAP9 has a critical function in the infiltration of hematopoietic cells into the endothelium and inflammation leading to endothelial dysfunction. |
| 12 | DERL3 | RNAseq: white blood cells, Lymph node, kidney (basal expression). | http://www.ncbi.nlm.nih.gov/pubmed/24699711 | the papar sugests DER3 is a key gene in the endoplasmic reticulum-associated protein degradation pathway in human tumours. |
| 13 | JSRP1 | Myeloid, Monocytes, Dentritic cells, NK cells, B lymphoblast, T cells, B cells, kidney. | http://www.ncbi.nlm.nih.gov/pubmed/20442750 | the papar sugests that JSRP1 had a minor assoiation to Graves' disease. |

**Supplementary Table S6: Involvement of gender, age, and pathalogical stage for significant genes in the lowest CVR genes group.**
