# Supplementary material for: Extracting tumor tissue immune status from expression profiles: correlating renal cancer prognosis with tumor-associated immunome
Source: Oncotarget. 2015 Sep 7;6(32):33191–205. doi: 10.18632/oncotarget.5052 (PMC4741758; doi:10.18632/oncotarget.5052)
Supplement: Supplementary file 2 [file oncotarget-06-33191-s002.docx]

| \| **Supplementary Table 2: Survival analysis results for Lowest CVR genes group** \| \| \| \| \| \| \| \| \|  \| \|  \| \| --- \| --- \| --- \| --- \| --- \| --- \| --- \| --- \| --- \| --- \| --- \| --- \| \|  \|  \|  \|  \| **Survival (Logrank test)** \| \| \| \| **log norm** \| \|  \| \| \| \|  \|  \|  \|  \| **Normalized** \| \| **Un-normalized** \| \| **/ raw** \| \|  \| \| \| \| **#** \| **Gene Name** \| **CVR** \| **Survival direction*** \| **Nominal** \| **Bonferroni correction** \| **Nominal** \| **Bonferroni correction** \| **nominal log-rank** \| \| **Immune response**** \| \| \| \| 1 \| GPR84 \| 0.71 \| - \| 1.43E-11 \| 7.17E-09 \| 9.74E-06 \| 4.87E-03 \| -13.43 \| \| Y \| \| \| \| 2 \| FCGR1B \| 0.68 \| - \| 4.30E-11 \| 2.15E-08 \| 1.21E-05 \| 6.03E-03 \| -12.54 \| \| Y \| \| \| \| 3 \| FCGR1C \| 0.71 \| - \| 6.26E-11 \| 3.13E-08 \| 1.28E-05 \| 6.38E-03 \| -12.23 \| \| Y \| \| \| \| 4 \| HAMP \| 0.86 \| - \| 3.61E-09 \| 1.80E-06 \| 9.70E-06 \| 4.85E-03 \| -7.90 \| \| Y \| \| \| \| 5 \| FCGR1A \| 0.63 \| - \| 1.21E-08 \| 6.05E-06 \| 1.05E-04 \| 5.23E-02 \| -9.06 \| \| N \| \| \| \| 6 \| JSRP1 \| 0.89 \| - \| 1.23E-08 \| 6.17E-06 \| 1.02E-07 \| 5.12E-05 \| -2.12 \| \| N \| \| \| \| 7 \| LAIR1 \| 0.60 \| - \| 1.34E-08 \| 6.69E-06 \| 4.51E-04 \| 2.26E-01 \| -10.43 \| \| Y \| \| \| \| 8 \| BATF \| 0.86 \| - \| 2.46E-08 \| 1.23E-05 \| 6.98E-05 \| 3.49E-02 \| -7.95 \| \| Y \| \| \| \| 9 \| ZNF80 \| 0.78 \| - \| 2.50E-08 \| 1.25E-05 \| 1.49E-05 \| 7.44E-03 \| -6.39 \| \| N \| \| \| \| 10 \| CD72 \| 0.66 \| - \| 2.56E-08 \| 1.28E-05 \| 6.29E-05 \| 3.15E-02 \| -7.81 \| \| Y \| \| \| \| 11 \| SP140 \| 0.62 \| - \| 3.65E-08 \| 1.83E-05 \| 1.47E-03 \| 7.36E-01 \| -10.60 \| \| N \| \| \| \| 12 \| TRPM2 \| 0.79 \| - \| 4.21E-08 \| 2.11E-05 \| 6.56E-05 \| 3.28E-02 \| -7.35 \| \| N \| \| \| \| 13 \| HCST \| 0.84 \| - \| 5.02E-08 \| 2.51E-05 \| 6.57E-05 \| 3.28E-02 \| -7.18 \| \| Y \| \| \| \| 14 \| HLA-DRA \| 0.43 \| + \| 1.96E-07 \| 9.78E-05 \| 3.82E-01 \| 1 \| -14.48 \| \| Y \| \| \| \| 15 \| CTLA4 \| 0.81 \| - \| 2.72E-07 \| 1.36E-04 \| 4.34E-05 \| 2.17E-02 \| -5.07 \| \| Y \| \| \| \| 16 \| AIF1 \| 0.73 \| - \| 3.17E-07 \| 1.58E-04 \| 2.17E-02 \| 1 \| -11.14 \| \| Y \| \| \| \| 17 \| XCR1 \| 0.65 \| + \| 4.14E-07 \| 2.07E-04 \| 3.54E-04 \| 1.77E-01 \| -6.75 \| \| Y \| \| \| \| 18 \| FOXP3 \| 0.83 \| - \| 4.41E-07 \| 2.20E-04 \| 6.43E-08 \| 3.22E-05 \| 1.92 \| \| Y \| \| \| \| 19 \| DERL3 \| 0.82 \| - \| 5.89E-07 \| 2.95E-04 \| 1.96E-05 \| 9.80E-03 \| -3.50 \| \| N \| \| \| \| 20 \| EREG \| 0.85 \| - \| 1.20E-06 \| 6.02E-04 \| 3.78E-07 \| 1.89E-04 \| 1.16 \| \| Y \| \| \| \| 21 \| CXCL13 \| 0.85 \| - \| 1.22E-06 \| 6.11E-04 \| 1.69E-04 \| 8.46E-02 \| -4.93 \| \| Y \| \| \| \| 22 \| PARVG \| 0.80 \| - \| 1.38E-06 \| 6.92E-04 \| 2.75E-03 \| 1 \| -7.60 \| \| N \| \| \| \| 23 \| SLAMF8 \| 0.89 \| - \| 1.52E-06 \| 7.59E-04 \| 1.28E-02 \| 1 \| -9.04 \| \| Y \| \| \| \| 24 \| DEF6 \| 0.86 \| - \| 1.88E-06 \| 9.39E-04 \| 3.75E-06 \| 1.88E-03 \| -0.69 \| \| Y \| \| \| \| 25 \| IL10RA \| 0.49 \| - \| 2.30E-06 \| 1.15E-03 \| 9.49E-02 \| 1 \| -10.63 \| \| Y \| \| \| \| 26 \| PROM2 \| 0.80 \| - \| 2.55E-06 \| 1.28E-03 \| 5.48E-07 \| 2.74E-04 \| 1.54 \| \| N \| \| \| \| 27 \| LILRA4 \| 0.85 \| + \| 3.00E-06 \| 1.50E-03 \| 5.76E-04 \| 2.88E-01 \| -5.26 \| \| Y \| \| \| \| 28 \| C12orf69 \| 0.86 \| - \| 3.17E-06 \| 1.59E-03 \| 1.15E-05 \| 5.76E-03 \| -1.29 \| \| N \| \| \| \| 29 \| WAS \| 0.67 \| - \| 3.22E-06 \| 1.61E-03 \| 1.46E-03 \| 7.32E-01 \| -6.12 \| \| Y \| \| \| \| 30 \| SPI1 \| 0.71 \| - \| 3.55E-06 \| 1.77E-03 \| 1.16E-03 \| 5.80E-01 \| -5.79 \| \| N \| \| \| \| 31 \| C1QA \| 0.74 \| - \| 4.11E-06 \| 2.05E-03 \| 4.99E-04 \| 2.50E-01 \| -4.80 \| \| Y \| \| \| \| 32 \| CYTH4 \| 0.68 \| - \| 4.28E-06 \| 2.14E-03 \| 1.95E-03 \| 9.73E-01 \| -6.12 \| \| N \| \| \| \| 33 \| ARHGAP9 \| 0.80 \| - \| 4.73E-06 \| 2.36E-03 \| 2.11E-04 \| 1.05E-01 \| -3.80 \| \| N \| \| \| \| 34 \| LILRB1 \| 0.71 \| - \| 6.75E-06 \| 3.38E-03 \| 4.48E-03 \| 1 \| -6.50 \| \| Y \| \| \| \| 35 \| PTX3 \| 0.72 \| - \| 7.19E-06 \| 3.60E-03 \| 1.67E-05 \| 8.34E-03 \| -0.84 \| \| Y \| \| \| \| 36 \| TNFSF13B \| 0.72 \| - \| 8.69E-06 \| 4.35E-03 \| 2.41E-03 \| 1 \| -5.63 \| \| Y \| \| \| \| 37 \| PTPN7 \| 0.70 \| - \| 9.47E-06 \| 4.74E-03 \| 7.57E-04 \| 3.78E-01 \| -4.38 \| \| Y \| \| \| \| 38 \| FCER1G \| 0.62 \| - \| 1.23E-05 \| 6.16E-03 \| 7.18E-04 \| 3.59E-01 \| -4.07 \| \| Y \| \| \| \| 39 \| C1QB \| 0.49 \| - \| 1.39E-05 \| 6.93E-03 \| 1.16E-03 \| 5.81E-01 \| -4.43 \| \| Y \| \| \| \| 40 \| GNA15 \| 0.80 \| - \| 1.58E-05 \| 7.90E-03 \| 3.06E-03 \| 1 \| -5.26 \| \| N \| \| \| \| 41 \| HSH2D \| 0.82 \| - \| 1.60E-05 \| 8.00E-03 \| 1.87E-05 \| 9.33E-03 \| -0.15 \| \| N \| \| \| \| 42 \| FMNL1 \| 0.80 \| - \| 1.93E-05 \| 9.63E-03 \| 4.20E-05 \| 2.10E-02 \| -0.78 \| \| N \| \| \| \| 43 \| MYO1F \| 0.78 \| - \| 2.02E-05 \| 1.01E-02 \| 5.59E-03 \| 1 \| -5.62 \| \| Y \| \| \| \| 44 \| IL2RG \| 0.64 \| - \| 2.09E-05 \| 1.05E-02 \| 2.32E-03 \| 1 \| -4.71 \| \| Y \| \| \| \| 45 \| TM4SF19 \| 0.88 \| - \| 2.18E-05 \| 1.09E-02 \| 5.94E-07 \| 2.97E-04 \| 3.60 \| \| N \| \| \| \| 46 \| PSTPIP1 \| 0.89 \| - \| 2.29E-05 \| 1.15E-02 \| 1.14E-04 \| 5.70E-02 \| -1.60 \| \| Y \| \| \| \| 47 \| RAC2 \| 0.71 \| - \| 2.38E-05 \| 1.19E-02 \| 1.12E-02 \| 1 \| -6.16 \| \| Y \| \| \| \| 48 \| IFNG \| 0.74 \| - \| 2.46E-05 \| 1.23E-02 \| 3.47E-03 \| 1 \| -4.95 \| \| Y \| \| \| \| 49 \| FLJ40330 \| 0.72 \| - \| 2.57E-05 \| 1.28E-02 \| 1.09E-08 \| 5.44E-06 \| 7.77 \| \| N \| \| \| \| 50 \| VGF \| 0.82 \| - \| 2.72E-05 \| 1.36E-02 \| 1.33E-05 \| 6.64E-03 \| 0.72 \| \| N \| \| \| \| 51 \| CYP3A4 \| 0.63 \| + \| 2.85E-05 \| 1.43E-02 \| 1.35E-06 \| 6.75E-04 \| 3.05 \| \| N \| \| \| \| 52 \| LILRB3 \| 0.81 \| - \| 3.08E-05 \| 1.54E-02 \| 6.90E-04 \| 3.45E-01 \| -3.11 \| \| Y \| \| \| \| 53 \| LIPA \| 0.90 \| + \| 3.13E-05 \| 1.56E-02 \| 5.39E-05 \| 2.69E-02 \| -0.54 \| \| N \| \| \| \| 54 \| LAG3 \| 0.73 \| - \| 3.23E-05 \| 1.61E-02 \| 9.64E-05 \| 4.82E-02 \| -1.09 \| \| Y \| \| \| \| 55 \| LILRB2 \| 0.69 \| - \| 3.36E-05 \| 1.68E-02 \| 1.35E-03 \| 6.77E-01 \| -3.70 \| \| Y \| \| \| \| 56 \| CD80 \| 0.62 \| - \| 3.60E-05 \| 1.80E-02 \| 3.96E-03 \| 1 \| -4.70 \| \| Y \| \| \| \| 57 \| C17orf60 \| 0.82 \| - \| 4.34E-05 \| 2.17E-02 \| 1.78E-04 \| 8.88E-02 \| -1.41 \| \| Y \| \| \| \| 58 \| ZBP1 \| 0.77 \| - \| 4.95E-05 \| 2.48E-02 \| 3.33E-04 \| 1.66E-01 \| -1.90 \| \| Y \| \| \| \| 59 \| FCGR3A \| 0.46 \| - \| 5.34E-05 \| 2.67E-02 \| 4.04E-02 \| 1 \| -6.63 \| \| N \| \| \| \| 60 \| CCL5 \| 0.76 \| - \| 5.62E-05 \| 2.81E-02 \| 8.65E-03 \| 1 \| -5.04 \| \| Y \| \| \| \| 61 \| COCH \| 0.72 \| - \| 6.22E-05 \| 3.11E-02 \| 1.52E-04 \| 7.58E-02 \| -0.89 \| \| Y \| \| \| \| 62 \| STMN2 \| 0.71 \| - \| 6.49E-05 \| 3.25E-02 \| 1.21E-04 \| 6.04E-02 \| -0.62 \| \| N \| \| \| \| 63 \| FERMT3 \| 0.61 \| - \| 6.56E-05 \| 3.28E-02 \| 8.14E-03 \| 1 \| -4.82 \| \| N \| \| \| \| 64 \| LTA \| 0.67 \| - \| 7.96E-05 \| 3.98E-02 \| 4.58E-04 \| 2.29E-01 \| -1.75 \| \| Y \| \| \| \| 65 \| ACAP1 \| 0.86 \| - \| 8.38E-05 \| 4.19E-02 \| 1.52E-04 \| 7.59E-02 \| -0.59 \| \| N \| \| \| \| 66 \| PGAM2 \| 0.86 \| - \| 9.29E-05 \| 4.65E-02 \| 1.15E-10 \| 5.74E-08 \| 13.60 \| \| N \| \| \| \| 67 \| CXCR5 \| 0.82 \| - \| 1.04E-04 \| 5.20E-02 \| 9.56E-04 \| 4.78E-01 \| -2.22 \| \| Y \| \| \| \| 68 \| LOC96610 \| 0.88 \| - \| 1.15E-04 \| 5.77E-02 \| 2.44E-03 \| 1 \| -3.05 \| \| N \| \| \| \| 69 \| CCL4 \| 0.83 \| - \| 1.21E-04 \| 6.04E-02 \| 9.27E-02 \| 1 \| -6.64 \| \| Y \| \| \| \| 70 \| EYA1 \| 0.87 \| - \| 1.23E-04 \| 6.13E-02 \| 4.63E-05 \| 2.31E-02 \| 0.97 \| \| N \| \| \| \| 71 \| LAPTM5 \| 0.56 \| - \| 1.25E-04 \| 6.23E-02 \| 3.75E-02 \| 1 \| -5.71 \| \| N \| \| \| \| 72 \| HEPHL1 \| 0.79 \| - \| 1.27E-04 \| 6.33E-02 \| 3.58E-06 \| 1.79E-03 \| 3.57 \| \| N \| \| \| \| 73 \| XCL2 \| 0.87 \| - \| 1.32E-04 \| 6.58E-02 \| 9.87E-05 \| 4.93E-02 \| 0.29 \| \| Y \| \| \| \| 74 \| PLB1 \| 0.69 \| - \| 1.33E-04 \| 6.63E-02 \| 2.02E-04 \| 1.01E-01 \| -0.42 \| \| N \| \| \| \| 75 \| SAMSN1 \| 0.68 \| - \| 1.35E-04 \| 6.75E-02 \| 2.39E-02 \| 1 \| -5.18 \| \| Y \| \| \| \| 76 \| LOXL3 \| 0.71 \| - \| 1.36E-04 \| 6.81E-02 \| 1.11E-03 \| 5.55E-01 \| -2.10 \| \| N \| \| \| \| 77 \| SH3BP1 \| 0.83 \| - \| 1.42E-04 \| 7.12E-02 \| 2.28E-05 \| 1.14E-02 \| 1.83 \| \| N \| \| \| \| 78 \| MMP12 \| 0.89 \| - \| 1.51E-04 \| 7.57E-02 \| 7.74E-05 \| 3.87E-02 \| 0.67 \| \| N \| \| \| \| 79 \| IL2RA \| 0.62 \| - \| 1.60E-04 \| 7.98E-02 \| 1.10E-02 \| 1 \| -4.23 \| \| Y \| \| \| \| 80 \| GJB6 \| 0.87 \| - \| 1.83E-04 \| 9.13E-02 \| 1.17E-04 \| 5.86E-02 \| 0.44 \| \| N \| \| \| \| 81 \| LOC100188949 \| 0.77 \| - \| 1.89E-04 \| 9.45E-02 \| 1.61E-03 \| 8.07E-01 \| -2.14 \| \| N \| \| \| \| 82 \| LOC283663 \| 0.81 \| - \| 2.06E-04 \| 1.03E-01 \| 4.51E-06 \| 2.25E-03 \| 3.82 \| \| N \| \| \| \| 83 \| HLA-DPA1 \| 0.41 \| + \| 2.10E-04 \| 1.05E-01 \| 6.28E-01 \| 1 \| -8.00 \| \| Y \| \| \| \| 84 \| PLCB2 \| 0.80 \| - \| 2.43E-04 \| 1.21E-01 \| 3.50E-04 \| 1.75E-01 \| -0.37 \| \| N \| \| \| \| 85 \| CRYGS \| 0.56 \| - \| 2.46E-04 \| 1.23E-01 \| 4.82E-09 \| 2.41E-06 \| 10.84 \| \| N \| \| \| \| 86 \| SEZ6L \| 0.70 \| + \| 2.82E-04 \| 1.41E-01 \| 3.83E-04 \| 1.92E-01 \| -0.31 \| \| N \| \| \| \| 87 \| IL9R \| 0.88 \| - \| 3.29E-04 \| 1.65E-01 \| 6.30E-04 \| 3.15E-01 \| -0.65 \| \| Y \| \| \| \| 88 \| MATN4 \| 0.86 \| - \| 3.30E-04 \| 1.65E-01 \| 8.29E-06 \| 4.15E-03 \| 3.68 \| \| N \| \| \| \| 89 \| SIGLEC1 \| 0.72 \| - \| 3.46E-04 \| 1.73E-01 \| 2.61E-03 \| 1 \| -2.02 \| \| Y \| \| \| \| 90 \| HAS1 \| 0.89 \| - \| 3.63E-04 \| 1.81E-01 \| 1.81E-06 \| 9.07E-04 \| 5.30 \| \| Y \| \| \| \| 91 \| GPR171 \| 0.90 \| - \| 3.69E-04 \| 1.84E-01 \| 1.03E-02 \| 1 \| -3.33 \| \| N \| \| \| \| 92 \| VSIG4 \| 0.62 \| - \| 3.84E-04 \| 1.92E-01 \| 2.50E-02 \| 1 \| -4.17 \| \| Y \| \| \| \| 93 \| F3 \| 0.75 \| - \| 3.85E-04 \| 1.92E-01 \| 1.27E-03 \| 6.36E-01 \| -1.20 \| \| Y \| \| \| \| 94 \| JAKMIP1 \| 0.79 \| - \| 4.06E-04 \| 2.03E-01 \| 1.11E-02 \| 1 \| -3.31 \| \| N \| \| \| \| 95 \| IRF4 \| 0.75 \| - \| 4.25E-04 \| 2.12E-01 \| 1.17E-02 \| 1 \| -3.31 \| \| Y \| \| \| \| 96 \| CORO1A \| 0.66 \| - \| 4.27E-04 \| 2.14E-01 \| 9.36E-03 \| 1 \| -3.09 \| \| Y \| \| \| \| 97 \| MGC29506 \| 0.89 \| - \| 4.41E-04 \| 2.20E-01 \| 5.39E-04 \| 2.69E-01 \| -0.20 \| \| N \| \| \| \| 98 \| VAV1 \| 0.44 \| - \| 4.46E-04 \| 2.23E-01 \| 7.42E-02 \| 1 \| -5.11 \| \| Y \| \| \| \| 99 \| C1QC \| 0.47 \| - \| 4.66E-04 \| 2.33E-01 \| 5.33E-03 \| 1 \| -2.44 \| \| Y \| \| \| \| 100 \| RGS1 \| 0.78 \| - \| 5.40E-04 \| 2.70E-01 \| 7.09E-03 \| 1 \| -2.58 \| \| Y \| \| \| \| 101 \| CD37 \| 0.65 \| - \| 5.51E-04 \| 2.76E-01 \| 2.15E-02 \| 1 \| -3.66 \| \| Y \| \| \| \| 102 \| LILRA2 \| 0.80 \| + \| 5.76E-04 \| 2.88E-01 \| 1.18E-01 \| 1 \| -5.33 \| \| Y \| \| \| \| 103 \| PIM2 \| 0.73 \| - \| 6.15E-04 \| 3.08E-01 \| 1.34E-04 \| 6.69E-02 \| 1.53 \| \| N \| \| \| \| 104 \| TNFAIP8L2 \| 0.78 \| - \| 6.21E-04 \| 3.10E-01 \| 1.71E-03 \| 8.57E-01 \| -1.01 \| \| Y \| \| \| \| 105 \| MAP4K1 \| 0.78 \| - \| 6.23E-04 \| 3.12E-01 \| 6.20E-04 \| 3.10E-01 \| 0.01 \| \| Y \| \| \| \| 106 \| CD274 \| 0.58 \| + \| 6.34E-04 \| 3.17E-01 \| 2.24E-01 \| 1 \| -5.87 \| \| Y \| \| \| \| 107 \| SIGLEC10 \| 0.44 \| - \| 6.54E-04 \| 3.27E-01 \| 1.68E-01 \| 1 \| -5.55 \| \| Y \| \| \| \| 108 \| GBP5 \| 0.56 \| - \| 6.59E-04 \| 3.29E-01 \| 3.52E-02 \| 1 \| -3.98 \| \| Y \| \| \| \| 109 \| TTC24 \| 0.87 \| - \| 6.99E-04 \| 3.49E-01 \| 3.33E-03 \| 1 \| -1.56 \| \| N \| \| \| \| 110 \| PDCD1 \| 0.75 \| - \| 7.28E-04 \| 3.64E-01 \| 1.92E-02 \| 1 \| -3.27 \| \| Y \| \| \| \| 111 \| ITGA2B \| 0.59 \| - \| 8.63E-04 \| 4.31E-01 \| 5.94E-06 \| 2.97E-03 \| 4.98 \| \| Y \| \| \| \| 112 \| MYO1G \| 0.88 \| - \| 9.02E-04 \| 4.51E-01 \| 3.60E-03 \| 1 \| -1.38 \| \| N \| \| \| \| 113 \| PIK3CG \| 0.75 \| + \| 9.45E-04 \| 4.72E-01 \| 6.95E-02 \| 1 \| -4.30 \| \| N \| \| \| \| 114 \| CCR4 \| 0.68 \| + \| 9.79E-04 \| 4.90E-01 \| 1.07E-01 \| 1 \| -4.69 \| \| Y \| \| \| \| 115 \| ZPLD1 \| 0.67 \| - \| 1.01E-03 \| 5.05E-01 \| 1.24E-03 \| 6.19E-01 \| -0.20 \| \| N \| \| \| \| 116 \| TBC1D10C \| 0.86 \| - \| 1.12E-03 \| 5.62E-01 \| 5.44E-04 \| 2.72E-01 \| 0.72 \| \| N \| \| \| \| 117 \| CD3D \| 0.71 \| - \| 1.13E-03 \| 5.63E-01 \| 1.39E-01 \| 1 \| -4.82 \| \| Y \| \| \| \| 118 \| GYPA \| 0.86 \| + \| 1.24E-03 \| 6.19E-01 \| 2.70E-04 \| 1.35E-01 \| 1.52 \| \| Y \| \| \| \| 119 \| C12orf70 \| 0.76 \| - \| 1.28E-03 \| 6.38E-01 \| 2.44E-04 \| 1.22E-01 \| 1.65 \| \| N \| \| \| \| 120 \| MPEG1 \| 0.66 \| + \| 1.51E-03 \| 7.57E-01 \| 6.44E-02 \| 1 \| -3.75 \| \| N \| \| \| \| 121 \| GPR34 \| 0.74 \| + \| 1.59E-03 \| 7.94E-01 \| 3.79E-02 \| 1 \| -3.17 \| \| N \| \| \| \| 122 \| TMC8 \| 0.80 \| - \| 1.65E-03 \| 8.27E-01 \| 1.40E-04 \| 7.02E-02 \| 2.47 \| \| N \| \| \| \| 123 \| CASP5 \| 0.61 \| - \| 1.76E-03 \| 8.79E-01 \| 1.02E-03 \| 5.12E-01 \| 0.54 \| \| Y \| \| \| \| 124 \| C1orf162 \| 0.75 \| - \| 1.78E-03 \| 8.88E-01 \| 5.42E-03 \| 1 \| -1.12 \| \| N \| \| \| \| 125 \| SLA2 \| 0.63 \| - \| 2.09E-03 \| 1 \| 1.65E-02 \| 1 \| -2.06 \| \| Y \| \| \| \| 126 \| C13orf18 \| 0.79 \| - \| 2.25E-03 \| 1 \| 2.87E-02 \| 1 \| -2.55 \| \| N \| \| \| \| 127 \| P2RY13 \| 0.74 \| + \| 2.26E-03 \| 1 \| 1.10E-01 \| 1 \| -3.88 \| \| N \| \| \| \| 128 \| IL21R \| 0.69 \| - \| 2.34E-03 \| 1 \| 4.53E-02 \| 1 \| -2.96 \| \| Y \| \| \| \| 129 \| OPN4 \| 0.80 \| - \| 2.55E-03 \| 1 \| 1.30E-03 \| 6.49E-01 \| 0.68 \| \| N \| \| \| \| 130 \| SIGLEC16 \| 0.70 \| - \| 2.61E-03 \| 1 \| 3.28E-04 \| 1.64E-01 \| 2.07 \| \| N \| \| \| \| 131 \| SCARNA5 \| 0.84 \| - \| 2.64E-03 \| 1 \| 6.77E-04 \| 3.38E-01 \| 1.36 \| \| N \| \| \| \| 132 \| IGSF6 \| 0.57 \| + \| 2.69E-03 \| 1 \| 5.99E-01 \| 1 \| -5.41 \| \| Y \| \| \| \| 133 \| CECR1 \| 0.77 \| + \| 2.77E-03 \| 1 \| 3.70E-01 \| 1 \| -4.89 \| \| N \| \| \| \| 134 \| C19orf59 \| 0.74 \| - \| 2.80E-03 \| 1 \| 8.74E-03 \| 1 \| -1.14 \| \| N \| \| \| \| 135 \| GNG4 \| 0.89 \| - \| 2.90E-03 \| 1 \| 3.53E-03 \| 1 \| -0.20 \| \| N \| \| \| \| 136 \| UBASH3A \| 0.66 \| - \| 3.12E-03 \| 1 \| 4.85E-02 \| 1 \| -2.74 \| \| N \| \| \| \| 137 \| POU2AF1 \| 0.90 \| - \| 3.19E-03 \| 1 \| 2.16E-03 \| 1 \| 0.39 \| \| Y \| \| \| \| 138 \| HTRA4 \| 0.85 \| - \| 3.21E-03 \| 1 \| 9.55E-03 \| 1 \| -1.09 \| \| N \| \| \| \| 139 \| CD3E \| 0.67 \| - \| 3.54E-03 \| 1 \| 1.38E-01 \| 1 \| -3.66 \| \| Y \| \| \| \| 140 \| SIRPG \| 0.73 \| - \| 3.77E-03 \| 1 \| 1.58E-01 \| 1 \| -3.74 \| \| N \| \| \| \| 141 \| CCL22 \| 0.82 \| + \| 3.93E-03 \| 1 \| 1.24E-02 \| 1 \| -1.15 \| \| Y \| \| \| \| 142 \| IL12RB1 \| 0.50 \| - \| 4.09E-03 \| 1 \| 9.30E-02 \| 1 \| -3.12 \| \| Y \| \| \| \| 143 \| GBP4 \| 0.82 \| + \| 4.17E-03 \| 1 \| 2.83E-02 \| 1 \| -1.91 \| \| Y \| \| \| \| 144 \| SPIC \| 0.84 \| + \| 4.35E-03 \| 1 \| 1.16E-01 \| 1 \| -3.29 \| \| N \| \| \| \| 145 \| P2RY12 \| 0.77 \| + \| 4.42E-03 \| 1 \| 1.09E-01 \| 1 \| -3.21 \| \| N \| \| \| \| 146 \| CD226 \| 0.70 \| + \| 4.92E-03 \| 1 \| 1.24E-01 \| 1 \| -3.23 \| \| Y \| \| \| \| 147 \| SASH3 \| 0.46 \| - \| 5.36E-03 \| 1 \| 1.76E-01 \| 1 \| -3.49 \| \| Y \| \| \| \| 148 \| CRYBB1 \| 0.81 \| - \| 5.41E-03 \| 1 \| 2.81E-04 \| 1.40E-01 \| 2.96 \| \| N \| \| \| \| 149 \| TIGIT \| 0.67 \| - \| 5.45E-03 \| 1 \| 2.19E-02 \| 1 \| -1.39 \| \| N \| \| \| \| 150 \| MSMP \| 0.64 \| + \| 5.63E-03 \| 1 \| 4.64E-05 \| 2.32E-02 \| 4.80 \| \| N \| \| \| \| 151 \| TNFSF8 \| 0.60 \| + \| 5.71E-03 \| 1 \| 2.04E-01 \| 1 \| -3.58 \| \| Y \| \| \| \| 152 \| LOC100233209 \| 0.63 \| - \| 5.89E-03 \| 1 \| 8.04E-02 \| 1 \| -2.61 \| \| N \| \| \| \| 153 \| FAM26F \| 0.65 \| - \| 6.01E-03 \| 1 \| 4.07E-03 \| 1 \| 0.39 \| \| N \| \| \| \| 154 \| CD79A \| 0.90 \| - \| 6.02E-03 \| 1 \| 3.01E-02 \| 1 \| -1.61 \| \| Y \| \| \| \| 155 \| GPR18 \| 0.68 \| - \| 6.14E-03 \| 1 \| 1.35E-02 \| 1 \| -0.79 \| \| N \| \| \| \| 156 \| CD101 \| 0.79 \| - \| 6.27E-03 \| 1 \| 3.71E-03 \| 1 \| 0.53 \| \| Y \| \| \| \| 157 \| TRAF3IP3 \| 0.63 \| - \| 6.28E-03 \| 1 \| 1.52E-02 \| 1 \| -0.89 \| \| N \| \| \| \| 158 \| FCGR2B \| 0.68 \| - \| 6.67E-03 \| 1 \| 5.05E-02 \| 1 \| -2.03 \| \| N \| \| \| \| 159 \| RNASE3 \| 0.81 \| - \| 6.71E-03 \| 1 \| 5.00E-03 \| 1 \| 0.29 \| \| Y \| \| \| \| 160 \| RORB \| 0.84 \| - \| 6.92E-03 \| 1 \| 7.15E-03 \| 1 \| -0.03 \| \| N \| \| \| \| 161 \| KEL \| 0.78 \| - \| 7.15E-03 \| 1 \| 3.50E-02 \| 1 \| -1.59 \| \| Y \| \| \| \| 162 \| FASLG \| 0.66 \| - \| 7.34E-03 \| 1 \| 3.22E-02 \| 1 \| -1.48 \| \| Y \| \| \| \| 163 \| SYT5 \| 0.84 \| - \| 7.36E-03 \| 1 \| 3.86E-04 \| 1.93E-01 \| 2.95 \| \| N \| \| \| \| 164 \| CST7 \| 0.67 \| - \| 7.37E-03 \| 1 \| 9.67E-02 \| 1 \| -2.57 \| \| Y \| \| \| \| 165 \| EMILIN3 \| 0.90 \| - \| 7.37E-03 \| 1 \| 3.12E-03 \| 1 \| 0.86 \| \| N \| \| \| \| 166 \| IL7R \| 0.90 \| + \| 7.46E-03 \| 1 \| 2.09E-01 \| 1 \| -3.33 \| \| Y \| \| \| \| 167 \| TLR7 \| 0.59 \| + \| 7.47E-03 \| 1 \| 2.06E-01 \| 1 \| -3.32 \| \| Y \| \| \| \| 168 \| PTPN22 \| 0.64 \| - \| 7.95E-03 \| 1 \| 6.13E-02 \| 1 \| -2.04 \| \| Y \| \| \| \| 169 \| KLRK1 \| 0.84 \| - \| 8.41E-03 \| 1 \| 1.13E-01 \| 1 \| -2.60 \| \| Y \| \| \| \| 170 \| OTOA \| 0.86 \| + \| 8.49E-03 \| 1 \| 1.33E-02 \| 1 \| -0.45 \| \| N \| \| \| \| 171 \| CD27 \| 0.73 \| - \| 8.58E-03 \| 1 \| 4.06E-02 \| 1 \| -1.55 \| \| Y \| \| \| \| 172 \| PTPRZ1 \| 0.82 \| - \| 8.59E-03 \| 1 \| 3.75E-02 \| 1 \| -1.48 \| \| N \| \| \| \| 173 \| LAT2 \| 0.72 \| - \| 8.64E-03 \| 1 \| 2.22E-02 \| 1 \| -0.94 \| \| Y \| \| \| \| 174 \| ICOS \| 0.66 \| - \| 8.96E-03 \| 1 \| 1.21E-01 \| 1 \| -2.60 \| \| Y \| \| \| \| 175 \| CR1 \| 0.85 \| - \| 9.21E-03 \| 1 \| 9.42E-02 \| 1 \| -2.33 \| \| Y \| \| \| \| 176 \| PTPRCAP \| 0.87 \| - \| 9.41E-03 \| 1 \| 2.80E-02 \| 1 \| -1.09 \| \| Y \| \| \| \| 177 \| SNORD17 \| 0.82 \| - \| 9.89E-03 \| 1 \| 4.44E-03 \| 1 \| 0.80 \| \| N \| \| \| \| 178 \| HLA-DPB1 \| 0.44 \| + \| 9.91E-03 \| 1 \| 6.95E-01 \| 1 \| -4.25 \| \| Y \| \| \| \| 179 \| GBP1 \| 0.75 \| - \| 9.92E-03 \| 1 \| 1.39E-01 \| 1 \| -2.64 \| \| Y \| \| \| \| 180 \| PLXNC1 \| 0.81 \| + \| 1.01E-02 \| 1 \| 2.04E-02 \| 1 \| -0.70 \| \| N \| \| \| \| 181 \| CD53 \| 0.37 \| - \| 1.03E-02 \| 1 \| 1.83E-01 \| 1 \| -2.87 \| \| Y \| \| \| \| 182 \| HLA-DQB2 \| 0.88 \| + \| 1.04E-02 \| 1 \| 2.67E-01 \| 1 \| -3.25 \| \| Y \| \| \| \| 183 \| HLA-DOB \| 0.76 \| - \| 1.04E-02 \| 1 \| 1.85E-02 \| 1 \| -0.57 \| \| Y \| \| \| \| 184 \| CEL \| 0.68 \| - \| 1.05E-02 \| 1 \| 2.62E-04 \| 1.31E-01 \| 3.69 \| \| N \| \| \| \| 185 \| CD96 \| 0.61 \| - \| 1.06E-02 \| 1 \| 2.18E-01 \| 1 \| -3.02 \| \| N \| \| \| \| 186 \| WDFY4 \| 0.68 \| + \| 1.07E-02 \| 1 \| 1.62E-01 \| 1 \| -2.72 \| \| N \| \| \| \| 187 \| SAMD3 \| 0.87 \| - \| 1.11E-02 \| 1 \| 3.63E-02 \| 1 \| -1.19 \| \| N \| \| \| \| 188 \| CLEC9A \| 0.76 \| + \| 1.15E-02 \| 1 \| 5.24E-02 \| 1 \| -1.52 \| \| N \| \| \| \| 189 \| CD300LB \| 0.81 \| - \| 1.15E-02 \| 1 \| 4.34E-02 \| 1 \| -1.33 \| \| Y \| \| \| \| 190 \| MNDA \| 0.64 \| + \| 1.16E-02 \| 1 \| 6.87E-01 \| 1 \| -4.09 \| \| Y \| \| \| \| 191 \| PARP15 \| 0.84 \| - \| 1.19E-02 \| 1 \| 5.54E-03 \| 1 \| 0.76 \| \| N \| \| \| \| 192 \| CX3CR1 \| 0.85 \| + \| 1.24E-02 \| 1 \| 4.91E-03 \| 1 \| 0.92 \| \| Y \| \| \| \| 193 \| CXCR3 \| 0.68 \| - \| 1.24E-02 \| 1 \| 2.31E-02 \| 1 \| -0.63 \| \| Y \| \| \| \| 194 \| CLEC7A \| 0.63 \| - \| 1.34E-02 \| 1 \| 2.16E-01 \| 1 \| -2.78 \| \| Y \| \| \| \| 195 \| IL1A \| 0.68 \| - \| 1.36E-02 \| 1 \| 9.22E-03 \| 1 \| 0.39 \| \| Y \| \| \| \| 196 \| SELPLG \| 0.63 \| - \| 1.43E-02 \| 1 \| 2.01E-02 \| 1 \| -0.34 \| \| Y \| \| \| \| 197 \| SIGLEC8 \| 0.64 \| + \| 1.46E-02 \| 1 \| 8.98E-02 \| 1 \| -1.82 \| \| Y \| \| \| \| 198 \| TNFRSF13B \| 0.85 \| - \| 1.52E-02 \| 1 \| 1.71E-02 \| 1 \| -0.12 \| \| Y \| \| \| \| 199 \| KIAA0125 \| 0.73 \| - \| 1.62E-02 \| 1 \| 1.54E-02 \| 1 \| 0.05 \| \| N \| \| \| \| 200 \| AKNA \| 0.85 \| - \| 1.63E-02 \| 1 \| 1.80E-02 \| 1 \| -0.10 \| \| N \| \| \| \| 201 \| SLA \| 0.55 \| - \| 1.64E-02 \| 1 \| 3.20E-01 \| 1 \| -2.97 \| \| N \| \| \| \| 202 \| CYP2E1 \| 0.69 \| - \| 1.65E-02 \| 1 \| 1.57E-03 \| 7.86E-01 \| 2.35 \| \| N \| \| \| \| 203 \| NFAM1 \| 0.76 \| - \| 1.68E-02 \| 1 \| 2.09E-01 \| 1 \| -2.52 \| \| Y \| \| \| \| 204 \| SNORD10 \| 0.80 \| - \| 1.68E-02 \| 1 \| 2.21E-04 \| 1.10E-01 \| 4.33 \| \| N \| \| \| \| 205 \| TREM2 \| 0.78 \| - \| 1.86E-02 \| 1 \| 5.71E-02 \| 1 \| -1.12 \| \| Y \| \| \| \| 206 \| CCDC91 \| 0.83 \| - \| 1.88E-02 \| 1 \| 4.25E-06 \| 2.13E-03 \| 8.39 \| \| N \| \| \| \| 207 \| LYZ \| 0.70 \| + \| 1.89E-02 \| 1 \| 9.18E-02 \| 1 \| -1.58 \| \| Y \| \| \| \| 208 \| CXCR6 \| 0.61 \| - \| 1.90E-02 \| 1 \| 1.10E-01 \| 1 \| -1.75 \| \| Y \| \| \| \| 209 \| FOSL1 \| 0.88 \| - \| 2.03E-02 \| 1 \| 1.00E-03 \| 5.00E-01 \| 3.01 \| \| N \| \| \| \| 210 \| LILRB4 \| 0.58 \| - \| 2.04E-02 \| 1 \| 8.61E-02 \| 1 \| -1.44 \| \| Y \| \| \| \| 211 \| IL10 \| 0.87 \| - \| 2.07E-02 \| 1 \| 1.17E-01 \| 1 \| -1.73 \| \| Y \| \| \| \| 212 \| NDRG4 \| 0.85 \| - \| 2.11E-02 \| 1 \| 4.88E-04 \| 2.44E-01 \| 3.77 \| \| N \| \| \| \| 213 \| P2RX5 \| 0.85 \| - \| 2.19E-02 \| 1 \| 1.53E-02 \| 1 \| 0.36 \| \| N \| \| \| \| 214 \| CTSS \| 0.49 \| + \| 2.25E-02 \| 1 \| 8.14E-01 \| 1 \| -3.59 \| \| Y \| \| \| \| 215 \| GFI1 \| 0.82 \| - \| 2.33E-02 \| 1 \| 1.63E-02 \| 1 \| 0.36 \| \| Y \| \| \| \| 216 \| C8orf46 \| 0.91 \| - \| 2.38E-02 \| 1 \| 8.24E-03 \| 1 \| 1.06 \| \| N \| \| \| \| 217 \| FPR3 \| 0.60 \| + \| 2.50E-02 \| 1 \| 6.37E-01 \| 1 \| -3.24 \| \| Y \| \| \| \| 218 \| CSF1R \| 0.57 \| - \| 2.52E-02 \| 1 \| 2.77E-01 \| 1 \| -2.40 \| \| Y \| \| \| \| 219 \| CLEC12B \| 0.83 \| - \| 2.57E-02 \| 1 \| 2.58E-02 \| 1 \| 0.00 \| \| N \| \| \| \| 220 \| C1QL3 \| 0.88 \| + \| 2.58E-02 \| 1 \| 1.26E-02 \| 1 \| 0.72 \| \| N \| \| \| \| 221 \| LILRA1 \| 0.84 \| + \| 2.59E-02 \| 1 \| 1.14E-01 \| 1 \| -1.48 \| \| Y \| \| \| \| 222 \| PLD4 \| 0.77 \| + \| 2.73E-02 \| 1 \| 1.03E-01 \| 1 \| -1.33 \| \| N \| \| \| \| 223 \| GZMA \| 0.72 \| - \| 2.76E-02 \| 1 \| 7.79E-02 \| 1 \| -1.04 \| \| Y \| \| \| \| 224 \| PTPRN \| 0.81 \| - \| 2.87E-02 \| 1 \| 1.27E-02 \| 1 \| 0.81 \| \| N \| \| \| \| 225 \| HLA-DOA \| 0.59 \| + \| 2.92E-02 \| 1 \| 3.14E-01 \| 1 \| -2.38 \| \| Y \| \| \| \| 226 \| GAPDHS \| 0.72 \| - \| 2.98E-02 \| 1 \| 3.06E-04 \| 1.53E-01 \| 4.58 \| \| N \| \| \| \| 227 \| WDR69 \| 0.90 \| - \| 2.99E-02 \| 1 \| 3.12E-03 \| 1 \| 2.26 \| \| N \| \| \| \| 228 \| SH2D4B \| 0.90 \| - \| 3.00E-02 \| 1 \| 7.07E-02 \| 1 \| -0.86 \| \| N \| \| \| \| 229 \| RHOH \| 0.61 \| - \| 3.07E-02 \| 1 \| 2.75E-01 \| 1 \| -2.20 \| \| N \| \| \| \| 230 \| RASSF5 \| 0.63 \| - \| 3.22E-02 \| 1 \| 1.58E-01 \| 1 \| -1.59 \| \| N \| \| \| \| 231 \| LCK \| 0.61 \| - \| 3.23E-02 \| 1 \| 3.83E-01 \| 1 \| -2.47 \| \| Y \| \| \| \| 232 \| ADAMTS6 \| 0.87 \| - \| 3.44E-02 \| 1 \| 4.14E-02 \| 1 \| -0.19 \| \| N \| \| \| \| 233 \| CYBB \| 0.57 \| + \| 3.64E-02 \| 1 \| 4.42E-01 \| 1 \| -2.50 \| \| Y \| \| \| \| 234 \| CD84 \| 0.67 \| + \| 3.79E-02 \| 1 \| 1.06E-01 \| 1 \| -1.03 \| \| Y \| \| \| \| 235 \| TNFRSF9 \| 0.80 \| - \| 3.85E-02 \| 1 \| 1.38E-01 \| 1 \| -1.27 \| \| Y \| \| \| \| 236 \| CD33 \| 0.57 \| - \| 3.87E-02 \| 1 \| 5.48E-01 \| 1 \| -2.65 \| \| Y \| \| \| \| 237 \| DHRS9 \| 0.78 \| + \| 3.89E-02 \| 1 \| 3.43E-01 \| 1 \| -2.18 \| \| N \| \| \| \| 238 \| CD2 \| 0.62 \| - \| 3.93E-02 \| 1 \| 1.55E-01 \| 1 \| -1.37 \| \| Y \| \| \| \| 239 \| NCF1B \| 0.91 \| - \| 4.02E-02 \| 1 \| 4.91E-02 \| 1 \| -0.20 \| \| N \| \| \| \| 240 \| GTSF1L \| 0.87 \| - \| 4.38E-02 \| 1 \| 7.56E-02 \| 1 \| -0.55 \| \| N \| \| \| \| 241 \| SIGLEC11 \| 0.69 \| - \| 4.43E-02 \| 1 \| 1.34E-02 \| 1 \| 1.19 \| \| Y \| \| \| \| 242 \| FPR2 \| 0.71 \| - \| 4.58E-02 \| 1 \| 1.18E-01 \| 1 \| -0.95 \| \| Y \| \| \| \| 243 \| KIF21B \| 0.81 \| - \| 4.64E-02 \| 1 \| 4.47E-01 \| 1 \| -2.27 \| \| N \| \| \| \| 244 \| SAMHD1 \| 0.70 \| + \| 4.65E-02 \| 1 \| 6.70E-02 \| 1 \| -0.37 \| \| Y \| \| \| \| 245 \| ADAMDEC1 \| 0.78 \| - \| 4.65E-02 \| 1 \| 1.21E-01 \| 1 \| -0.95 \| \| Y \| \| \| \| 246 \| DRD2 \| 0.86 \| - \| 4.74E-02 \| 1 \| 7.95E-03 \| 1 \| 1.78 \| \| N \| \| \| \| 247 \| CLEC5A \| 0.86 \| - \| 4.77E-02 \| 1 \| 8.54E-02 \| 1 \| -0.58 \| \| Y \| \| \| \| 248 \| F13A1 \| 0.82 \| - \| 4.85E-02 \| 1 \| 7.86E-02 \| 1 \| -0.48 \| \| Y \| \| \| \| 249 \| NCF1 \| 0.84 \| - \| 4.86E-02 \| 1 \| 1.54E-01 \| 1 \| -1.16 \| \| Y \| \| \| \| 250 \| CD5L \| 0.88 \| + \| 4.86E-02 \| 1 \| 1.91E-01 \| 1 \| -1.37 \| \| Y \| \| \| \| 251 \| GPR65 \| 0.40 \| - \| 4.90E-02 \| 1 \| 3.08E-01 \| 1 \| -1.84 \| \| Y \| \| \| \| 252 \| NKG7 \| 0.77 \| - \| 5.00E-02 \| 1 \| 1.16E-01 \| 1 \| -0.84 \| \| Y \| \| \| \| 253 \| CTSW \| 0.79 \| - \| 5.03E-02 \| 1 \| 4.04E-02 \| 1 \| 0.22 \| \| Y \| \| \| \| 254 \| IL2RB \| 0.78 \| - \| 5.07E-02 \| 1 \| 5.75E-03 \| 1 \| 2.18 \| \| Y \| \| \| \| 255 \| MRC1 \| 0.81 \| + \| 5.41E-02 \| 1 \| 5.64E-02 \| 1 \| -0.04 \| \| Y \| \| \| \| 256 \| SOSTDC1 \| 0.84 \| + \| 5.44E-02 \| 1 \| 4.78E-03 \| 1 \| 2.43 \| \| N \| \| \| \| 257 \| GAL3ST4 \| 0.60 \| - \| 5.66E-02 \| 1 \| 2.72E-02 \| 1 \| 0.73 \| \| N \| \| \| \| 258 \| C5AR1 \| 0.86 \| - \| 5.75E-02 \| 1 \| 1.90E-01 \| 1 \| -1.20 \| \| Y \| \| \| \| 259 \| LCP2 \| 0.60 \| - \| 5.78E-02 \| 1 \| 1.72E-01 \| 1 \| -1.09 \| \| Y \| \| \| \| 260 \| TWIST2 \| 0.86 \| - \| 5.80E-02 \| 1 \| 7.22E-02 \| 1 \| -0.22 \| \| N \| \| \| \| 261 \| GZMK \| 0.68 \| - \| 5.89E-02 \| 1 \| 2.83E-01 \| 1 \| -1.57 \| \| Y \| \| \| \| 262 \| HLA-G \| 0.87 \| + \| 5.91E-02 \| 1 \| 2.11E-01 \| 1 \| -1.27 \| \| Y \| \| \| \| 263 \| ITGAL \| 0.56 \| - \| 5.99E-02 \| 1 \| 1.97E-01 \| 1 \| -1.19 \| \| Y \| \| \| \| 264 \| CD86 \| 0.43 \| - \| 6.19E-02 \| 1 \| 1.35E-01 \| 1 \| -0.78 \| \| Y \| \| \| \| 265 \| SLAMF7 \| 0.65 \| - \| 6.42E-02 \| 1 \| 2.09E-01 \| 1 \| -1.18 \| \| Y \| \| \| \| 266 \| CD6 \| 0.75 \| - \| 6.48E-02 \| 1 \| 1.74E-01 \| 1 \| -0.99 \| \| Y \| \| \| \| 267 \| TAS1R3 \| 0.83 \| - \| 6.50E-02 \| 1 \| 1.05E-02 \| 1 \| 1.82 \| \| N \| \| \| \| 268 \| C1orf200 \| 0.86 \| - \| 6.56E-02 \| 1 \| 6.80E-03 \| 1 \| 2.27 \| \| N \| \| \| \| 269 \| CD247 \| 0.76 \| - \| 6.86E-02 \| 1 \| 7.52E-02 \| 1 \| -0.09 \| \| N \| \| \| \| 270 \| NCF2 \| 0.82 \| + \| 7.19E-02 \| 1 \| 5.40E-01 \| 1 \| -2.02 \| \| Y \| \| \| \| 271 \| TLR8 \| 0.47 \| + \| 7.22E-02 \| 1 \| 4.61E-01 \| 1 \| -1.85 \| \| Y \| \| \| \| 272 \| LIPG \| 0.85 \| - \| 7.30E-02 \| 1 \| 1.35E-01 \| 1 \| -0.62 \| \| N \| \| \| \| 273 \| HLA-DQA1 \| 0.53 \| + \| 7.32E-02 \| 1 \| 4.81E-01 \| 1 \| -1.88 \| \| Y \| \| \| \| 274 \| SIGLEC15 \| 0.89 \| - \| 7.36E-02 \| 1 \| 4.78E-02 \| 1 \| 0.43 \| \| N \| \| \| \| 275 \| SLAMF1 \| 0.69 \| - \| 7.45E-02 \| 1 \| 2.58E-02 \| 1 \| 1.06 \| \| Y \| \| \| \| 276 \| SIGLEC12 \| 0.63 \| - \| 7.67E-02 \| 1 \| 1.77E-02 \| 1 \| 1.46 \| \| N \| \| \| \| 277 \| SLFN12L \| 0.64 \| - \| 7.67E-02 \| 1 \| 4.09E-01 \| 1 \| -1.67 \| \| N \| \| \| \| 278 \| DOK2 \| 0.70 \| - \| 7.75E-02 \| 1 \| 7.50E-02 \| 1 \| 0.03 \| \| N \| \| \| \| 279 \| VPREB3 \| 0.74 \| - \| 8.00E-02 \| 1 \| 6.34E-03 \| 1 \| 2.54 \| \| N \| \| \| \| 280 \| SIGLEC9 \| 0.61 \| - \| 8.02E-02 \| 1 \| 4.47E-01 \| 1 \| -1.72 \| \| Y \| \| \| \| 281 \| IKZF3 \| 0.59 \| + \| 8.04E-02 \| 1 \| 2.57E-01 \| 1 \| -1.16 \| \| Y \| \| \| \| 282 \| SLC37A2 \| 0.65 \| + \| 8.12E-02 \| 1 \| 1.69E-01 \| 1 \| -0.73 \| \| N \| \| \| \| 283 \| THBS4 \| 0.85 \| - \| 8.18E-02 \| 1 \| 1.30E-01 \| 1 \| -0.46 \| \| N \| \| \| \| 284 \| SIRPB2 \| 0.80 \| - \| 8.20E-02 \| 1 \| 5.43E-02 \| 1 \| 0.41 \| \| N \| \| \| \| 285 \| PYHIN1 \| 0.67 \| - \| 8.23E-02 \| 1 \| 8.01E-02 \| 1 \| 0.03 \| \| N \| \| \| \| 286 \| CD3G \| 0.56 \| + \| 8.28E-02 \| 1 \| 9.70E-01 \| 1 \| -2.46 \| \| Y \| \| \| \| 287 \| HBG2 \| 0.87 \| + \| 8.43E-02 \| 1 \| 7.51E-02 \| 1 \| 0.12 \| \| N \| \| \| \| 288 \| FGD2 \| 0.78 \| - \| 8.90E-02 \| 1 \| 5.92E-01 \| 1 \| -1.90 \| \| N \| \| \| \| 289 \| HBA2 \| 0.82 \| + \| 8.91E-02 \| 1 \| 2.25E-01 \| 1 \| -0.93 \| \| N \| \| \| \| 290 \| CD8A \| 0.67 \| - \| 9.15E-02 \| 1 \| 2.04E-01 \| 1 \| -0.80 \| \| Y \| \| \| \| 291 \| CD52 \| 0.78 \| - \| 9.27E-02 \| 1 \| 9.90E-02 \| 1 \| -0.07 \| \| Y \| \| \| \| 292 \| GVIN1 \| 0.88 \| + \| 9.34E-02 \| 1 \| 3.92E-02 \| 1 \| 0.87 \| \| N \| \| \| \| 293 \| AQPEP \| 0.65 \| - \| 9.40E-02 \| 1 \| 2.34E-02 \| 1 \| 1.39 \| \| N \| \| \| \| 294 \| NOG \| 0.82 \| - \| 9.47E-02 \| 1 \| 2.98E-02 \| 1 \| 1.16 \| \| N \| \| \| \| 295 \| PAX6 \| 0.82 \| + \| 9.59E-02 \| 1 \| 1.91E-01 \| 1 \| -0.69 \| \| N \| \| \| \| 296 \| AOAH \| 0.47 \| + \| 9.63E-02 \| 1 \| 6.87E-01 \| 1 \| -1.96 \| \| N \| \| \| \| 297 \| HCK \| 0.69 \| - \| 9.84E-02 \| 1 \| 1.68E-01 \| 1 \| -0.53 \| \| Y \| \| \| \| 298 \| PHACTR3 \| 0.88 \| - \| 9.88E-02 \| 1 \| 6.49E-02 \| 1 \| 0.42 \| \| N \| \| \| \| 299 \| CD38 \| 0.65 \| - \| 1.01E-01 \| 1 \| 2.97E-01 \| 1 \| -1.08 \| \| Y \| \| \| \| 300 \| GIMAP2 \| 0.78 \| + \| 1.01E-01 \| 1 \| 1.10E-01 \| 1 \| -0.09 \| \| N \| \| \| \| 301 \| SLC1A3 \| 0.68 \| + \| 1.04E-01 \| 1 \| 5.70E-01 \| 1 \| -1.70 \| \| N \| \| \| \| 302 \| LOC441869 \| 0.88 \| + \| 1.05E-01 \| 1 \| 4.16E-02 \| 1 \| 0.92 \| \| N \| \| \| \| 303 \| KLRC4 \| 0.85 \| - \| 1.05E-01 \| 1 \| 4.00E-02 \| 1 \| 0.96 \| \| Y \| \| \| \| 304 \| FCGR2C \| 0.63 \| - \| 1.07E-01 \| 1 \| 6.86E-02 \| 1 \| 0.44 \| \| Y \| \| \| \| 305 \| ZDHHC8P1 \| 0.90 \| - \| 1.08E-01 \| 1 \| 3.30E-02 \| 1 \| 1.19 \| \| N \| \| \| \| 306 \| LOC100192379 \| 0.76 \| - \| 1.09E-01 \| 1 \| 2.84E-01 \| 1 \| -0.96 \| \| N \| \| \| \| 307 \| BLK \| 0.88 \| - \| 1.10E-01 \| 1 \| 1.11E-01 \| 1 \| 0.00 \| \| Y \| \| \| \| 308 \| CD74 \| 0.71 \| - \| 1.11E-01 \| 1 \| 2.99E-01 \| 1 \| -1.00 \| \| Y \| \| \| \| 309 \| PTPRO \| 0.88 \| + \| 1.11E-01 \| 1 \| 4.09E-01 \| 1 \| -1.31 \| \| N \| \| \| \| 310 \| SELE \| 0.81 \| + \| 1.13E-01 \| 1 \| 7.87E-02 \| 1 \| 0.36 \| \| Y \| \| \| \| 311 \| BEND4 \| 0.87 \| - \| 1.15E-01 \| 1 \| 5.95E-02 \| 1 \| 0.66 \| \| N \| \| \| \| 312 \| CLEC17A \| 0.90 \| - \| 1.19E-01 \| 1 \| 7.36E-02 \| 1 \| 0.48 \| \| N \| \| \| \| 313 \| CNR2 \| 0.87 \| + \| 1.20E-01 \| 1 \| 2.06E-01 \| 1 \| -0.54 \| \| Y \| \| \| \| 314 \| CD5 \| 0.67 \| - \| 1.25E-01 \| 1 \| 1.01E-01 \| 1 \| 0.21 \| \| Y \| \| \| \| 315 \| SEZ6 \| 0.63 \| - \| 1.28E-01 \| 1 \| 5.43E-02 \| 1 \| 0.86 \| \| N \| \| \| \| 316 \| SNX20 \| 0.42 \| - \| 1.32E-01 \| 1 \| 2.28E-01 \| 1 \| -0.55 \| \| N \| \| \| \| 317 \| HLA-DMB \| 0.73 \| + \| 1.33E-01 \| 1 \| 8.73E-01 \| 1 \| -1.88 \| \| Y \| \| \| \| 318 \| RGS18 \| 0.63 \| + \| 1.33E-01 \| 1 \| 7.65E-01 \| 1 \| -1.75 \| \| N \| \| \| \| 319 \| TUBA3E \| 0.88 \| - \| 1.39E-01 \| 1 \| 1.36E-01 \| 1 \| 0.02 \| \| N \| \| \| \| 320 \| CHIT1 \| 0.87 \| + \| 1.41E-01 \| 1 \| 4.84E-01 \| 1 \| -1.23 \| \| Y \| \| \| \| 321 \| IRF8 \| 0.86 \| + \| 1.43E-01 \| 1 \| 5.78E-01 \| 1 \| -1.40 \| \| Y \| \| \| \| 322 \| UNC13A \| 0.81 \| - \| 1.52E-01 \| 1 \| 4.60E-02 \| 1 \| 1.19 \| \| N \| \| \| \| 323 \| FAM78A \| 0.72 \| - \| 1.53E-01 \| 1 \| 5.35E-01 \| 1 \| -1.26 \| \| N \| \| \| \| 324 \| BATF2 \| 0.91 \| - \| 1.55E-01 \| 1 \| 3.40E-01 \| 1 \| -0.78 \| \| N \| \| \| \| 325 \| SH2D1A \| 0.62 \| - \| 1.57E-01 \| 1 \| 2.82E-01 \| 1 \| -0.58 \| \| Y \| \| \| \| 326 \| PRSS3 \| 0.76 \| - \| 1.62E-01 \| 1 \| 1.19E-01 \| 1 \| 0.31 \| \| N \| \| \| \| 327 \| CXCL11 \| 0.68 \| - \| 1.64E-01 \| 1 \| 1.78E-01 \| 1 \| -0.08 \| \| Y \| \| \| \| 328 \| GUCA2B \| 0.88 \| + \| 1.67E-01 \| 1 \| 2.67E-01 \| 1 \| -0.47 \| \| N \| \| \| \| 329 \| CRHR2 \| 0.59 \| + \| 1.67E-01 \| 1 \| 6.18E-01 \| 1 \| -1.31 \| \| N \| \| \| \| 330 \| ITGB2 \| 0.62 \| - \| 1.68E-01 \| 1 \| 2.17E-01 \| 1 \| -0.26 \| \| Y \| \| \| \| 331 \| TKTL1 \| 0.80 \| + \| 1.69E-01 \| 1 \| 4.35E-01 \| 1 \| -0.95 \| \| N \| \| \| \| 332 \| SLAMF6 \| 0.62 \| - \| 1.70E-01 \| 1 \| 4.27E-01 \| 1 \| -0.92 \| \| Y \| \| \| \| 333 \| DKK1 \| 0.73 \| + \| 1.73E-01 \| 1 \| 5.67E-02 \| 1 \| 1.12 \| \| N \| \| \| \| 334 \| XIRP1 \| 0.86 \| - \| 1.75E-01 \| 1 \| 1.12E-01 \| 1 \| 0.45 \| \| N \| \| \| \| 335 \| FAM69C \| 0.71 \| - \| 1.78E-01 \| 1 \| 1.40E-01 \| 1 \| 0.24 \| \| N \| \| \| \| 336 \| FYB \| 0.55 \| - \| 1.78E-01 \| 1 \| 3.38E-01 \| 1 \| -0.64 \| \| Y \| \| \| \| 337 \| FCGR2A \| 0.62 \| - \| 1.79E-01 \| 1 \| 6.34E-02 \| 1 \| 1.04 \| \| Y \| \| \| \| 338 \| NELL2 \| 0.86 \| - \| 1.81E-01 \| 1 \| 1.50E-01 \| 1 \| 0.19 \| \| N \| \| \| \| 339 \| FCRLA \| 0.89 \| - \| 1.84E-01 \| 1 \| 4.68E-01 \| 1 \| -0.93 \| \| Y \| \| \| \| 340 \| MAPK8IP2 \| 0.90 \| - \| 1.85E-01 \| 1 \| 3.07E-02 \| 1 \| 1.80 \| \| N \| \| \| \| 341 \| LPAR5 \| 0.75 \| - \| 1.88E-01 \| 1 \| 4.27E-01 \| 1 \| -0.82 \| \| N \| \| \| \| 342 \| TM7SF4 \| 0.77 \| - \| 1.88E-01 \| 1 \| 5.34E-02 \| 1 \| 1.26 \| \| N \| \| \| \| 343 \| FAM129C \| 0.82 \| - \| 1.90E-01 \| 1 \| 5.40E-02 \| 1 \| 1.26 \| \| N \| \| \| \| 344 \| CD69 \| 0.76 \| - \| 1.94E-01 \| 1 \| 1.02E-01 \| 1 \| 0.65 \| \| Y \| \| \| \| 345 \| SIT1 \| 0.67 \| - \| 1.98E-01 \| 1 \| 1.76E-01 \| 1 \| 0.12 \| \| Y \| \| \| \| 346 \| C3AR1 \| 0.54 \| + \| 2.04E-01 \| 1 \| 8.95E-01 \| 1 \| -1.48 \| \| Y \| \| \| \| 347 \| MT1M \| 0.90 \| - \| 2.11E-01 \| 1 \| 1.86E-01 \| 1 \| 0.12 \| \| N \| \| \| \| 348 \| CHRNB2 \| 0.61 \| - \| 2.12E-01 \| 1 \| 8.48E-03 \| 1 \| 3.22 \| \| N \| \| \| \| 349 \| SIGLEC14 \| 0.58 \| - \| 2.12E-01 \| 1 \| 7.16E-01 \| 1 \| -1.22 \| \| Y \| \| \| \| 350 \| GATA1 \| 0.77 \| + \| 2.22E-01 \| 1 \| 3.34E-01 \| 1 \| -0.41 \| \| N \| \| \| \| 351 \| HEMGN \| 0.87 \| + \| 2.22E-01 \| 1 \| 7.47E-01 \| 1 \| -1.21 \| \| N \| \| \| \| 352 \| CD4 \| 0.67 \| - \| 2.25E-01 \| 1 \| 2.00E-01 \| 1 \| 0.12 \| \| Y \| \| \| \| 353 \| MAB21L1 \| 0.80 \| + \| 2.31E-01 \| 1 \| 2.93E-01 \| 1 \| -0.24 \| \| N \| \| \| \| 354 \| CACNA1B \| 0.79 \| - \| 2.35E-01 \| 1 \| 4.35E-01 \| 1 \| -0.62 \| \| N \| \| \| \| 355 \| MYPN \| 0.85 \| - \| 2.39E-01 \| 1 \| 8.81E-03 \| 1 \| 3.30 \| \| N \| \| \| \| 356 \| CD244 \| 0.65 \| - \| 2.43E-01 \| 1 \| 3.10E-01 \| 1 \| -0.24 \| \| Y \| \| \| \| 357 \| CCR5 \| 0.50 \| - \| 2.47E-01 \| 1 \| 8.68E-01 \| 1 \| -1.26 \| \| Y \| \| \| \| 358 \| GPR141 \| 0.55 \| + \| 2.48E-01 \| 1 \| 4.67E-01 \| 1 \| -0.63 \| \| N \| \| \| \| 359 \| TOX \| 0.76 \| - \| 2.56E-01 \| 1 \| 3.43E-01 \| 1 \| -0.29 \| \| N \| \| \| \| 360 \| LILRA3 \| 0.75 \| - \| 2.65E-01 \| 1 \| 4.77E-01 \| 1 \| -0.59 \| \| Y \| \| \| \| 361 \| SCML4 \| 0.82 \| - \| 2.68E-01 \| 1 \| 2.17E-01 \| 1 \| 0.21 \| \| N \| \| \| \| 362 \| IKZF1 \| 0.43 \| + \| 2.71E-01 \| 1 \| 9.29E-01 \| 1 \| -1.23 \| \| Y \| \| \| \| 363 \| LAX1 \| 0.69 \| - \| 2.73E-01 \| 1 \| 9.04E-01 \| 1 \| -1.20 \| \| Y \| \| \| \| 364 \| DNAJC5B \| 0.74 \| - \| 2.75E-01 \| 1 \| 3.05E-01 \| 1 \| -0.10 \| \| N \| \| \| \| 365 \| HLA-DMA \| 0.70 \| - \| 2.76E-01 \| 1 \| 2.47E-01 \| 1 \| 0.11 \| \| Y \| \| \| \| 366 \| EPO \| 0.89 \| - \| 2.79E-01 \| 1 \| 1.09E-01 \| 1 \| 0.94 \| \| Y \| \| \| \| 367 \| GPR15 \| 0.91 \| - \| 2.86E-01 \| 1 \| 1.43E-01 \| 1 \| 0.69 \| \| N \| \| \| \| 368 \| ITGAM \| 0.72 \| + \| 2.88E-01 \| 1 \| 3.07E-01 \| 1 \| -0.06 \| \| Y \| \| \| \| 369 \| PCSK1 \| 0.66 \| - \| 2.89E-01 \| 1 \| 1.50E-01 \| 1 \| 0.65 \| \| N \| \| \| \| 370 \| FAIM3 \| 0.80 \| + \| 2.90E-01 \| 1 \| 8.00E-01 \| 1 \| -1.02 \| \| Y \| \| \| \| 371 \| HCLS1 \| 0.69 \| - \| 2.94E-01 \| 1 \| 4.57E-01 \| 1 \| -0.44 \| \| N \| \| \| \| 372 \| CD180 \| 0.81 \| + \| 3.09E-01 \| 1 \| 3.97E-01 \| 1 \| -0.25 \| \| Y \| \| \| \| 373 \| NCKAP1L \| 0.37 \| - \| 3.12E-01 \| 1 \| 6.05E-01 \| 1 \| -0.66 \| \| Y \| \| \| \| 374 \| TSPO2 \| 0.87 \| - \| 3.14E-01 \| 1 \| 4.45E-01 \| 1 \| -0.35 \| \| N \| \| \| \| 375 \| PLEK \| 0.74 \| - \| 3.14E-01 \| 1 \| 2.98E-01 \| 1 \| 0.05 \| \| N \| \| \| \| 376 \| ZNF831 \| 0.62 \| + \| 3.17E-01 \| 1 \| 3.50E-01 \| 1 \| -0.10 \| \| N \| \| \| \| 377 \| NAPSB \| 0.80 \| + \| 3.23E-01 \| 1 \| 7.20E-01 \| 1 \| -0.80 \| \| N \| \| \| \| 378 \| TCL1A \| 0.86 \| + \| 3.24E-01 \| 1 \| 2.71E-01 \| 1 \| 0.18 \| \| Y \| \| \| \| 379 \| FIGF \| 0.70 \| - \| 3.30E-01 \| 1 \| 2.28E-01 \| 1 \| 0.37 \| \| N \| \| \| \| 380 \| CIITA \| 0.57 \| - \| 3.31E-01 \| 1 \| 6.13E-01 \| 1 \| -0.62 \| \| Y \| \| \| \| 381 \| ITGAD \| 0.87 \| + \| 3.37E-01 \| 1 \| 2.81E-01 \| 1 \| 0.18 \| \| Y \| \| \| \| 382 \| CRTAM \| 0.57 \| - \| 3.42E-01 \| 1 \| 4.49E-01 \| 1 \| -0.27 \| \| Y \| \| \| \| 383 \| SLCO2B1 \| 0.80 \| + \| 3.55E-01 \| 1 \| 3.96E-01 \| 1 \| -0.11 \| \| N \| \| \| \| 384 \| FCER2 \| 0.87 \| - \| 3.57E-01 \| 1 \| 5.87E-02 \| 1 \| 1.80 \| \| Y \| \| \| \| 385 \| KLHL6 \| 0.76 \| = \| 3.60E-01 \| 1 \| 3.04E-01 \| 1 \| 0.17 \| \| Y \| \| \| \| 386 \| ALAS2 \| 0.90 \| + \| 3.69E-01 \| 1 \| 9.19E-01 \| 1 \| -0.91 \| \| N \| \| \| \| 387 \| CLEC10A \| 0.89 \| + \| 3.70E-01 \| 1 \| 8.29E-01 \| 1 \| -0.81 \| \| Y \| \| \| \| 388 \| RNASE6 \| 0.65 \| + \| 3.70E-01 \| 1 \| 9.50E-01 \| 1 \| -0.94 \| \| N \| \| \| \| 389 \| SYT2 \| 0.91 \| - \| 3.71E-01 \| 1 \| 1.39E-02 \| 1 \| 3.29 \| \| N \| \| \| \| 390 \| CCL18 \| 0.83 \| - \| 3.75E-01 \| 1 \| 8.26E-01 \| 1 \| -0.79 \| \| Y \| \| \| \| 391 \| NLRC4 \| 0.53 \| + \| 3.78E-01 \| 1 \| 6.25E-01 \| 1 \| -0.50 \| \| Y \| \| \| \| 392 \| CD28 \| 0.87 \| - \| 3.87E-01 \| 1 \| 1.17E-01 \| 1 \| 1.19 \| \| Y \| \| \| \| 393 \| CD8B \| 0.71 \| - \| 3.88E-01 \| 1 \| 5.30E-01 \| 1 \| -0.31 \| \| Y \| \| \| \| 394 \| SYP \| 0.80 \| + \| 3.98E-01 \| 1 \| 8.91E-01 \| 1 \| -0.80 \| \| N \| \| \| \| 395 \| TMEM155 \| 0.86 \| - \| 4.03E-01 \| 1 \| 6.76E-01 \| 1 \| -0.52 \| \| N \| \| \| \| 396 \| GPR82 \| 0.73 \| + \| 4.18E-01 \| 1 \| 3.13E-01 \| 1 \| 0.29 \| \| N \| \| \| \| 397 \| CLLU1 \| 0.70 \| - \| 4.20E-01 \| 1 \| 3.46E-01 \| 1 \| 0.19 \| \| N \| \| \| \| 398 \| NLRP3 \| 0.80 \| + \| 4.28E-01 \| 1 \| 8.10E-01 \| 1 \| -0.64 \| \| Y \| \| \| \| 399 \| KRT2 \| 0.88 \| - \| 4.30E-01 \| 1 \| 5.35E-01 \| 1 \| -0.22 \| \| N \| \| \| \| 400 \| IFIT1B \| 0.88 \| + \| 4.30E-01 \| 1 \| 4.10E-01 \| 1 \| 0.05 \| \| N \| \| \| \| 401 \| SIGLEC5 \| 0.81 \| + \| 4.34E-01 \| 1 \| 3.10E-01 \| 1 \| 0.34 \| \| Y \| \| \| \| 402 \| MSR1 \| 0.58 \| + \| 4.34E-01 \| 1 \| 8.12E-01 \| 1 \| -0.63 \| \| Y \| \| \| \| 403 \| CD163 \| 0.59 \| + \| 4.58E-01 \| 1 \| 9.26E-01 \| 1 \| -0.70 \| \| Y \| \| \| \| 404 \| HLA-DQA2 \| 0.70 \| + \| 4.64E-01 \| 1 \| 7.14E-01 \| 1 \| -0.43 \| \| Y \| \| \| \| 405 \| CXCL9 \| 0.60 \| - \| 4.65E-01 \| 1 \| 8.70E-01 \| 1 \| -0.63 \| \| Y \| \| \| \| 406 \| C1QTNF9B \| 0.80 \| + \| 4.69E-01 \| 1 \| 8.22E-01 \| 1 \| -0.56 \| \| N \| \| \| \| 407 \| HBD \| 0.85 \| + \| 4.75E-01 \| 1 \| 7.80E-01 \| 1 \| -0.49 \| \| N \| \| \| \| 408 \| SIGLEC7 \| 0.54 \| + \| 4.78E-01 \| 1 \| 5.54E-01 \| 1 \| -0.15 \| \| Y \| \| \| \| 409 \| NRXN1 \| 0.63 \| - \| 4.78E-01 \| 1 \| 7.30E-01 \| 1 \| -0.42 \| \| N \| \| \| \| 410 \| TRAT1 \| 0.61 \| - \| 4.85E-01 \| 1 \| 3.71E-01 \| 1 \| 0.27 \| \| Y \| \| \| \| 411 \| POU3F1 \| 0.80 \| - \| 4.90E-01 \| 1 \| 8.39E-01 \| 1 \| -0.54 \| \| Y \| \| \| \| 412 \| NEFH \| 0.65 \| - \| 4.92E-01 \| 1 \| 1.40E-01 \| 1 \| 1.26 \| \| N \| \| \| \| 413 \| ARHGAP30 \| 0.51 \| - \| 5.06E-01 \| 1 \| 3.50E-01 \| 1 \| 0.37 \| \| N \| \| \| \| 414 \| LOC400759 \| 0.72 \| - \| 5.07E-01 \| 1 \| 6.02E-01 \| 1 \| -0.17 \| \| N \| \| \| \| 415 \| EVI2B \| 0.43 \| - \| 5.10E-01 \| 1 \| 8.57E-01 \| 1 \| -0.52 \| \| N \| \| \| \| 416 \| ADAM28 \| 0.89 \| + \| 5.21E-01 \| 1 \| 8.92E-01 \| 1 \| -0.54 \| \| N \| \| \| \| 417 \| CXCR2P1 \| 0.66 \| - \| 5.26E-01 \| 1 \| 8.84E-01 \| 1 \| -0.52 \| \| N \| \| \| \| 418 \| MS4A4A \| 0.62 \| + \| 5.29E-01 \| 1 \| 2.90E-01 \| 1 \| 0.60 \| \| Y \| \| \| \| 419 \| CD300LF \| 0.65 \| - \| 5.33E-01 \| 1 \| 5.11E-01 \| 1 \| 0.04 \| \| Y \| \| \| \| 420 \| SPN \| 0.54 \| + \| 5.37E-01 \| 1 \| 1.64E-01 \| 1 \| 1.19 \| \| Y \| \| \| \| 421 \| SLC15A3 \| 0.86 \| - \| 5.42E-01 \| 1 \| 6.76E-02 \| 1 \| 2.08 \| \| N \| \| \| \| 422 \| CD48 \| 0.59 \| - \| 5.47E-01 \| 1 \| 4.99E-01 \| 1 \| 0.09 \| \| Y \| \| \| \| 423 \| WNT9A \| 0.89 \| + \| 5.49E-01 \| 1 \| 3.64E-01 \| 1 \| 0.41 \| \| N \| \| \| \| 424 \| BTLA \| 0.80 \| - \| 5.49E-01 \| 1 \| 7.08E-01 \| 1 \| -0.25 \| \| Y \| \| \| \| 425 \| CLECL1 \| 0.75 \| - \| 5.56E-01 \| 1 \| 3.39E-01 \| 1 \| 0.49 \| \| Y \| \| \| \| 426 \| MGC4473 \| 0.89 \| - \| 5.65E-01 \| 1 \| 6.40E-01 \| 1 \| -0.13 \| \| N \| \| \| \| 427 \| AMICA1 \| 0.82 \| - \| 5.70E-01 \| 1 \| 1.15E-01 \| 1 \| 1.60 \| \| N \| \| \| \| 428 \| FCGBP \| 0.87 \| - \| 5.72E-01 \| 1 \| 8.89E-01 \| 1 \| -0.44 \| \| Y \| \| \| \| 429 \| CD209 \| 0.79 \| + \| 5.76E-01 \| 1 \| 2.26E-01 \| 1 \| 0.93 \| \| Y \| \| \| \| 430 \| HLA-DRB5 \| 0.79 \| - \| 5.80E-01 \| 1 \| 5.15E-01 \| 1 \| 0.12 \| \| Y \| \| \| \| 431 \| GAPT \| 0.70 \| + \| 5.82E-01 \| 1 \| 6.81E-01 \| 1 \| -0.16 \| \| N \| \| \| \| 432 \| FLJ45983 \| 0.82 \| - \| 5.86E-01 \| 1 \| 1.78E-01 \| 1 \| 1.19 \| \| N \| \| \| \| 433 \| HLA-DRB1 \| 0.69 \| - \| 5.91E-01 \| 1 \| 9.96E-01 \| 1 \| -0.52 \| \| N \| \| \| \| 434 \| SPTA1 \| 0.85 \| + \| 5.91E-01 \| 1 \| 4.21E-01 \| 1 \| 0.34 \| \| N \| \| \| \| 435 \| HLA-DQB1 \| 0.73 \| + \| 6.12E-01 \| 1 \| 3.91E-01 \| 1 \| 0.45 \| \| Y \| \| \| \| 436 \| CLEC4A \| 0.61 \| - \| 6.13E-01 \| 1 \| 9.72E-01 \| 1 \| -0.46 \| \| Y \| \| \| \| 437 \| EOMES \| 0.58 \| - \| 6.16E-01 \| 1 \| 8.39E-01 \| 1 \| -0.31 \| \| Y \| \| \| \| 438 \| LDLRAD2 \| 0.88 \| + \| 6.38E-01 \| 1 \| 3.17E-01 \| 1 \| 0.70 \| \| N \| \| \| \| 439 \| FPR1 \| 0.71 \| + \| 6.41E-01 \| 1 \| 5.56E-01 \| 1 \| 0.14 \| \| Y \| \| \| \| 440 \| LCP1 \| 0.38 \| + \| 6.44E-01 \| 1 \| 7.22E-01 \| 1 \| -0.11 \| \| Y \| \| \| \| 441 \| CLEC12A \| 0.62 \| - \| 6.45E-01 \| 1 \| 4.72E-01 \| 1 \| 0.31 \| \| Y \| \| \| \| 442 \| HLA-DPB2 \| 0.55 \| - \| 6.47E-01 \| 1 \| 8.48E-01 \| 1 \| -0.27 \| \| N \| \| \| \| 443 \| MS4A6A \| 0.41 \| + \| 6.49E-01 \| 1 \| 4.10E-01 \| 1 \| 0.46 \| \| Y \| \| \| \| 444 \| ABCD2 \| 0.77 \| - \| 6.58E-01 \| 1 \| 9.40E-01 \| 1 \| -0.36 \| \| N \| \| \| \| 445 \| CA3 \| 0.53 \| - \| 6.60E-01 \| 1 \| 1.91E-01 \| 1 \| 1.24 \| \| N \| \| \| \| 446 \| DPEP3 \| 0.84 \| + \| 6.66E-01 \| 1 \| 7.08E-01 \| 1 \| -0.06 \| \| N \| \| \| \| 447 \| ITK \| 0.64 \| - \| 6.70E-01 \| 1 \| 5.51E-01 \| 1 \| 0.20 \| \| Y \| \| \| \| 448 \| NKAIN2 \| 0.69 \| - \| 6.78E-01 \| 1 \| 3.26E-01 \| 1 \| 0.73 \| \| N \| \| \| \| 449 \| IL16 \| 0.69 \| - \| 6.83E-01 \| 1 \| 5.22E-01 \| 1 \| 0.27 \| \| Y \| \| \| \| 450 \| P2RY10 \| 0.57 \| - \| 6.87E-01 \| 1 \| 5.67E-01 \| 1 \| 0.19 \| \| N \| \| \| \| 451 \| CD200R1 \| 0.57 \| + \| 6.87E-01 \| 1 \| 9.97E-01 \| 1 \| -0.37 \| \| Y \| \| \| \| 452 \| UBD \| 0.83 \| - \| 6.88E-01 \| 1 \| 4.48E-01 \| 1 \| 0.43 \| \| Y \| \| \| \| 453 \| HLA-DRB6 \| 0.68 \| + \| 6.90E-01 \| 1 \| 9.45E-01 \| 1 \| -0.32 \| \| N \| \| \| \| 454 \| SOX2OT \| 0.85 \| + \| 6.96E-01 \| 1 \| 5.44E-01 \| 1 \| 0.25 \| \| N \| \| \| \| 455 \| THEMIS \| 0.61 \| - \| 6.99E-01 \| 1 \| 9.03E-01 \| 1 \| -0.26 \| \| Y \| \| \| \| 456 \| KCND3 \| 0.88 \| + \| 7.04E-01 \| 1 \| 9.82E-01 \| 1 \| -0.33 \| \| N \| \| \| \| 457 \| SLC10A4 \| 0.89 \| = \| 7.09E-01 \| 1 \| 6.35E-01 \| 1 \| 0.11 \| \| N \| \| \| \| 458 \| CRYBB3 \| 0.90 \| + \| 7.17E-01 \| 1 \| 9.77E-01 \| 1 \| -0.31 \| \| N \| \| \| \| 459 \| KCNC1 \| 0.88 \| + \| 7.22E-01 \| 1 \| 2.14E-01 \| 1 \| 1.22 \| \| N \| \| \| \| 460 \| DOCK2 \| 0.55 \| + \| 7.28E-01 \| 1 \| 9.61E-01 \| 1 \| -0.28 \| \| Y \| \| \| \| 461 \| TLR10 \| 0.73 \| - \| 7.30E-01 \| 1 \| 3.78E-01 \| 1 \| 0.66 \| \| Y \| \| \| \| 462 \| GPR55 \| 0.77 \| + \| 7.45E-01 \| 1 \| 8.74E-01 \| 1 \| -0.16 \| \| N \| \| \| \| 463 \| TIFAB \| 0.75 \| - \| 7.46E-01 \| 1 \| 7.52E-01 \| 1 \| -0.01 \| \| N \| \| \| \| 464 \| ADORA3 \| 0.67 \| + \| 7.57E-01 \| 1 \| 7.33E-01 \| 1 \| 0.03 \| \| Y \| \| \| \| 465 \| DAB1 \| 0.77 \| + \| 7.58E-01 \| 1 \| 9.16E-01 \| 1 \| -0.19 \| \| N \| \| \| \| 466 \| BTK \| 0.55 \| - \| 7.64E-01 \| 1 \| 9.28E-01 \| 1 \| -0.19 \| \| Y \| \| \| \| 467 \| FCRL3 \| 0.81 \| - \| 7.65E-01 \| 1 \| 7.52E-01 \| 1 \| 0.02 \| \| N \| \| \| \| 468 \| CCR2 \| 0.61 \| - \| 7.66E-01 \| 1 \| 5.98E-01 \| 1 \| 0.25 \| \| Y \| \| \| \| 469 \| LY9 \| 0.70 \| - \| 7.68E-01 \| 1 \| 2.75E-01 \| 1 \| 1.03 \| \| Y \| \| \| \| 470 \| IL5RA \| 0.80 \| + \| 7.70E-01 \| 1 \| 3.44E-01 \| 1 \| 0.81 \| \| Y \| \| \| \| 471 \| PTPRC \| 0.48 \| - \| 7.78E-01 \| 1 \| 7.57E-01 \| 1 \| 0.03 \| \| Y \| \| \| \| 472 \| C8orf84 \| 0.87 \| + \| 7.82E-01 \| 1 \| 9.67E-01 \| 1 \| -0.21 \| \| Y \| \| \| \| 473 \| GPR174 \| 0.61 \| - \| 7.83E-01 \| 1 \| 8.91E-01 \| 1 \| -0.13 \| \| N \| \| \| \| 474 \| CLEC6A \| 0.77 \| - \| 7.85E-01 \| 1 \| 9.05E-01 \| 1 \| -0.14 \| \| Y \| \| \| \| 475 \| FAM19A2 \| 0.57 \| - \| 7.92E-01 \| 1 \| 6.23E-01 \| 1 \| 0.24 \| \| N \| \| \| \| 476 \| FRG2C \| 0.72 \| + \| 7.96E-01 \| 1 \| 9.94E-01 \| 1 \| -0.22 \| \| N \| \| \| \| 477 \| FOLR2 \| 0.84 \| + \| 8.03E-01 \| 1 \| 4.90E-01 \| 1 \| 0.49 \| \| N \| \| \| \| 478 \| DPEP2 \| 0.88 \| + \| 8.12E-01 \| 1 \| 7.28E-01 \| 1 \| 0.11 \| \| N \| \| \| \| 479 \| GFI1B \| 0.81 \| + \| 8.42E-01 \| 1 \| 5.58E-01 \| 1 \| 0.41 \| \| N \| \| \| \| 480 \| CASP1 \| 0.83 \| + \| 8.46E-01 \| 1 \| 4.69E-01 \| 1 \| 0.59 \| \| Y \| \| \| \| 481 \| DCC \| 0.72 \| - \| 8.51E-01 \| 1 \| 7.28E-01 \| 1 \| 0.16 \| \| N \| \| \| \| 482 \| C17orf87 \| 0.51 \| + \| 8.55E-01 \| 1 \| 7.64E-01 \| 1 \| 0.11 \| \| N \| \| \| \| 483 \| EPX \| 0.75 \| + \| 8.68E-01 \| 1 \| 5.86E-01 \| 1 \| 0.39 \| \| Y \| \| \| \| 484 \| CLSTN2 \| 0.90 \| + \| 8.74E-01 \| 1 \| 4.94E-01 \| 1 \| 0.57 \| \| N \| \| \| \| 485 \| MS4A1 \| 0.84 \| - \| 8.75E-01 \| 1 \| 5.52E-01 \| 1 \| 0.46 \| \| Y \| \| \| \| 486 \| SLC16A6 \| 0.83 \| + \| 8.92E-01 \| 1 \| 8.19E-01 \| 1 \| 0.09 \| \| N \| \| \| \| 487 \| KLRB1 \| 0.83 \| - \| 8.98E-01 \| 1 \| 1.81E-01 \| 1 \| 1.60 \| \| Y \| \| \| \| 488 \| CSF2RB \| 0.84 \| + \| 9.02E-01 \| 1 \| 8.36E-01 \| 1 \| 0.08 \| \| Y \| \| \| \| 489 \| TBXAS1 \| 0.87 \| = \| 9.04E-01 \| 1 \| 5.31E-01 \| 1 \| 0.53 \| \| Y \| \| \| \| 490 \| LY86 \| 0.67 \| - \| 9.05E-01 \| 1 \| 3.14E-01 \| 1 \| 1.06 \| \| Y \| \| \| \| 491 \| DTHD1 \| 0.73 \| - \| 9.19E-01 \| 1 \| 4.42E-01 \| 1 \| 0.73 \| \| N \| \| \| \| 492 \| NCF1C \| 0.88 \| - \| 9.20E-01 \| 1 \| 7.50E-01 \| 1 \| 0.20 \| \| Y \| \| \| \| 493 \| SELL \| 0.83 \| - \| 9.41E-01 \| 1 \| 2.77E-01 \| 1 \| 1.22 \| \| Y \| \| \| \| 494 \| C3orf45 \| 0.83 \| + \| 9.47E-01 \| 1 \| 2.72E-01 \| 1 \| 1.25 \| \| N \| \| \| \| 495 \| GRAP2 \| 0.73 \| - \| 9.56E-01 \| 1 \| 9.05E-01 \| 1 \| 0.05 \| \| Y \| \| \| \| 496 \| CXorf21 \| 0.74 \| - \| 9.67E-01 \| 1 \| 7.81E-01 \| 1 \| 0.21 \| \| N \| \| \| \| 497 \| MMP16 \| 0.86 \| + \| 9.76E-01 \| 1 \| 9.74E-01 \| 1 \| 0.00 \| \| N \| \| \| \| 498 \| C5orf20 \| 0.89 \| + \| 9.83E-01 \| 1 \| 8.44E-01 \| 1 \| 0.15 \| \| Y \| \| \| \| 499 \| AQP7P1 \| 0.89 \| + \| 9.83E-01 \| 1 \| 7.60E-01 \| 1 \| 0.26 \| \| N \| \| \| \| 500 \| CD40LG \| 0.84 \| + \| 1 \| 1 \| 3.03E-01 \| 1 \| 1.19 \| \| Y \| \| \| | | | | | | | |  |  |
| --- | --- | --- | --- | --- | --- | --- | --- | --- | --- | --- | --- | --- | --- | --- | --- | --- | --- | --- | --- | --- | --- | --- | --- | --- | --- | --- | --- | --- | --- | --- | --- | --- | --- | --- | --- | --- | --- | --- | --- | --- | --- | --- | --- | --- | --- | --- | --- | --- | --- | --- | --- | --- | --- | --- | --- | --- | --- | --- | --- | --- | --- | --- | --- | --- | --- | --- | --- | --- | --- | --- | --- | --- | --- | --- | --- | --- | --- | --- | --- | --- | --- | --- | --- | --- | --- | --- | --- | --- | --- | --- | --- | --- | --- | --- | --- | --- | --- | --- | --- | --- | --- | --- | --- | --- | --- | --- | --- | --- | --- | --- | --- | --- | --- | --- | --- | --- | --- | --- | --- | --- | --- | --- | --- | --- | --- | --- | --- | --- | --- | --- | --- | --- | --- | --- | --- | --- | --- | --- | --- | --- | --- | --- | --- | --- | --- | --- | --- | --- | --- | --- | --- | --- | --- | --- | --- | --- | --- | --- | --- | --- | --- | --- | --- | --- | --- | --- | --- | --- | --- | --- | --- | --- | --- | --- | --- | --- | --- | --- | --- | --- | --- | --- | --- | --- | --- | --- | --- | --- | --- | --- | --- | --- | --- | --- | --- | --- | --- | --- | --- | --- | --- | --- | --- | --- | --- | --- | --- | --- | --- | --- | --- | --- | --- | --- | --- | --- | --- | --- | --- | --- | --- | --- | --- | --- | --- | --- | --- | --- | --- | --- | --- | --- | --- | --- | --- | --- | --- | --- | --- | --- | --- | --- | --- | --- | --- | --- | --- | --- | --- | --- | --- | --- | --- | --- | --- | --- | --- | --- | --- | --- | --- | --- | --- | --- | --- | --- | --- | --- | --- | --- | --- | --- | --- | --- | --- | --- | --- | --- | --- | --- | --- | --- | --- | --- | --- | --- | --- | --- | --- | --- | --- | --- | --- | --- | --- | --- | --- | --- | --- | --- | --- | --- | --- | --- | --- | --- | --- | --- | --- | --- | --- | --- | --- | --- | --- | --- | --- | --- | --- | --- | --- | --- | --- | --- | --- | --- | --- | --- | --- | --- | --- | --- | --- | --- | --- | --- | --- | --- | --- | --- | --- | --- | --- | --- | --- | --- | --- | --- | --- | --- | --- | --- | --- | --- | --- | --- | --- | --- | --- | --- | --- | --- | --- | --- | --- | --- | --- | --- | --- | --- | --- | --- | --- | --- | --- | --- | --- | --- | --- | --- | --- | --- | --- | --- | --- | --- | --- | --- | --- | --- | --- | --- | --- | --- | --- | --- | --- | --- | --- | --- | --- | --- | --- | --- | --- | --- | --- | --- | --- | --- | --- | --- | --- | --- | --- | --- | --- | --- | --- | --- | --- | --- | --- | --- | --- | --- | --- | --- | --- | --- | --- | --- | --- | --- | --- | --- | --- | --- | --- | --- | --- | --- | --- | --- | --- | --- | --- | --- | --- | --- | --- | --- | --- | --- | --- | --- | --- | --- | --- | --- | --- | --- | --- | --- | --- | --- | --- | --- | --- | --- | --- | --- | --- | --- | --- | --- | --- | --- | --- | --- | --- | --- | --- | --- | --- | --- | --- | --- | --- | --- | --- | --- | --- | --- | --- | --- | --- | --- | --- | --- | --- | --- | --- | --- | --- | --- | --- | --- | --- | --- | --- | --- | --- | --- | --- | --- | --- | --- | --- | --- | --- | --- | --- | --- | --- | --- | --- | --- | --- | --- | --- | --- | --- | --- | --- | --- | --- | --- | --- | --- | --- | --- | --- | --- | --- | --- | --- | --- | --- | --- | --- | --- | --- | --- | --- | --- | --- | --- | --- | --- | --- | --- | --- | --- | --- | --- | --- | --- | --- | --- | --- | --- | --- | --- | --- | --- | --- | --- | --- | --- | --- | --- | --- | --- | --- | --- | --- | --- | --- | --- | --- | --- | --- | --- | --- | --- | --- | --- | --- | --- | --- | --- | --- | --- | --- | --- | --- | --- | --- | --- | --- | --- | --- | --- | --- | --- | --- | --- | --- | --- | --- | --- | --- | --- | --- | --- | --- | --- | --- | --- | --- | --- | --- | --- | --- | --- | --- | --- | --- | --- | --- | --- | --- | --- | --- | --- | --- | --- | --- | --- | --- | --- | --- | --- | --- | --- | --- | --- | --- | --- | --- | --- | --- | --- | --- | --- | --- | --- | --- | --- | --- | --- | --- | --- | --- | --- | --- | --- | --- | --- | --- | --- | --- | --- | --- | --- | --- | --- | --- | --- | --- | --- | --- | --- | --- | --- | --- | --- | --- | --- | --- | --- | --- | --- | --- | --- | --- | --- | --- | --- | --- | --- | --- | --- | --- | --- | --- | --- | --- | --- | --- | --- | --- | --- | --- | --- | --- | --- | --- | --- | --- | --- | --- | --- | --- | --- | --- | --- | --- | --- | --- | --- | --- | --- | --- | --- | --- | --- | --- | --- | --- | --- | --- | --- | --- | --- | --- | --- | --- | --- | --- | --- | --- | --- | --- | --- | --- | --- | --- | --- | --- | --- | --- | --- | --- | --- | --- | --- | --- | --- | --- | --- | --- | --- | --- | --- | --- | --- | --- | --- | --- | --- | --- | --- | --- | --- | --- | --- | --- | --- | --- | --- | --- | --- | --- | --- | --- | --- | --- | --- | --- | --- | --- | --- | --- | --- | --- | --- | --- | --- | --- | --- | --- | --- | --- | --- | --- | --- | --- | --- | --- | --- | --- | --- | --- | --- | --- | --- | --- | --- | --- | --- | --- | --- | --- | --- | --- | --- | --- | --- | --- | --- | --- | --- | --- | --- | --- | --- | --- | --- | --- | --- | --- | --- | --- | --- | --- | --- | --- | --- | --- | --- | --- | --- | --- | --- | --- | --- | --- | --- | --- | --- | --- | --- | --- | --- | --- | --- | --- | --- | --- | --- | --- | --- | --- | --- | --- | --- | --- | --- | --- | --- | --- | --- | --- | --- | --- | --- | --- | --- | --- | --- | --- | --- | --- | --- | --- | --- | --- | --- | --- | --- | --- | --- | --- | --- | --- | --- | --- | --- | --- | --- | --- | --- | --- | --- | --- | --- | --- | --- | --- | --- | --- | --- | --- | --- | --- | --- | --- | --- | --- | --- | --- | --- | --- | --- | --- | --- | --- | --- | --- | --- | --- | --- | --- | --- | --- | --- | --- | --- | --- | --- | --- | --- | --- | --- | --- | --- | --- | --- | --- | --- | --- | --- | --- | --- | --- | --- | --- | --- | --- | --- | --- | --- | --- | --- | --- | --- | --- | --- | --- | --- | --- | --- | --- | --- | --- | --- | --- | --- | --- | --- | --- | --- | --- | --- | --- | --- | --- | --- | --- | --- | --- | --- | --- | --- | --- | --- | --- | --- | --- | --- | --- | --- | --- | --- | --- | --- | --- | --- | --- | --- | --- | --- | --- | --- | --- | --- | --- | --- | --- | --- | --- | --- | --- | --- | --- | --- | --- | --- | --- | --- | --- | --- | --- | --- | --- | --- | --- | --- | --- | --- | --- | --- | --- | --- | --- | --- | --- | --- | --- | --- | --- | --- | --- | --- | --- | --- | --- | --- | --- | --- | --- | --- | --- | --- | --- | --- | --- | --- | --- | --- | --- | --- | --- | --- | --- | --- | --- | --- | --- | --- | --- | --- | --- | --- | --- | --- | --- | --- | --- | --- | --- | --- | --- | --- | --- | --- | --- | --- | --- | --- | --- | --- | --- | --- | --- | --- | --- | --- | --- | --- | --- | --- | --- | --- | --- | --- | --- | --- | --- | --- | --- | --- | --- | --- | --- | --- | --- | --- | --- | --- | --- | --- | --- | --- | --- | --- | --- | --- | --- | --- | --- | --- | --- | --- | --- | --- | --- | --- | --- | --- | --- | --- | --- | --- | --- | --- | --- | --- | --- | --- | --- | --- | --- | --- | --- | --- | --- | --- | --- | --- | --- | --- | --- | --- | --- | --- | --- | --- | --- | --- | --- | --- | --- | --- | --- | --- | --- | --- | --- | --- | --- | --- | --- | --- | --- | --- | --- | --- | --- | --- | --- | --- | --- | --- | --- | --- | --- | --- | --- | --- | --- | --- | --- | --- | --- | --- | --- | --- | --- | --- | --- | --- | --- | --- | --- | --- | --- | --- | --- | --- | --- | --- | --- | --- | --- | --- | --- | --- | --- | --- | --- | --- | --- | --- | --- | --- | --- | --- | --- | --- | --- | --- | --- | --- | --- | --- | --- | --- | --- | --- | --- | --- | --- | --- | --- | --- | --- | --- | --- | --- | --- | --- | --- | --- | --- | --- | --- | --- | --- | --- | --- | --- | --- | --- | --- | --- | --- | --- | --- | --- | --- | --- | --- | --- | --- | --- | --- | --- | --- | --- | --- | --- | --- | --- | --- | --- | --- | --- | --- | --- | --- | --- | --- | --- | --- | --- | --- | --- | --- | --- | --- | --- | --- | --- | --- | --- | --- | --- | --- | --- | --- | --- | --- | --- | --- | --- | --- | --- | --- | --- | --- | --- | --- | --- | --- | --- | --- | --- | --- | --- | --- | --- | --- | --- | --- | --- | --- | --- | --- | --- | --- | --- | --- | --- | --- | --- | --- | --- | --- | --- | --- | --- | --- | --- | --- | --- | --- | --- | --- | --- | --- | --- | --- | --- | --- | --- | --- | --- | --- | --- | --- | --- | --- | --- | --- | --- | --- | --- | --- | --- | --- | --- | --- | --- | --- | --- | --- | --- | --- | --- | --- | --- | --- | --- | --- | --- | --- | --- | --- | --- | --- | --- | --- | --- | --- | --- | --- | --- | --- | --- | --- | --- | --- | --- | --- | --- | --- | --- | --- | --- | --- | --- | --- | --- | --- | --- | --- | --- | --- | --- | --- | --- | --- | --- | --- | --- | --- | --- | --- | --- | --- | --- | --- | --- | --- | --- | --- | --- | --- | --- | --- | --- | --- | --- | --- | --- | --- | --- | --- | --- | --- | --- | --- | --- | --- | --- | --- | --- | --- | --- | --- | --- | --- | --- | --- | --- | --- | --- | --- | --- | --- | --- | --- | --- | --- | --- | --- | --- | --- | --- | --- | --- | --- | --- | --- | --- | --- | --- | --- | --- | --- | --- | --- | --- | --- | --- | --- | --- | --- | --- | --- | --- | --- | --- | --- | --- | --- | --- | --- | --- | --- | --- | --- | --- | --- | --- | --- | --- | --- | --- | --- | --- | --- | --- | --- | --- | --- | --- | --- | --- | --- | --- | --- | --- | --- | --- | --- | --- | --- | --- | --- | --- | --- | --- | --- | --- | --- | --- | --- | --- | --- | --- | --- | --- | --- | --- | --- | --- | --- | --- | --- | --- | --- | --- | --- | --- | --- | --- | --- | --- | --- | --- | --- | --- | --- | --- | --- | --- | --- | --- | --- | --- | --- | --- | --- | --- | --- | --- | --- | --- | --- | --- | --- | --- | --- | --- | --- | --- | --- | --- | --- | --- | --- | --- | --- | --- | --- | --- | --- | --- | --- | --- | --- | --- | --- | --- | --- | --- | --- | --- | --- | --- | --- | --- | --- | --- | --- | --- | --- | --- | --- | --- | --- | --- | --- | --- | --- | --- | --- | --- | --- | --- | --- | --- | --- | --- | --- | --- | --- | --- | --- | --- | --- | --- | --- | --- | --- | --- | --- | --- | --- | --- | --- | --- | --- | --- | --- | --- | --- | --- | --- | --- | --- | --- | --- | --- | --- | --- | --- | --- | --- | --- | --- | --- | --- | --- | --- | --- | --- | --- | --- | --- | --- | --- | --- | --- | --- | --- | --- | --- | --- | --- | --- | --- | --- | --- | --- | --- | --- | --- | --- | --- | --- | --- | --- | --- | --- | --- | --- | --- | --- | --- | --- | --- | --- | --- | --- | --- | --- | --- | --- | --- | --- | --- | --- | --- | --- | --- | --- | --- | --- | --- | --- | --- | --- | --- | --- | --- | --- | --- | --- | --- | --- | --- | --- | --- | --- | --- | --- | --- | --- | --- | --- | --- | --- | --- | --- | --- | --- | --- | --- | --- | --- | --- | --- | --- | --- | --- | --- | --- | --- | --- | --- | --- | --- | --- | --- | --- | --- | --- | --- | --- | --- | --- | --- | --- | --- | --- | --- | --- | --- | --- | --- | --- | --- | --- | --- | --- | --- | --- | --- | --- | --- | --- | --- | --- | --- | --- | --- | --- | --- | --- | --- | --- | --- | --- | --- | --- | --- | --- | --- | --- | --- | --- | --- | --- | --- | --- | --- | --- | --- | --- | --- | --- | --- | --- | --- | --- | --- | --- | --- | --- | --- | --- | --- | --- | --- | --- | --- | --- | --- | --- | --- | --- | --- | --- | --- | --- | --- | --- | --- | --- | --- | --- | --- | --- | --- | --- | --- | --- | --- | --- | --- | --- | --- | --- | --- | --- | --- | --- | --- | --- | --- | --- | --- | --- | --- | --- | --- | --- | --- | --- | --- | --- | --- | --- | --- | --- | --- | --- | --- | --- | --- | --- | --- | --- | --- | --- | --- | --- | --- | --- | --- | --- | --- | --- | --- | --- | --- | --- | --- | --- | --- | --- | --- | --- | --- | --- | --- | --- | --- | --- | --- | --- | --- | --- | --- | --- | --- | --- | --- | --- | --- | --- | --- | --- | --- | --- | --- | --- | --- | --- | --- | --- | --- | --- | --- | --- | --- | --- | --- | --- | --- | --- | --- | --- | --- | --- | --- | --- | --- | --- | --- | --- | --- | --- | --- | --- | --- | --- | --- | --- | --- | --- | --- | --- | --- | --- | --- | --- | --- | --- | --- | --- | --- | --- | --- | --- | --- | --- | --- | --- | --- | --- | --- | --- | --- | --- | --- | --- | --- | --- | --- | --- | --- | --- | --- | --- | --- | --- | --- | --- | --- | --- | --- | --- | --- | --- | --- | --- | --- | --- | --- | --- | --- | --- | --- | --- | --- | --- | --- | --- | --- | --- | --- | --- | --- | --- | --- | --- | --- | --- | --- | --- | --- | --- | --- | --- | --- | --- | --- | --- | --- | --- | --- | --- | --- | --- | --- | --- | --- | --- | --- | --- | --- | --- | --- | --- | --- | --- | --- | --- | --- | --- | --- | --- | --- | --- | --- | --- | --- | --- | --- | --- | --- | --- | --- | --- | --- | --- | --- | --- | --- | --- | --- | --- | --- | --- | --- | --- | --- | --- | --- | --- | --- | --- | --- | --- | --- | --- | --- | --- | --- | --- | --- | --- | --- | --- | --- | --- | --- | --- | --- | --- | --- | --- | --- | --- | --- | --- | --- | --- | --- | --- | --- | --- | --- | --- | --- | --- | --- | --- | --- | --- | --- | --- | --- | --- | --- | --- | --- | --- | --- | --- | --- | --- | --- | --- | --- | --- | --- | --- | --- | --- | --- | --- | --- | --- | --- | --- | --- | --- | --- | --- | --- | --- | --- | --- | --- | --- | --- | --- | --- | --- | --- | --- | --- | --- | --- | --- | --- | --- | --- | --- | --- | --- | --- | --- | --- | --- | --- | --- | --- | --- | --- | --- | --- | --- | --- | --- | --- | --- | --- | --- | --- | --- | --- | --- | --- | --- | --- | --- | --- | --- | --- | --- | --- | --- | --- | --- | --- | --- | --- | --- | --- | --- | --- | --- | --- | --- | --- | --- | --- | --- | --- | --- | --- | --- | --- | --- | --- | --- | --- | --- | --- | --- | --- | --- | --- | --- | --- | --- | --- | --- | --- | --- | --- | --- | --- | --- | --- | --- | --- | --- | --- | --- | --- | --- | --- | --- | --- | --- | --- | --- | --- | --- | --- | --- | --- | --- | --- | --- | --- | --- | --- | --- | --- | --- | --- | --- | --- | --- | --- | --- | --- | --- | --- | --- | --- | --- | --- | --- | --- | --- | --- | --- | --- | --- | --- | --- | --- | --- | --- | --- | --- | --- | --- | --- | --- | --- | --- | --- | --- | --- | --- | --- | --- | --- | --- | --- | --- | --- | --- | --- | --- | --- | --- | --- | --- | --- | --- | --- | --- | --- | --- | --- | --- | --- | --- | --- | --- | --- | --- | --- | --- | --- | --- | --- | --- | --- | --- | --- | --- | --- | --- | --- | --- | --- | --- | --- | --- | --- | --- | --- | --- | --- | --- | --- | --- | --- | --- | --- | --- | --- | --- | --- | --- | --- | --- | --- | --- | --- | --- | --- | --- | --- | --- | --- | --- | --- | --- | --- | --- | --- | --- | --- | --- | --- | --- | --- | --- | --- | --- | --- | --- | --- | --- | --- | --- | --- | --- | --- | --- | --- | --- | --- | --- | --- | --- | --- | --- | --- | --- | --- | --- | --- | --- | --- | --- | --- | --- | --- | --- | --- | --- | --- | --- | --- | --- | --- | --- | --- | --- | --- | --- | --- | --- | --- | --- | --- | --- | --- | --- | --- | --- | --- | --- | --- | --- | --- | --- | --- | --- | --- | --- | --- | --- | --- | --- | --- | --- | --- | --- | --- | --- | --- | --- | --- | --- | --- | --- | --- | --- | --- | --- | --- | --- | --- | --- | --- | --- | --- | --- | --- | --- | --- | --- | --- | --- | --- | --- | --- | --- | --- | --- | --- | --- | --- | --- | --- | --- | --- | --- | --- | --- | --- | --- | --- | --- | --- | --- | --- | --- | --- | --- | --- | --- | --- | --- | --- | --- | --- | --- | --- | --- | --- | --- | --- | --- | --- | --- | --- | --- | --- | --- | --- | --- | --- | --- | --- | --- | --- | --- | --- | --- | --- | --- | --- | --- | --- | --- | --- | --- | --- | --- | --- | --- | --- | --- | --- | --- | --- | --- | --- | --- | --- | --- | --- | --- | --- | --- | --- | --- | --- | --- | --- | --- | --- | --- | --- | --- | --- | --- | --- | --- | --- | --- | --- | --- | --- | --- | --- | --- | --- | --- | --- | --- | --- | --- | --- | --- | --- | --- | --- | --- | --- | --- | --- | --- | --- | --- | --- | --- | --- | --- | --- | --- | --- | --- | --- | --- | --- | --- | --- | --- | --- | --- | --- | --- | --- | --- | --- | --- | --- | --- | --- | --- | --- | --- | --- | --- | --- | --- | --- | --- | --- | --- | --- | --- | --- | --- | --- | --- | --- | --- | --- | --- | --- | --- | --- | --- | --- | --- | --- | --- | --- | --- | --- | --- | --- | --- | --- | --- | --- | --- | --- | --- | --- | --- | --- | --- | --- | --- | --- | --- | --- | --- | --- | --- | --- | --- | --- | --- | --- | --- | --- | --- | --- | --- | --- | --- | --- | --- | --- | --- | --- | --- | --- | --- | --- | --- | --- | --- | --- | --- | --- | --- | --- | --- | --- | --- | --- | --- | --- | --- | --- | --- | --- | --- | --- | --- | --- | --- | --- | --- | --- | --- | --- | --- | --- | --- | --- | --- | --- | --- | --- | --- | --- | --- | --- | --- | --- | --- | --- | --- | --- | --- | --- | --- | --- | --- | --- | --- | --- | --- | --- | --- | --- | --- | --- | --- | --- | --- | --- | --- | --- | --- | --- | --- | --- | --- | --- | --- | --- | --- | --- | --- | --- | --- | --- | --- | --- | --- | --- | --- | --- | --- | --- | --- | --- | --- | --- | --- | --- | --- | --- | --- | --- | --- | --- | --- | --- | --- | --- | --- | --- | --- | --- | --- | --- | --- | --- | --- | --- | --- | --- | --- | --- | --- | --- | --- | --- | --- | --- | --- | --- | --- | --- | --- | --- | --- | --- | --- | --- | --- | --- | --- | --- | --- | --- | --- | --- | --- | --- | --- | --- | --- | --- | --- | --- | --- | --- | --- | --- | --- | --- | --- | --- | --- | --- | --- | --- | --- | --- | --- | --- | --- | --- | --- | --- | --- | --- | --- | --- | --- | --- | --- | --- | --- | --- | --- | --- | --- | --- | --- | --- | --- | --- | --- | --- | --- | --- | --- | --- | --- | --- | --- | --- | --- | --- | --- | --- | --- | --- | --- | --- | --- | --- | --- | --- | --- | --- | --- | --- | --- | --- | --- | --- | --- | --- | --- | --- | --- | --- | --- | --- | --- | --- | --- | --- | --- | --- | --- | --- | --- | --- | --- | --- | --- | --- | --- | --- | --- | --- | --- | --- | --- | --- | --- | --- | --- | --- | --- | --- | --- | --- | --- | --- | --- | --- | --- | --- | --- | --- | --- | --- | --- | --- | --- | --- | --- | --- | --- | --- | --- | --- | --- | --- | --- | --- | --- | --- | --- | --- | --- | --- | --- | --- | --- | --- | --- | --- | --- | --- | --- | --- | --- | --- | --- | --- | --- | --- | --- | --- | --- | --- | --- | --- | --- | --- | --- | --- | --- | --- | --- | --- | --- | --- | --- | --- | --- | --- | --- | --- | --- | --- | --- | --- | --- | --- | --- | --- | --- | --- | --- | --- | --- | --- | --- | --- | --- | --- | --- | --- | --- | --- | --- | --- | --- | --- | --- | --- | --- | --- | --- | --- | --- | --- | --- | --- | --- | --- | --- | --- | --- | --- | --- | --- | --- | --- | --- | --- | --- | --- | --- | --- | --- | --- | --- | --- | --- | --- | --- | --- | --- | --- | --- | --- | --- | --- | --- | --- | --- | --- | --- | --- | --- | --- | --- | --- | --- | --- | --- | --- | --- | --- | --- | --- | --- | --- | --- | --- | --- | --- | --- | --- | --- | --- | --- | --- | --- | --- | --- | --- | --- | --- | --- | --- | --- | --- | --- | --- | --- | --- | --- | --- | --- | --- | --- | --- | --- | --- | --- | --- | --- | --- | --- | --- | --- | --- | --- | --- | --- | --- | --- | --- | --- | --- | --- | --- | --- | --- | --- | --- | --- | --- | --- | --- | --- | --- | --- | --- | --- | --- | --- | --- | --- | --- | --- | --- | --- | --- | --- | --- | --- | --- | --- | --- | --- | --- | --- | --- | --- | --- | --- | --- | --- | --- | --- | --- | --- | --- | --- | --- | --- | --- | --- | --- | --- | --- | --- | --- | --- | --- | --- | --- | --- | --- | --- | --- | --- | --- | --- | --- | --- | --- | --- | --- | --- | --- | --- | --- | --- | --- | --- | --- | --- | --- | --- | --- | --- | --- | --- | --- | --- | --- | --- | --- | --- | --- | --- | --- | --- | --- | --- | --- | --- | --- | --- | --- | --- | --- | --- | --- | --- | --- | --- | --- | --- | --- | --- | --- | --- | --- | --- | --- | --- | --- | --- | --- | --- | --- | --- | --- | --- | --- | --- | --- | --- | --- | --- | --- | --- | --- | --- | --- | --- | --- | --- | --- | --- | --- | --- | --- | --- | --- | --- | --- | --- | --- | --- | --- | --- | --- | --- | --- | --- | --- | --- | --- | --- | --- | --- | --- | --- | --- | --- | --- | --- | --- | --- | --- | --- | --- | --- | --- | --- | --- | --- | --- | --- | --- | --- | --- | --- | --- | --- | --- | --- | --- | --- | --- | --- | --- | --- | --- | --- | --- | --- | --- | --- | --- | --- | --- | --- | --- | --- | --- | --- | --- | --- | --- | --- | --- | --- | --- | --- | --- | --- | --- | --- | --- | --- | --- | --- | --- | --- | --- | --- | --- | --- | --- | --- | --- | --- | --- | --- | --- | --- | --- | --- | --- | --- | --- | --- | --- | --- | --- | --- | --- | --- | --- | --- | --- | --- | --- | --- | --- | --- | --- | --- | --- | --- | --- | --- | --- | --- | --- | --- | --- | --- | --- | --- | --- | --- | --- | --- | --- | --- | --- | --- | --- | --- | --- | --- | --- | --- | --- | --- | --- | --- | --- | --- | --- | --- | --- | --- | --- | --- | --- | --- | --- | --- | --- | --- | --- | --- | --- | --- | --- | --- | --- | --- | --- | --- | --- | --- | --- | --- | --- | --- | --- | --- | --- | --- | --- | --- | --- | --- | --- | --- | --- | --- | --- | --- | --- | --- | --- | --- | --- | --- | --- | --- | --- | --- | --- | --- | --- | --- | --- | --- | --- | --- | --- | --- | --- | --- | --- | --- | --- | --- | --- | --- | --- | --- | --- | --- | --- | --- | --- | --- | --- | --- | --- | --- | --- | --- | --- | --- | --- | --- | --- | --- | --- | --- | --- | --- | --- | --- | --- | --- | --- | --- | --- | --- | --- | --- | --- | --- | --- | --- | --- | --- | --- | --- | --- | --- | --- | --- | --- | --- | --- | --- | --- | --- | --- | --- | --- | --- | --- | --- | --- | --- | --- | --- | --- | --- | --- | --- | --- | --- | --- | --- | --- | --- | --- | --- | --- | --- | --- | --- | --- | --- | --- | --- | --- | --- | --- | --- | --- | --- | --- | --- | --- | --- | --- | --- | --- | --- | --- | --- | --- | --- | --- | --- | --- | --- | --- | --- | --- | --- | --- | --- | --- | --- | --- | --- | --- | --- | --- | --- | --- | --- | --- | --- | --- | --- | --- | --- | --- | --- | --- | --- | --- | --- | --- | --- | --- | --- | --- | --- | --- | --- | --- | --- | --- | --- | --- | --- | --- | --- | --- | --- | --- | --- | --- | --- | --- | --- | --- | --- | --- | --- | --- | --- | --- | --- | --- | --- | --- | --- | --- | --- | --- | --- | --- | --- | --- | --- | --- | --- | --- | --- | --- | --- | --- | --- | --- | --- | --- | --- | --- | --- | --- | --- | --- | --- | --- | --- | --- | --- | --- | --- | --- | --- | --- | --- | --- | --- | --- | --- | --- | --- | --- | --- | --- | --- | --- | --- | --- | --- | --- | --- | --- | --- | --- | --- | --- | --- | --- | --- | --- | --- | --- | --- | --- | --- | --- | --- | --- | --- | --- | --- | --- | --- | --- | --- | --- | --- | --- | --- | --- | --- | --- | --- | --- | --- | --- | --- | --- | --- | --- | --- | --- | --- | --- | --- | --- | --- | --- | --- | --- | --- | --- | --- | --- | --- | --- | --- | --- | --- | --- | --- | --- | --- | --- | --- | --- | --- | --- | --- | --- | --- | --- | --- | --- | --- | --- | --- | --- | --- | --- | --- | --- | --- | --- | --- | --- | --- | --- | --- | --- | --- | --- | --- | --- | --- | --- | --- | --- | --- | --- | --- | --- | --- | --- | --- | --- | --- | --- | --- | --- | --- | --- | --- | --- | --- | --- | --- | --- | --- | --- | --- | --- | --- | --- | --- | --- | --- | --- | --- | --- | --- | --- | --- | --- | --- | --- | --- | --- | --- | --- | --- | --- | --- | --- | --- | --- | --- | --- | --- | --- | --- | --- | --- | --- | --- | --- | --- | --- | --- | --- | --- | --- | --- | --- | --- | --- | --- | --- | --- | --- | --- | --- | --- | --- | --- | --- | --- | --- | --- | --- | --- | --- | --- | --- | --- | --- | --- | --- | --- | --- | --- | --- | --- | --- | --- | --- | --- | --- | --- | --- | --- | --- | --- | --- | --- | --- | --- | --- | --- | --- | --- | --- | --- | --- | --- | --- | --- | --- | --- | --- | --- | --- | --- | --- | --- | --- | --- | --- | --- | --- | --- | --- | --- | --- | --- | --- | --- | --- | --- | --- | --- | --- | --- | --- | --- | --- | --- | --- | --- | --- | --- | --- | --- | --- | --- | --- | --- | --- | --- | --- | --- | --- | --- | --- | --- | --- | --- | --- | --- | --- | --- | --- | --- | --- | --- | --- | --- | --- | --- | --- | --- | --- | --- | --- | --- | --- | --- | --- | --- | --- | --- | --- | --- | --- | --- | --- | --- | --- | --- | --- | --- | --- | --- | --- | --- | --- | --- | --- | --- | --- | --- | --- | --- | --- | --- | --- | --- | --- | --- | --- | --- | --- | --- | --- | --- | --- | --- | --- | --- | --- | --- | --- | --- | --- | --- | --- | --- | --- | --- | --- | --- | --- | --- | --- | --- | --- | --- | --- | --- | --- | --- | --- | --- | --- | --- | --- | --- | --- | --- | --- | --- | --- | --- | --- | --- | --- | --- | --- | --- | --- | --- | --- | --- | --- | --- | --- | --- | --- | --- | --- | --- | --- | --- | --- | --- | --- | --- | --- | --- | --- | --- | --- | --- | --- | --- | --- | --- | --- | --- | --- | --- | --- | --- | --- | --- | --- | --- | --- | --- | --- | --- | --- | --- | --- | --- | --- | --- | --- | --- | --- | --- | --- | --- | --- | --- | --- | --- | --- | --- | --- | --- | --- | --- | --- | --- | --- | --- | --- | --- | --- | --- | --- | --- | --- | --- | --- | --- | --- | --- | --- | --- | --- | --- | --- | --- | --- | --- | --- | --- | --- | --- | --- | --- | --- | --- | --- | --- | --- | --- | --- | --- | --- | --- | --- | --- | --- | --- | --- | --- | --- | --- | --- | --- | --- | --- | --- | --- | --- | --- | --- | --- | --- | --- | --- | --- | --- | --- | --- | --- | --- | --- | --- | --- | --- | --- | --- | --- | --- | --- | --- | --- | --- | --- | --- | --- | --- | --- | --- | --- | --- | --- | --- | --- | --- | --- | --- | --- | --- | --- | --- | --- | --- | --- | --- | --- | --- | --- | --- | --- | --- | --- | --- | --- | --- | --- | --- | --- | --- | --- | --- | --- | --- | --- | --- | --- | --- | --- | --- | --- | --- | --- | --- | --- | --- | --- | --- | --- | --- | --- | --- | --- | --- | --- | --- | --- | --- | --- | --- | --- | --- | --- | --- | --- | --- | --- | --- | --- | --- | --- | --- | --- | --- | --- | --- | --- | --- | --- | --- | --- | --- | --- | --- | --- | --- | --- | --- | --- | --- | --- | --- | --- | --- | --- | --- | --- | --- | --- | --- | --- | --- | --- | --- | --- | --- | --- | --- | --- | --- | --- | --- | --- | --- | --- | --- | --- | --- | --- | --- | --- | --- | --- | --- | --- | --- | --- | --- | --- | --- | --- | --- | --- | --- | --- | --- | --- | --- | --- | --- | --- | --- | --- | --- | --- | --- | --- | --- | --- | --- | --- | --- | --- | --- | --- | --- | --- | --- | --- | --- | --- | --- | --- | --- | --- | --- | --- | --- | --- | --- | --- | --- | --- | --- | --- | --- | --- | --- | --- | --- | --- | --- | --- | --- | --- | --- | --- | --- | --- | --- | --- | --- | --- | --- | --- | --- | --- | --- | --- | --- | --- | --- | --- | --- | --- | --- | --- | --- | --- | --- | --- | --- | --- | --- | --- | --- | --- | --- | --- | --- | --- | --- | --- | --- | --- | --- | --- | --- | --- | --- | --- | --- | --- | --- | --- | --- | --- | --- | --- | --- | --- | --- | --- | --- | --- | --- | --- | --- | --- | --- | --- | --- | --- | --- | --- | --- | --- | --- | --- | --- | --- | --- | --- | --- | --- | --- | --- | --- | --- | --- | --- | --- | --- | --- | --- | --- | --- | --- | --- | --- | --- | --- | --- | --- | --- | --- | --- | --- | --- | --- | --- | --- | --- | --- | --- | --- | --- | --- | --- | --- | --- | --- | --- | --- | --- | --- | --- | --- | --- | --- | --- | --- | --- | --- | --- | --- | --- | --- | --- | --- | --- | --- | --- | --- | --- | --- | --- | --- | --- | --- | --- | --- | --- | --- | --- | --- | --- | --- | --- | --- | --- | --- | --- | --- | --- | --- | --- | --- | --- | --- | --- | --- | --- | --- | --- | --- | --- | --- | --- | --- | --- | --- | --- | --- | --- | --- | --- | --- | --- | --- | --- | --- | --- | --- | --- | --- | --- | --- | --- | --- | --- | --- | --- | --- | --- | --- | --- | --- | --- | --- | --- | --- | --- | --- | --- | --- | --- | --- | --- | --- | --- | --- | --- | --- | --- | --- | --- | --- | --- | --- | --- | --- | --- | --- | --- | --- | --- | --- | --- | --- | --- | --- | --- | --- | --- | --- | --- | --- | --- | --- | --- | --- | --- | --- | --- | --- | --- | --- | --- | --- | --- | --- | --- | --- | --- | --- | --- | --- | --- | --- | --- | --- | --- | --- | --- | --- | --- | --- | --- | --- | --- | --- | --- | --- | --- | --- | --- | --- | --- | --- | --- | --- | --- | --- | --- | --- | --- | --- | --- | --- | --- | --- | --- | --- | --- | --- | --- | --- | --- | --- | --- | --- | --- | --- | --- | --- | --- | --- | --- | --- | --- | --- | --- | --- | --- | --- | --- | --- | --- | --- | --- | --- | --- | --- | --- | --- | --- | --- | --- | --- | --- | --- | --- | --- | --- | --- | --- | --- | --- | --- | --- | --- | --- | --- | --- | --- | --- | --- | --- | --- | --- | --- | --- | --- | --- | --- | --- | --- | --- | --- | --- | --- | --- | --- | --- | --- | --- | --- | --- | --- | --- | --- | --- | --- | --- | --- | --- | --- | --- | --- | --- | --- | --- | --- | --- | --- | --- | --- | --- | --- | --- | --- | --- | --- | --- | --- | --- | --- | --- | --- | --- | --- | --- | --- | --- | --- | --- | --- | --- | --- | --- | --- | --- | --- | --- | --- | --- | --- | --- | --- | --- | --- | --- | --- | --- | --- | --- | --- | --- | --- | --- | --- | --- | --- | --- | --- | --- | --- | --- | --- | --- | --- | --- | --- | --- | --- | --- | --- | --- | --- | --- | --- | --- | --- | --- | --- | --- | --- | --- | --- | --- | --- | --- | --- | --- | --- | --- | --- | --- | --- | --- | --- | --- | --- | --- | --- | --- | --- | --- | --- | --- | --- | --- | --- | --- | --- | --- | --- | --- | --- | --- | --- | --- | --- | --- | --- | --- | --- | --- | --- | --- | --- | --- | --- | --- | --- | --- | --- | --- | --- | --- | --- | --- | --- | --- | --- | --- | --- | --- | --- | --- | --- | --- | --- | --- | --- | --- | --- | --- | --- | --- | --- | --- | --- | --- | --- | --- | --- | --- | --- | --- | --- | --- | --- | --- | --- | --- | --- | --- | --- | --- | --- | --- | --- | --- | --- | --- | --- | --- | --- | --- | --- | --- | --- | --- | --- | --- | --- | --- | --- | --- | --- | --- | --- | --- | --- | --- | --- | --- | --- | --- | --- | --- | --- | --- | --- | --- | --- | --- | --- | --- | --- | --- | --- | --- | --- | --- | --- | --- | --- | --- | --- | --- | --- | --- | --- | --- | --- | --- | --- | --- | --- | --- | --- | --- | --- | --- | --- | --- | --- | --- | --- | --- | --- | --- | --- | --- | --- | --- | --- | --- | --- | --- | --- | --- | --- | --- | --- | --- | --- | --- | --- | --- | --- | --- | --- | --- | --- | --- | --- | --- | --- | --- | --- | --- | --- | --- | --- | --- | --- | --- | --- | --- | --- | --- | --- | --- | --- | --- | --- | --- | --- | --- | --- | --- | --- | --- | --- | --- | --- | --- | --- | --- | --- | --- | --- | --- | --- | --- | --- | --- | --- | --- | --- | --- | --- | --- | --- | --- | --- | --- | --- | --- | --- | --- | --- | --- | --- | --- | --- | --- | --- | --- | --- | --- | --- | --- | --- | --- | --- | --- | --- | --- | --- | --- | --- | --- | --- | --- | --- | --- | --- | --- | --- | --- | --- | --- | --- | --- | --- | --- | --- | --- | --- | --- | --- | --- | --- | --- | --- | --- | --- | --- | --- | --- | --- | --- | --- | --- | --- | --- | --- | --- | --- | --- | --- | --- | --- | --- | --- | --- | --- | --- | --- | --- | --- | --- | --- | --- | --- | --- | --- | --- | --- | --- | --- | --- | --- | --- | --- | --- | --- | --- | --- | --- | --- | --- | --- | --- | --- | --- | --- | --- | --- | --- | --- | --- | --- | --- | --- | --- | --- | --- | --- | --- | --- | --- | --- | --- | --- | --- | --- | --- | --- | --- | --- | --- | --- | --- | --- | --- | --- | --- | --- | --- | --- | --- | --- | --- | --- | --- | --- | --- | --- | --- | --- | --- | --- | --- | --- | --- | --- | --- | --- | --- | --- | --- | --- | --- | --- | --- | --- | --- | --- | --- | --- | --- | --- | --- | --- | --- | --- | --- | --- | --- | --- | --- | --- | --- | --- | --- | --- | --- | --- | --- | --- | --- | --- | --- | --- | --- | --- | --- | --- | --- | --- | --- | --- | --- | --- | --- | --- | --- | --- | --- | --- | --- | --- | --- | --- | --- | --- | --- | --- | --- | --- | --- | --- | --- | --- | --- | --- | --- | --- | --- | --- | --- | --- | --- | --- | --- | --- | --- | --- | --- | --- | --- | --- | --- | --- | --- | --- | --- | --- | --- | --- | --- | --- | --- | --- | --- | --- | --- | --- | --- | --- | --- | --- | --- | --- | --- | --- | --- | --- | --- | --- | --- | --- | --- | --- | --- | --- | --- | --- | --- | --- | --- | --- | --- | --- | --- | --- | --- | --- | --- | --- | --- | --- | --- | --- | --- | --- | --- | --- | --- | --- | --- | --- | --- | --- | --- | --- | --- | --- | --- | --- | --- | --- | --- | --- | --- | --- | --- | --- | --- | --- | --- | --- | --- | --- | --- | --- | --- | --- | --- | --- | --- | --- | --- | --- | --- | --- | --- | --- | --- | --- | --- | --- | --- | --- | --- | --- | --- | --- | --- | --- | --- | --- | --- | --- | --- | --- | --- | --- | --- | --- | --- | --- | --- | --- | --- | --- | --- | --- | --- | --- | --- | --- | --- | --- | --- | --- | --- | --- | --- | --- | --- | --- | --- | --- | --- | --- | --- | --- | --- | --- | --- | --- | --- | --- | --- | --- | --- | --- | --- | --- | --- | --- | --- | --- | --- | --- | --- | --- | --- | --- | --- | --- | --- | --- | --- | --- | --- | --- | --- | --- | --- | --- | --- | --- | --- | --- | --- | --- | --- | --- | --- | --- | --- | --- | --- | --- | --- | --- | --- | --- | --- | --- | --- | --- | --- | --- | --- | --- | --- | --- | --- | --- | --- | --- | --- | --- | --- | --- | --- | --- | --- | --- | --- | --- | --- | --- | --- | --- | --- | --- | --- | --- | --- | --- | --- | --- | --- | --- | --- | --- | --- | --- | --- | --- | --- | --- | --- | --- | --- | --- | --- | --- | --- | --- | --- | --- | --- | --- | --- | --- | --- | --- | --- | --- | --- | --- | --- | --- | --- | --- | --- | --- | --- | --- | --- | --- | --- | --- | --- | --- | --- | --- | --- | --- | --- | --- | --- | --- | --- | --- | --- | --- | --- | --- | --- | --- | --- | --- | --- | --- | --- | --- | --- | --- | --- | --- | --- | --- | --- | --- | --- | --- | --- | --- | --- | --- | --- | --- | --- | --- | --- | --- | --- | --- | --- | --- | --- | --- | --- | --- | --- | --- | --- | --- | --- | --- | --- | --- | --- | --- | --- | --- | --- | --- | --- | --- | --- | --- | --- | --- | --- | --- | --- | --- | --- | --- | --- | --- | --- | --- | --- | --- | --- | --- | --- | --- | --- | --- | --- | --- | --- | --- | --- | --- | --- | --- | --- | --- | --- | --- | --- | --- | --- | --- | --- | --- | --- | --- | --- | --- | --- | --- | --- | --- | --- | --- | --- | --- | --- | --- | --- | --- | --- | --- | --- | --- | --- | --- | --- | --- | --- | --- | --- | --- | --- | --- | --- | --- | --- | --- | --- | --- | --- | --- | --- | --- | --- | --- | --- | --- | --- | --- | --- | --- | --- | --- | --- | --- | --- | --- | --- | --- | --- | --- | --- | --- | --- | --- | --- | --- | --- | --- | --- | --- | --- | --- | --- | --- | --- | --- | --- | --- | --- | --- | --- | --- | --- | --- | --- | --- | --- | --- | --- | --- | --- | --- | --- | --- | --- | --- | --- | --- | --- | --- | --- | --- | --- | --- | --- | --- | --- | --- | --- | --- | --- | --- | --- | --- | --- | --- | --- | --- | --- | --- | --- | --- | --- | --- | --- | --- | --- | --- | --- | --- | --- | --- | --- | --- | --- | --- | --- | --- | --- | --- | --- | --- | --- | --- | --- | --- | --- | --- | --- | --- | --- | --- | --- | --- | --- | --- | --- | --- | --- | --- | --- | --- | --- | --- | --- | --- | --- | --- | --- | --- | --- | --- | --- | --- | --- | --- | --- | --- | --- | --- | --- | --- | --- | --- | --- | --- | --- | --- | --- | --- | --- | --- | --- | --- | --- | --- | --- | --- | --- | --- | --- | --- | --- | --- | --- | --- | --- | --- | --- | --- | --- | --- | --- | --- | --- | --- | --- | --- | --- | --- | --- | --- | --- | --- | --- | --- | --- | --- | --- | --- | --- | --- | --- | --- | --- | --- | --- | --- | --- | --- | --- | --- | --- | --- | --- | --- | --- | --- | --- | --- | --- | --- | --- | --- | --- | --- | --- | --- | --- | --- | --- | --- | --- | --- | --- | --- | --- | --- | --- | --- | --- | --- | --- | --- | --- | --- | --- | --- | --- | --- | --- | --- | --- | --- | --- | --- | --- | --- | --- | --- | --- | --- | --- | --- | --- | --- | --- | --- | --- | --- | --- | --- | --- | --- | --- | --- | --- | --- | --- | --- | --- | --- | --- | --- | --- | --- | --- | --- | --- | --- | --- | --- | --- | --- | --- | --- | --- | --- | --- | --- | --- | --- | --- | --- | --- | --- | --- | --- | --- | --- | --- | --- | --- | --- | --- | --- | --- | --- | --- | --- | --- | --- | --- | --- | --- | --- | --- | --- | --- | --- | --- | --- | --- | --- | --- | --- | --- | --- | --- | --- | --- | --- | --- | --- | --- | --- | --- | --- | --- | --- | --- | --- | --- | --- | --- | --- | --- | --- | --- | --- | --- | --- | --- | --- | --- | --- | --- | --- | --- | --- | --- | --- | --- | --- | --- | --- | --- | --- | --- | --- | --- | --- | --- | --- | --- | --- | --- | --- | --- | --- | --- | --- | --- | --- | --- | --- | --- | --- | --- | --- | --- | --- | --- | --- | --- | --- | --- | --- | --- | --- | --- | --- | --- | --- | --- | --- | --- | --- | --- | --- | --- | --- | --- | --- | --- | --- | --- | --- | --- | --- | --- | --- | --- | --- | --- | --- | --- | --- | --- | --- | --- | --- | --- | --- | --- | --- | --- | --- | --- | --- | --- | --- | --- | --- | --- | --- | --- | --- | --- | --- | --- | --- | --- | --- | --- | --- | --- | --- | --- | --- | --- | --- | --- | --- | --- | --- | --- | --- | --- | --- | --- | --- | --- | --- | --- | --- | --- | --- | --- | --- | --- | --- | --- | --- | --- | --- | --- | --- | --- | --- | --- | --- | --- | --- | --- | --- | --- | --- | --- | --- | --- | --- | --- | --- | --- | --- | --- | --- | --- | --- | --- | --- | --- | --- | --- | --- | --- | --- | --- | --- | --- | --- | --- | --- | --- | --- | --- | --- | --- | --- | --- | --- | --- | --- | --- | --- | --- | --- | --- | --- | --- | --- | --- | --- | --- | --- | --- | --- | --- | --- | --- | --- | --- | --- | --- | --- | --- | --- | --- | --- | --- | --- | --- | --- | --- | --- | --- | --- | --- | --- | --- | --- | --- | --- | --- | --- | --- | --- | --- | --- | --- | --- | --- | --- | --- | --- | --- | --- | --- | --- | --- | --- | --- | --- | --- | --- | --- | --- | --- | --- | --- | --- | --- | --- | --- | --- | --- | --- | --- | --- | --- | --- | --- | --- | --- | --- | --- | --- | --- | --- | --- | --- | --- | --- | --- | --- | --- | --- |
|  |  |  |  |  |  |  |  |  |  |

*Values in the column “Survival direction”: "-" = more survivors in the low-expression third. "+" = more survivors in the higher-expression third.

**Gray background – genes that are associated with the Immune system.

| **Supplementary table 3: Survival analysis results for highest CVR genes group** | | | | | | | |  |  |
| --- | --- | --- | --- | --- | --- | --- | --- | --- | --- |
|  |  |  |  | **Survival (Logrank test)** | | | | **log norm** |  |
|  |  |  |  | **Normalized** | | **Un-normalized** | | **/ raw** |  |
| **#** | **Gene Name** | **CVR** | **Survival direction*** | **Nominal** | **Bonferroni correction** | **Nominal** | **Bonferroni correction** | **nominal logrank** | **Immune response**** |
| 1 | CCNDBP1 | 4.14 | + | 5.09E-03 | 1 | 1.02E-05 | 5.11E-03 | 6.21 | Y |
| 2 | TMEM184C | 4.71 | + | 5.41E-03 | 1 | 1.46E-04 | 7.31E-02 | 3.61 | N |
| 3 | UBR3 | 4.11 | + | 2.41E-02 | 1 | 2.86E-04 | 1.43E-01 | 4.43 | N |
| 4 | PRMT10 | 4.25 | + | 4.01E-02 | 1 | 2.68E-04 | 1.34E-01 | 5.01 | N |
| 5 | FBXW2 | 4.84 | + | 4.24E-02 | 1 | 7.57E-04 | 3.78E-01 | 4.03 | N |
| 6 | OSBP | 4.23 | + | 4.30E-02 | 1 | 1.65E-04 | 8.26E-02 | 5.56 | N |
| 7 | STAT5B | 4.35 | + | 4.43E-02 | 1 | 2.13E-06 | 1.07E-03 | 9.94 | Y |
| 8 | LOC647979 | 4.36 | + | 4.78E-02 | 1 | 4.60E-03 | 1 | 2.34 | N |
| 9 | MYST2 | 4.20 | + | 5.66E-02 | 1 | 3.75E-05 | 1.87E-02 | 7.32 | N |
| 10 | ZBTB4 | 4.17 | + | 6.20E-02 | 1 | 1.65E-06 | 8.23E-04 | 10.54 | N |
| 11 | AP3B1 | 4.50 | + | 6.49E-02 | 1 | 3.23E-03 | 1.00E+00 | 3.00 | N |
| 12 | MFAP1 | 4.53 | + | 6.58E-02 | 1 | 5.19E-05 | 2.60E-02 | 7.15 | N |
| 13 | CA5B | 4.20 | + | 7.32E-02 | 1 | 6.49E-04 | 3.24E-01 | 4.73 | N |
| 14 | HADHB | 4.26 | + | 7.69E-02 | 1 | 6.18E-04 | 3.09E-01 | 4.82 | N |
| 15 | UBE3A | 4.13 | + | 8.75E-02 | 1 | 6.96E-03 | 1 | 2.53 | N |
| 16 | GOSR1 | 4.42 | + | 9.33E-02 | 1 | 1.52E-04 | 7.61E-02 | 6.42 | N |
| 17 | FOXO1 | 4.20 | + | 9.37E-02 | 1 | 1.13E-03 | 5.67E-01 | 4.41 | Y |
| 18 | ARPP19 | 4.15 | + | 9.56E-02 | 1 | 7.99E-06 | 4.00E-03 | 9.39 | N |
| 19 | ZNF747 | 4.14 | + | 1.01E-01 | 1 | 5.38E-03 | 1 | 2.93 | N |
| 20 | NARG2 | 4.55 | + | 1.10E-01 | 1 | 1.73E-02 | 1 | 1.86 | N |
| 21 | MECP2 | 4.30 | + | 1.11E-01 | 1 | 4.55E-04 | 2.27E-01 | 5.50 | N |
| 22 | UBE2D3 | 4.23 | + | 1.12E-01 | 1 | 1.14E-03 | 5.71E-01 | 4.58 | N |
| 23 | C4orf41 | 4.19 | + | 1.15E-01 | 1 | 3.69E-02 | 1 | 1.13 | N |
| 24 | CTR9 | 4.16 | + | 1.15E-01 | 1 | 8.01E-05 | 4.01E-02 | 7.27 | N |
| 25 | TRPC4AP | 4.17 | - | 1.19E-01 | 1 | 2.65E-09 | 1.32E-06 | 17.62 | Y |
| 26 | FUBP3 | 4.26 | + | 1.21E-01 | 1 | 1.77E-04 | 8.86E-02 | 6.52 | N |
| 27 | BBS2 | 4.13 | + | 1.22E-01 | 1 | 7.60E-01 | 1 | -1.83 | N |
| 28 | SUV420H1 | 4.13 | + | 1.24E-01 | 1 | 5.24E-03 | 1 | 3.16 | N |
| 29 | STARD7 | 4.90 | + | 1.25E-01 | 1 | 5.46E-02 | 1 | 0.83 | N |
| 30 | C9orf156 | 4.30 | + | 1.26E-01 | 1 | 1.36E-04 | 6.79E-02 | 6.84 | N |
| 31 | CUEDC1 | 4.13 | + | 1.27E-01 | 1 | 4.82E-02 | 1 | 0.97 | N |
| 32 | TFCP2 | 4.87 | + | 1.29E-01 | 1 | 8.73E-01 | 1 | -1.91 | N |
| 33 | RNF214 | 4.22 | + | 1.29E-01 | 1 | 1.74E-01 | 1 | -0.30 | N |
| 34 | ASB8 | 4.79 | + | 1.33E-01 | 1 | 1.75E-03 | 8.75E-01 | 4.33 | N |
| 35 | LOC729082 | 4.14 | + | 1.35E-01 | 1 | 1.69E-02 | 1 | 2.08 | N |
| 36 | UVRAG | 4.10 | + | 1.38E-01 | 1 | 2.35E-02 | 1 | 1.77 | N |
| 37 | HNRNPK | 5.40 | + | 1.42E-01 | 1 | 1.78E-05 | 8.90E-03 | 8.98 | N |
| 38 | PHKB | 4.43 | + | 1.47E-01 | 1 | 1.89E-04 | 9.47E-02 | 6.65 | N |
| 39 | HDHD2 | 4.20 | + | 1.48E-01 | 1 | 4.62E-06 | 2.31E-03 | 10.37 | N |
| 40 | CNOT4 | 4.81 | + | 1.51E-01 | 1 | 5.95E-04 | 2.97E-01 | 5.54 | N |
| 41 | SNX1 | 5.40 | + | 1.54E-01 | 1 | 4.83E-02 | 1 | 1.16 | N |
| 42 | CTCF | 4.72 | + | 1.54E-01 | 1 | 4.53E-05 | 2.27E-02 | 8.13 | N |
| 43 | OSBPL9 | 4.35 | + | 1.55E-01 | 1 | 8.43E-02 | 1 | 0.61 | N |
| 44 | FBXO9 | 4.18 | + | 1.56E-01 | 1 | 3.66E-02 | 1 | 1.45 | N |
| 45 | GPBP1 | 4.10 | + | 1.58E-01 | 1 | 1.08E-04 | 5.40E-02 | 7.29 | N |
| 46 | TERF2 | 4.19 | + | 1.58E-01 | 1 | 1.80E-02 | 1 | 2.18 | N |
| 47 | WDR33 | 4.41 | + | 1.59E-01 | 1 | 5.44E-01 | 1 | -1.23 | N |
| 48 | KLHL26 | 4.24 | + | 1.60E-01 | 1 | 9.00E-04 | 4.50E-01 | 5.18 | N |
| 49 | PLRG1 | 5.26 | + | 1.60E-01 | 1 | 9.26E-07 | 4.63E-04 | 12.06 | N |
| 50 | METAP1 | 4.31 | + | 1.61E-01 | 1 | 5.98E-04 | 2.99E-01 | 5.60 | N |
| 51 | WDR20 | 4.19 | + | 1.61E-01 | 1 | 1.45E-03 | 7.24E-01 | 4.71 | N |
| 52 | BAHD1 | 4.14 | + | 1.64E-01 | 1 | 1.49E-02 | 1 | 2.40 | N |
| 53 | PSMD7 | 4.19 | - | 1.67E-01 | 1 | 2.75E-06 | 1.37E-03 | 11.01 | Y |
| 54 | ZNF2 | 4.23 | + | 1.68E-01 | 1 | 1.52E-01 | 1 | 0.10 | N |
| 55 | KIAA2026 | 4.19 | + | 1.69E-01 | 1 | 2.85E-02 | 1 | 1.78 | N |
| 56 | MAP2K5 | 4.13 | + | 1.72E-01 | 1 | 1.97E-02 | 1 | 2.17 | N |
| 57 | UBQLN1 | 4.48 | + | 1.72E-01 | 1 | 7.54E-04 | 3.77E-01 | 5.43 | N |
| 58 | PAFAH1B1 | 4.49 | + | 1.76E-01 | 1 | 1.92E-03 | 9.58E-01 | 4.52 | N |
| 59 | BCL2L13 | 4.56 | + | 1.78E-01 | 1 | 1.40E-02 | 1 | 2.55 | N |
| 60 | MED9 | 4.71 | + | 1.78E-01 | 1 | 4.74E-01 | 1 | -0.98 | N |
| 61 | SMG7 | 4.13 | + | 1.80E-01 | 1 | 2.17E-01 | 1 | -0.19 | N |
| 62 | FAM122B | 4.14 | - | 1.84E-01 | 1 | 1.65E-06 | 8.25E-04 | 11.62 | N |
| 63 | NCOA6 | 4.17 | + | 1.85E-01 | 1 | 1.12E-01 | 1 | 0.51 | Y |
| 64 | CCDC25 | 4.27 | + | 1.88E-01 | 1 | 1.41E-04 | 7.05E-02 | 7.19 | N |
| 65 | H2AFV | 4.36 | + | 1.92E-01 | 1 | 1.41E-04 | 7.03E-02 | 7.22 | N |
| 66 | OGFOD1 | 4.52 | - | 1.93E-01 | 1 | 3.58E-03 | 1 | 3.99 | N |
| 67 | EXOC1 | 4.55 | + | 2.01E-01 | 1 | 6.86E-01 | 1 | -1.23 | N |
| 68 | SMU1 | 4.30 | + | 2.01E-01 | 1 | 5.25E-08 | 2.63E-05 | 15.16 | N |
| 69 | RNF10 | 4.68 | - | 2.02E-01 | 1 | 3.57E-07 | 1.78E-04 | 13.25 | N |
| 70 | SIN3A | 4.63 | + | 2.04E-01 | 1 | 3.93E-03 | 1 | 3.95 | N |
| 71 | C6orf130 | 4.42 | + | 2.05E-01 | 1 | 6.42E-02 | 1 | 1.16 | N |
| 72 | USP39 | 4.65 | - | 2.11E-01 | 1 | 1.93E-05 | 9.66E-03 | 9.30 | N |
| 73 | IPO8 | 4.22 | + | 2.13E-01 | 1 | 2.41E-03 | 1 | 4.48 | N |
| 74 | ZFR | 4.32 | + | 2.14E-01 | 1 | 5.92E-04 | 2.96E-01 | 5.89 | N |
| 75 | BCDIN3D | 4.46 | + | 2.15E-01 | 1 | 2.63E-03 | 1 | 4.41 | N |
| 76 | FBXO7 | 4.78 | + | 2.17E-01 | 1 | 8.28E-04 | 4.14E-01 | 5.57 | N |
| 77 | C19orf2 | 4.69 | + | 2.26E-01 | 1 | 1.41E-03 | 7.05E-01 | 5.08 | N |
| 78 | TADA2B | 4.67 | + | 2.27E-01 | 1 | 8.21E-02 | 1 | 1.02 | N |
| 79 | PRKRA | 4.19 | - | 2.31E-01 | 1 | 3.74E-03 | 1 | 4.12 | Y |
| 80 | KIAA0141 | 4.17 | + | 2.36E-01 | 1 | 7.46E-01 | 1 | -1.15 | N |
| 81 | USP30 | 4.29 | + | 2.39E-01 | 1 | 7.74E-03 | 1 | 3.43 | N |
| 82 | ARIH1 | 4.52 | + | 2.41E-01 | 1 | 7.57E-02 | 1 | 1.16 | N |
| 83 | RALBP1 | 4.20 | + | 2.44E-01 | 1 | 5.65E-03 | 1 | 3.77 | N |
| 84 | DDX1 | 4.31 | + | 2.45E-01 | 1 | 2.11E-04 | 1.05E-01 | 7.06 | N |
| 85 | ZNF324 | 4.23 | + | 2.48E-01 | 1 | 1.42E-01 | 1 | 0.56 | N |
| 86 | RNF34 | 4.29 | - | 2.48E-01 | 1 | 6.99E-08 | 3.49E-05 | 15.08 | N |
| 87 | C20orf11 | 4.59 | + | 2.51E-01 | 1 | 5.25E-02 | 1 | 1.57 | N |
| 88 | MCPH1 | 4.63 | + | 2.53E-01 | 1 | 6.45E-02 | 1 | 1.36 | N |
| 89 | WBP4 | 4.20 | + | 2.55E-01 | 1 | 7.62E-02 | 1 | 1.21 | N |
| 90 | ADNP | 4.36 | + | 2.56E-01 | 1 | 1.49E-01 | 1 | 0.54 | N |
| 91 | HSDL1 | 4.11 | + | 2.57E-01 | 1 | 1.41E-02 | 1 | 2.90 | N |
| 92 | UBE2V1 | 4.23 | - | 2.57E-01 | 1 | 9.00E-08 | 4.50E-05 | 14.87 | N |
| 93 | NCBP2 | 4.44 | - | 2.57E-01 | 1 | 9.15E-04 | 4.57E-01 | 5.64 | N |
| 94 | LRRC57 | 5.05 | + | 2.59E-01 | 1 | 3.15E-01 | 1 | -0.20 | N |
| 95 | RFT1 | 4.17 | - | 2.63E-01 | 1 | 1.41E-02 | 1 | 2.93 | N |
| 96 | ZNF765 | 4.12 | + | 2.70E-01 | 1 | 2.09E-03 | 1 | 4.86 | N |
| 97 | MTFMT | 4.61 | + | 2.70E-01 | 1 | 3.86E-03 | 1 | 4.25 | N |
| 98 | SLBP | 4.12 | - | 2.72E-01 | 1 | 2.65E-01 | 1 | 0.03 | N |
| 99 | CAB39 | 4.52 | + | 2.75E-01 | 1 | 2.74E-02 | 1 | 2.31 | N |
| 100 | C20orf43 | 5.21 | - | 2.76E-01 | 1 | 2.32E-01 | 1 | 0.17 | N |
| 101 | PEX7 | 4.34 | + | 2.76E-01 | 1 | 1.16E-03 | 5.82E-01 | 5.47 | N |
| 102 | INTS12 | 4.78 | + | 2.80E-01 | 1 | 5.46E-02 | 1 | 1.64 | N |
| 103 | CSTF1 | 4.59 | + | 2.83E-01 | 1 | 2.84E-03 | 1 | 4.60 | N |
| 104 | COQ7 | 4.79 | + | 2.86E-01 | 1 | 2.87E-02 | 1 | 2.30 | N |
| 105 | CDYL | 4.65 | + | 2.87E-01 | 1 | 1.22E-01 | 1 | 0.85 | N |
| 106 | RBM23 | 4.46 | + | 2.89E-01 | 1 | 2.99E-03 | 1 | 4.57 | N |
| 107 | YWHAB | 4.36 | + | 2.96E-01 | 1 | 1.02E-01 | 1 | 1.07 | Y |
| 108 | PICALM | 4.68 | + | 2.97E-01 | 1 | 6.75E-02 | 1 | 1.48 | N |
| 109 | MBTPS1 | 4.91 | + | 2.99E-01 | 1 | 3.85E-03 | 1 | 4.35 | N |
| 110 | PREP | 4.69 | - | 2.99E-01 | 1 | 1.58E-01 | 1 | 0.64 | N |
| 111 | HNRNPA0 | 4.35 | + | 3.01E-01 | 1 | 3.68E-03 | 1 | 4.41 | N |
| 112 | CLPX | 4.33 | + | 3.02E-01 | 1 | 1.37E-02 | 1 | 3.09 | N |
| 113 | ASB7 | 4.33 | + | 3.03E-01 | 1 | 3.83E-03 | 1 | 4.37 | N |
| 114 | ARFGAP2 | 4.97 | + | 3.09E-01 | 1 | 5.47E-01 | 1 | -0.57 | N |
| 115 | PPFIA1 | 4.51 | + | 3.09E-01 | 1 | 4.32E-02 | 1 | 1.97 | N |
| 116 | COX4NB | 4.15 | - | 3.10E-01 | 1 | 1.58E-02 | 1 | 2.98 | N |
| 117 | GPBP1L1 | 4.91 | + | 3.11E-01 | 1 | 1.17E-01 | 1 | 0.98 | N |
| 118 | POLR2B | 4.76 | + | 3.16E-01 | 1 | 4.05E-02 | 1 | 2.05 | N |
| 119 | INTS5 | 4.31 | + | 3.16E-01 | 1 | 3.74E-04 | 1.87E-01 | 6.74 | N |
| 120 | IRAK4 | 4.17 | + | 3.16E-01 | 1 | 9.91E-03 | 1 | 3.46 | Y |
| 121 | GRSF1 | 4.50 | + | 3.17E-01 | 1 | 9.25E-02 | 1 | 1.23 | N |
| 122 | HNRNPL | 5.08 | - | 3.18E-01 | 1 | 2.41E-08 | 1.20E-05 | 16.40 | N |
| 123 | PSEN1 | 4.17 | + | 3.21E-01 | 1 | 3.08E-04 | 1.54E-01 | 6.95 | Y |
| 124 | PSMD3 | 4.19 | - | 3.22E-01 | 1 | 4.32E-05 | 2.16E-02 | 8.92 | Y |
| 125 | N4BP1 | 6.20 | + | 3.24E-01 | 1 | 2.85E-02 | 1 | 2.43 | N |
| 126 | MED29 | 4.31 | + | 3.26E-01 | 1 | 5.71E-01 | 1 | -0.56 | N |
| 127 | RNPS1 | 4.10 | - | 3.26E-01 | 1 | 1.70E-06 | 8.50E-04 | 12.16 | N |
| 128 | ZC3H18 | 4.30 | + | 3.28E-01 | 1 | 7.86E-01 | 1 | -0.87 | N |
| 129 | HUS1 | 4.66 | + | 3.28E-01 | 1 | 8.69E-01 | 1 | -0.98 | N |
| 130 | NRD1 | 4.36 | - | 3.28E-01 | 1 | 8.50E-05 | 4.25E-02 | 8.26 | N |
| 131 | BSDC1 | 4.24 | + | 3.32E-01 | 1 | 1.37E-01 | 1 | 0.89 | N |
| 132 | GLYR1 | 4.48 | + | 3.33E-01 | 1 | 4.06E-02 | 1 | 2.10 | N |
| 133 | NMT1 | 4.26 | - | 3.35E-01 | 1 | 2.32E-05 | 1.16E-02 | 9.58 | N |
| 134 | RAD23B | 4.29 | + | 3.40E-01 | 1 | 1.17E-02 | 1 | 3.37 | N |
| 135 | EIF2B1 | 4.61 | - | 3.40E-01 | 1 | 1.79E-08 | 8.95E-06 | 16.76 | N |
| 136 | ARCN1 | 4.23 | + | 3.41E-01 | 1 | 9.76E-02 | 1 | 1.25 | N |
| 137 | NAPG | 4.19 | + | 3.44E-01 | 1 | 8.31E-01 | 1 | -0.88 | N |
| 138 | NAT10 | 4.53 | - | 3.44E-01 | 1 | 9.22E-04 | 4.61E-01 | 5.92 | N |
| 139 | ASB1 | 4.34 | + | 3.49E-01 | 1 | 2.86E-01 | 1 | 0.20 | N |
| 140 | DDX19B | 4.22 | + | 3.50E-01 | 1 | 2.90E-02 | 1 | 2.49 | N |
| 141 | ZNF317 | 5.27 | + | 3.52E-01 | 1 | 3.26E-01 | 1 | 0.08 | N |
| 142 | FBXL20 | 4.27 | + | 3.55E-01 | 1 | 1.76E-01 | 1 | 0.70 | N |
| 143 | AK3 | 4.14 | + | 3.60E-01 | 1 | 1.81E-02 | 1 | 2.99 | N |
| 144 | RABEP1 | 4.69 | + | 3.61E-01 | 1 | 5.86E-01 | 1 | -0.48 | N |
| 145 | PCTP | 4.12 | + | 3.61E-01 | 1 | 9.60E-01 | 1 | -0.98 | N |
| 146 | ZKSCAN5 | 4.50 | + | 3.65E-01 | 1 | 5.28E-01 | 1 | -0.37 | N |
| 147 | DNASE1L1 | 4.21 | + | 3.66E-01 | 1 | 1.44E-01 | 1 | 0.94 | N |
| 148 | ARIH2 | 4.59 | + | 3.67E-01 | 1 | 1.82E-02 | 1 | 3.01 | N |
| 149 | RAB11A | 4.53 | + | 3.72E-01 | 1 | 1.39E-03 | 6.93E-01 | 5.59 | N |
| 150 | ARHGAP21 | 4.15 | + | 3.73E-01 | 1 | 8.49E-01 | 1 | -0.82 | N |
| 151 | FAM82A2 | 4.83 | + | 3.73E-01 | 1 | 7.57E-03 | 1 | 3.90 | N |
| 152 | DHX36 | 4.20 | + | 3.75E-01 | 1 | 1.02E-01 | 1 | 1.30 | Y |
| 153 | UBE4A | 4.22 | + | 3.77E-01 | 1 | 4.31E-01 | 1 | -0.13 | N |
| 154 | C12orf43 | 4.55 | + | 3.77E-01 | 1 | 6.99E-02 | 1 | 1.69 | N |
| 155 | VAPB | 4.33 | + | 3.80E-01 | 1 | 4.11E-02 | 1 | 2.22 | N |
| 156 | KLHL12 | 4.25 | + | 3.80E-01 | 1 | 2.44E-01 | 1 | 0.44 | N |
| 157 | RBM14 | 4.29 | + | 3.81E-01 | 1 | 9.24E-01 | 1 | -0.89 | N |
| 158 | BECN1 | 4.33 | + | 3.82E-01 | 1 | 6.26E-01 | 1 | -0.50 | N |
| 159 | CUL4A | 5.01 | + | 3.84E-01 | 1 | 4.37E-01 | 1 | -0.13 | N |
| 160 | ARMC8 | 4.49 | + | 3.85E-01 | 1 | 4.28E-01 | 1 | -0.11 | N |
| 161 | TTC19 | 4.39 | + | 3.86E-01 | 1 | 1.35E-02 | 1 | 3.36 | N |
| 162 | CWC27 | 4.16 | + | 3.86E-01 | 1 | 5.93E-02 | 1 | 1.87 | N |
| 163 | FEM1A | 4.76 | + | 3.96E-01 | 1 | 1.36E-02 | 1 | 3.37 | N |
| 164 | DNM1L | 4.30 | = | 3.98E-01 | 1 | 4.46E-06 | 2.23E-03 | 11.40 | N |
| 165 | ELAC1 | 4.43 | + | 4.02E-01 | 1 | 4.93E-01 | 1 | -0.20 | N |
| 166 | SAR1A | 4.30 | - | 4.04E-01 | 1 | 3.58E-01 | 1 | 0.12 | N |
| 167 | ZNF200 | 4.50 | + | 4.05E-01 | 1 | 9.80E-01 | 1 | -0.88 | N |
| 168 | AMBRA1 | 4.93 | + | 4.06E-01 | 1 | 8.77E-01 | 1 | -0.77 | N |
| 169 | USP10 | 4.69 | + | 4.06E-01 | 1 | 2.03E-02 | 1 | 3.00 | N |
| 170 | SPG21 | 4.44 | - | 4.12E-01 | 1 | 8.16E-01 | 1 | -0.68 | Y |
| 171 | C13orf23 | 4.14 | + | 4.14E-01 | 1 | 5.40E-01 | 1 | -0.26 | N |
| 172 | UBAP1 | 4.18 | + | 4.17E-01 | 1 | 7.24E-02 | 1 | 1.75 | N |
| 173 | ELAVL1 | 4.98 | - | 4.18E-01 | 1 | 3.60E-03 | 1 | 4.75 | N |
| 174 | HMG20A | 4.93 | + | 4.18E-01 | 1 | 3.47E-01 | 1 | 0.19 | N |
| 175 | SAMD4B | 4.17 | + | 4.22E-01 | 1 | 9.64E-01 | 1 | -0.83 | N |
| 176 | AP2B1 | 4.36 | + | 4.23E-01 | 1 | 1.74E-02 | 1 | 3.19 | N |
| 177 | CRCP | 4.55 | + | 4.27E-01 | 1 | 2.48E-01 | 1 | 0.54 | N |
| 178 | DAZAP2 | 4.75 | + | 4.33E-01 | 1 | 2.54E-03 | 1 | 5.14 | N |
| 179 | LARP7 | 4.17 | + | 4.37E-01 | 1 | 6.41E-01 | 1 | -0.38 | N |
| 180 | C17orf80 | 4.25 | + | 4.39E-01 | 1 | 9.80E-02 | 1 | 1.50 | N |
| 181 | C2orf42 | 4.36 | + | 4.39E-01 | 1 | 4.55E-01 | 1 | -0.03 | N |
| 182 | EIF4G2 | 4.42 | + | 4.42E-01 | 1 | 4.70E-01 | 1 | -0.06 | N |
| 183 | HAUS2 | 4.45 | + | 4.43E-01 | 1 | 9.70E-02 | 1 | 1.52 | N |
| 184 | WDR92 | 4.33 | + | 4.43E-01 | 1 | 4.33E-01 | 1 | 0.02 | N |
| 185 | ZCCHC4 | 4.30 | + | 4.46E-01 | 1 | 5.37E-01 | 1 | -0.19 | N |
| 186 | ANKRD27 | 4.45 | + | 4.46E-01 | 1 | 3.89E-02 | 1 | 2.44 | N |
| 187 | DDX23 | 4.59 | + | 4.51E-01 | 1 | 7.05E-01 | 1 | -0.45 | N |
| 188 | MAP4K3 | 4.21 | + | 4.56E-01 | 1 | 8.93E-01 | 1 | -0.67 | N |
| 189 | PPM1B | 4.40 | + | 4.57E-01 | 1 | 3.04E-01 | 1 | 0.41 | N |
| 190 | VPS33B | 4.93 | + | 4.58E-01 | 1 | 1.49E-03 | 7.43E-01 | 5.73 | N |
| 191 | PDCD6IP | 4.30 | + | 4.60E-01 | 1 | 1.80E-01 | 1 | 0.94 | N |
| 192 | HNRNPH2 | 4.32 | + | 4.61E-01 | 1 | 7.04E-03 | 1 | 4.18 | N |
| 193 | WDR53 | 4.14 | - | 4.62E-01 | 1 | 4.07E-03 | 1 | 4.73 | N |
| 194 | AIMP1 | 4.36 | + | 4.63E-01 | 1 | 1.85E-01 | 1 | 0.92 | Y |
| 195 | TRA2B | 4.11 | + | 4.65E-01 | 1 | 3.35E-01 | 1 | 0.33 | N |
| 196 | ZC3H14 | 4.21 | + | 4.70E-01 | 1 | 4.50E-03 | 1 | 4.65 | N |
| 197 | VTI1A | 4.55 | + | 4.73E-01 | 1 | 1.21E-02 | 1 | 3.66 | N |
| 198 | DDX47 | 4.83 | - | 4.77E-01 | 1 | 4.03E-03 | 1 | 4.77 | N |
| 199 | C10orf76 | 4.53 | + | 4.77E-01 | 1 | 5.85E-02 | 1 | 2.10 | N |
| 200 | ZNF564 | 4.49 | + | 4.78E-01 | 1 | 3.52E-03 | 1 | 4.91 | N |
| 201 | SAPS3 | 5.77 | + | 4.80E-01 | 1 | 1.07E-01 | 1 | 1.50 | N |
| 202 | WDR1 | 4.57 | + | 4.81E-01 | 1 | 9.13E-01 | 1 | -0.64 | N |
| 203 | C10orf26 | 4.61 | + | 4.84E-01 | 1 | 9.22E-02 | 1 | 1.66 | N |
| 204 | DNAJA2 | 4.65 | + | 4.85E-01 | 1 | 6.90E-04 | 3.45E-01 | 6.56 | N |
| 205 | CDK13 | 4.51 | + | 4.85E-01 | 1 | 2.87E-01 | 1 | 0.53 | N |
| 206 | RPP14 | 4.16 | + | 4.85E-01 | 1 | 6.40E-03 | 1 | 4.33 | N |
| 207 | INTS4 | 4.13 | + | 4.87E-01 | 1 | 4.69E-01 | 1 | 0.04 | N |
| 208 | GGNBP2 | 4.52 | + | 4.88E-01 | 1 | 7.67E-01 | 1 | -0.45 | N |
| 209 | C8orf41 | 4.17 | + | 4.88E-01 | 1 | 5.29E-01 | 1 | -0.08 | N |
| 210 | TLK1 | 4.80 | + | 4.93E-01 | 1 | 7.49E-01 | 1 | -0.42 | N |
| 211 | COG7 | 4.16 | + | 4.95E-01 | 1 | 2.28E-01 | 1 | 0.78 | N |
| 212 | PITPNB | 4.14 | + | 4.95E-01 | 1 | 3.92E-01 | 1 | 0.23 | N |
| 213 | QRICH1 | 4.95 | + | 4.97E-01 | 1 | 4.75E-01 | 1 | 0.04 | N |
| 214 | PRPF19 | 4.14 | + | 5.03E-01 | 1 | 2.35E-02 | 1 | 3.07 | N |
| 215 | SUDS3 | 4.73 | = | 5.04E-01 | 1 | 2.27E-03 | 1 | 5.40 | N |
| 216 | VAPA | 4.17 | + | 5.07E-01 | 1 | 2.59E-01 | 1 | 0.67 | N |
| 217 | GGPS1 | 4.17 | - | 5.08E-01 | 1 | 2.19E-01 | 1 | 0.84 | N |
| 218 | NFYC | 4.48 | + | 5.09E-01 | 1 | 4.85E-01 | 1 | 0.05 | N |
| 219 | SART3 | 4.17 | + | 5.12E-01 | 1 | 9.76E-02 | 1 | 1.66 | Y |
| 220 | YTHDF1 | 4.65 | + | 5.13E-01 | 1 | 2.90E-04 | 1.45E-01 | 7.48 | N |
| 221 | SNAP29 | 4.79 | + | 5.13E-01 | 1 | 3.66E-03 | 1 | 4.94 | N |
| 222 | PPP2R5C | 4.37 | + | 5.14E-01 | 1 | 1.81E-01 | 1 | 1.04 | N |
| 223 | C11orf46 | 4.20 | + | 5.15E-01 | 1 | 1.84E-02 | 1 | 3.34 | N |
| 224 | HBS1L | 4.18 | + | 5.19E-01 | 1 | 7.25E-02 | 1 | 1.97 | N |
| 225 | ZSCAN5A | 4.11 | + | 5.20E-01 | 1 | 4.37E-01 | 1 | 0.17 | N |
| 226 | MON1B | 4.34 | + | 5.21E-01 | 1 | 4.71E-01 | 1 | 0.10 | N |
| 227 | TBRG1 | 4.12 | - | 5.21E-01 | 1 | 1.81E-04 | 9.05E-02 | 7.97 | N |
| 228 | OCIAD1 | 4.43 | + | 5.23E-01 | 1 | 3.84E-01 | 1 | 0.31 | N |
| 229 | BAP1 | 4.38 | + | 5.23E-01 | 1 | 4.16E-01 | 1 | 0.23 | N |
| 230 | PWP1 | 4.27 | + | 5.32E-01 | 1 | 9.88E-01 | 1 | -0.62 | N |
| 231 | UBAP2L | 4.26 | + | 5.33E-01 | 1 | 6.87E-02 | 1 | 2.05 | N |
| 232 | KIAA0174 | 6.20 | + | 5.33E-01 | 1 | 4.01E-01 | 1 | 0.28 | N |
| 233 | CSRP2BP | 4.31 | + | 5.37E-01 | 1 | 2.17E-03 | 1 | 5.51 | N |
| 234 | TERF2IP | 4.79 | - | 5.37E-01 | 1 | 1.45E-03 | 7.24E-01 | 5.92 | N |
| 235 | STK24 | 4.89 | + | 5.37E-01 | 1 | 1.27E-01 | 1 | 1.44 | N |
| 236 | PUM2 | 4.26 | + | 5.41E-01 | 1 | 4.84E-03 | 1 | 4.72 | N |
| 237 | U2AF2 | 4.41 | = | 5.42E-01 | 1 | 1.05E-05 | 5.25E-03 | 10.85 | N |
| 238 | PRDM4 | 4.34 | + | 5.44E-01 | 1 | 2.27E-01 | 1 | 0.87 | N |
| 239 | TATDN2 | 4.24 | + | 5.52E-01 | 1 | 6.97E-01 | 1 | -0.23 | N |
| 240 | WBP11 | 4.48 | + | 5.54E-01 | 1 | 1.91E-01 | 1 | 1.06 | N |
| 241 | GOLPH3 | 4.33 | + | 5.59E-01 | 1 | 7.91E-01 | 1 | -0.35 | N |
| 242 | RNF4 | 4.14 | - | 5.61E-01 | 1 | 3.05E-01 | 1 | 0.61 | N |
| 243 | ATL2 | 4.19 | + | 5.61E-01 | 1 | 6.65E-02 | 1 | 2.13 | Y |
| 244 | UPF1 | 4.55 | + | 5.62E-01 | 1 | 1.43E-01 | 1 | 1.37 | N |
| 245 | INTS10 | 4.14 | + | 5.62E-01 | 1 | 7.10E-04 | 3.55E-01 | 6.67 | N |
| 246 | C3orf19 | 4.24 | + | 5.64E-01 | 1 | 1.35E-02 | 1 | 3.73 | N |
| 247 | SIRT5 | 4.43 | + | 5.65E-01 | 1 | 6.53E-01 | 1 | -0.14 | N |
| 248 | RALGAPB | 4.37 | + | 5.70E-01 | 1 | 3.15E-01 | 1 | 0.59 | N |
| 249 | RELA | 4.34 | - | 5.71E-01 | 1 | 1.39E-03 | 6.97E-01 | 6.01 | N |
| 250 | SF3B2 | 5.57 | + | 5.72E-01 | 1 | 5.04E-01 | 1 | 0.13 | N |
| 251 | BUD13 | 4.54 | + | 5.73E-01 | 1 | 4.23E-01 | 1 | 0.30 | N |
| 252 | LSM14B | 4.18 | + | 5.77E-01 | 1 | 2.10E-01 | 1 | 1.01 | N |
| 253 | DPF2 | 4.99 | + | 5.77E-01 | 1 | 3.44E-01 | 1 | 0.52 | N |
| 254 | USP4 | 4.73 | + | 5.77E-01 | 1 | 1.81E-01 | 1 | 1.16 | N |
| 255 | MRFAP1L1 | 4.61 | + | 5.85E-01 | 1 | 3.62E-01 | 1 | 0.48 | N |
| 256 | GSK3A | 4.44 | = | 5.88E-01 | 1 | 1.82E-02 | 1 | 3.48 | Y |
| 257 | HARS2 | 4.24 | + | 5.89E-01 | 1 | 2.92E-02 | 1 | 3.01 | N |
| 258 | SPOP | 4.50 | + | 5.90E-01 | 1 | 4.36E-01 | 1 | 0.30 | N |
| 259 | RNF26 | 4.21 | + | 5.90E-01 | 1 | 7.51E-01 | 1 | -0.24 | N |
| 260 | MAP3K7 | 4.31 | + | 5.91E-01 | 1 | 3.64E-01 | 1 | 0.48 | Y |
| 261 | QRSL1 | 4.40 | + | 5.91E-01 | 1 | 8.80E-01 | 1 | -0.40 | N |
| 262 | GAK | 4.12 | + | 5.93E-01 | 1 | 2.23E-02 | 1 | 3.28 | N |
| 263 | EDC3 | 4.18 | + | 5.93E-01 | 1 | 1.93E-01 | 1 | 1.12 | N |
| 264 | TFIP11 | 4.69 | - | 5.96E-01 | 1 | 3.46E-01 | 1 | 0.54 | N |
| 265 | FAM32A | 4.15 | = | 5.96E-01 | 1 | 1.29E-04 | 6.47E-02 | 8.44 | N |
| 266 | TEX261 | 4.31 | + | 5.98E-01 | 1 | 9.63E-01 | 1 | -0.48 | N |
| 267 | ARHGEF18 | 4.20 | + | 5.99E-01 | 1 | 8.85E-01 | 1 | -0.39 | N |
| 268 | USP19 | 4.52 | + | 6.05E-01 | 1 | 3.79E-02 | 1 | 2.77 | N |
| 269 | NAP1L4 | 4.24 | + | 6.07E-01 | 1 | 2.82E-05 | 1.41E-02 | 9.97 | N |
| 270 | FAM192A | 5.10 | + | 6.07E-01 | 1 | 1.83E-04 | 9.15E-02 | 8.11 | N |
| 271 | UTP3 | 4.14 | + | 6.08E-01 | 1 | 1.48E-01 | 1 | 1.41 | N |
| 272 | DDB1 | 4.92 | + | 6.10E-01 | 1 | 3.97E-03 | 1 | 5.04 | N |
| 273 | MRPL35 | 4.28 | + | 6.15E-01 | 1 | 4.75E-01 | 1 | 0.26 | N |
| 274 | RABGEF1 | 4.45 | - | 6.15E-01 | 1 | 2.49E-02 | 1 | 3.21 | N |
| 275 | C6orf64 | 5.18 | + | 6.15E-01 | 1 | 8.73E-01 | 1 | -0.35 | N |
| 276 | RPAP1 | 4.48 | + | 6.16E-01 | 1 | 6.07E-01 | 1 | 0.01 | N |
| 277 | IDH3B | 4.40 | + | 6.17E-01 | 1 | 8.99E-01 | 1 | -0.38 | N |
| 278 | VPS8 | 4.47 | + | 6.19E-01 | 1 | 1.08E-02 | 1 | 4.05 | N |
| 279 | PANK4 | 4.49 | + | 6.22E-01 | 1 | 9.71E-01 | 1 | -0.45 | N |
| 280 | VPS52 | 4.90 | + | 6.22E-01 | 1 | 3.99E-02 | 1 | 2.75 | N |
| 281 | DIDO1 | 4.27 | + | 6.24E-01 | 1 | 5.21E-01 | 1 | 0.18 | Y |
| 282 | YWHAE | 4.51 | + | 6.25E-01 | 1 | 2.15E-01 | 1 | 1.06 | N |
| 283 | ELF2 | 4.11 | + | 6.25E-01 | 1 | 4.20E-01 | 1 | 0.40 | N |
| 284 | ZFP64 | 4.32 | + | 6.25E-01 | 1 | 6.99E-02 | 1 | 2.19 | N |
| 285 | UBE3B | 4.36 | + | 6.37E-01 | 1 | 8.57E-01 | 1 | -0.30 | N |
| 286 | BRAP | 5.50 | + | 6.40E-01 | 1 | 1.63E-01 | 1 | 1.37 | N |
| 287 | FXR2 | 4.88 | + | 6.43E-01 | 1 | 6.89E-01 | 1 | -0.07 | N |
| 288 | MCRS1 | 4.11 | - | 6.44E-01 | 1 | 7.68E-06 | 3.84E-03 | 11.34 | N |
| 289 | PPHLN1 | 4.95 | + | 6.44E-01 | 1 | 4.46E-05 | 2.23E-02 | 9.58 | N |
| 290 | PACRGL | 4.25 | + | 6.46E-01 | 1 | 3.33E-01 | 1 | 0.66 | N |
| 291 | DDX19A | 4.54 | + | 6.51E-01 | 1 | 9.78E-01 | 1 | -0.41 | N |
| 292 | XRCC6 | 4.84 | + | 6.54E-01 | 1 | 7.98E-01 | 1 | -0.20 | Y |
| 293 | UBOX5 | 4.55 | + | 6.54E-01 | 1 | 1.13E-01 | 1 | 1.76 | N |
| 294 | USP28 | 4.47 | + | 6.54E-01 | 1 | 9.40E-01 | 1 | -0.36 | N |
| 295 | TBP | 4.43 | + | 6.56E-01 | 1 | 2.43E-03 | 1 | 5.60 | N |
| 296 | EIF2AK1 | 4.50 | - | 6.59E-01 | 1 | 1.97E-02 | 1 | 3.51 | N |
| 297 | RNF167 | 4.27 | + | 6.59E-01 | 1 | 3.59E-01 | 1 | 0.61 | N |
| 298 | MESDC2 | 4.17 | + | 6.62E-01 | 1 | 5.65E-01 | 1 | 0.16 | N |
| 299 | ATMIN | 4.48 | + | 6.64E-01 | 1 | 1.86E-01 | 1 | 1.27 | N |
| 300 | C18orf55 | 4.26 | + | 6.64E-01 | 1 | 7.37E-02 | 1 | 2.20 | N |
| 301 | NOL10 | 4.39 | + | 6.64E-01 | 1 | 1.20E-02 | 1 | 4.01 | N |
| 302 | CUL1 | 4.27 | + | 6.65E-01 | 1 | 5.85E-01 | 1 | 0.13 | N |
| 303 | NSFL1C | 4.29 | = | 6.66E-01 | 1 | 1.64E-01 | 1 | 1.40 | N |
| 304 | MLH1 | 4.68 | + | 6.69E-01 | 1 | 2.06E-01 | 1 | 1.18 | Y |
| 305 | SENP3 | 4.29 | + | 6.69E-01 | 1 | 2.75E-01 | 1 | 0.89 | N |
| 306 | DCTN5 | 4.19 | + | 6.70E-01 | 1 | 9.93E-02 | 1 | 1.91 | N |
| 307 | MGC2752 | 4.30 | + | 6.72E-01 | 1 | 7.01E-01 | 1 | -0.04 | N |
| 308 | STIM1 | 4.13 | + | 6.77E-01 | 1 | 9.72E-02 | 1 | 1.94 | N |
| 309 | BAT3 | 4.33 | + | 6.79E-01 | 1 | 3.25E-01 | 1 | 0.74 | N |
| 310 | ABCF3 | 4.19 | + | 6.79E-01 | 1 | 3.05E-02 | 1 | 3.10 | N |
| 311 | VPS39 | 4.84 | + | 6.81E-01 | 1 | 4.85E-03 | 1 | 4.95 | N |
| 312 | SNX14 | 4.58 | + | 6.81E-01 | 1 | 5.30E-01 | 1 | 0.25 | N |
| 313 | MTIF2 | 4.14 | + | 6.83E-01 | 1 | 3.68E-01 | 1 | 0.62 | N |
| 314 | MED24 | 4.19 | + | 6.86E-01 | 1 | 6.46E-04 | 3.23E-01 | 6.97 | N |
| 315 | MTPAP | 4.68 | + | 6.86E-01 | 1 | 8.15E-01 | 1 | -0.17 | N |
| 316 | MRFAP1 | 4.71 | + | 6.86E-01 | 1 | 4.21E-03 | 1 | 5.09 | N |
| 317 | KIAA0317 | 4.14 | + | 6.87E-01 | 1 | 3.36E-01 | 1 | 0.72 | N |
| 318 | UNC45A | 4.42 | + | 6.92E-01 | 1 | 6.63E-01 | 1 | 0.04 | N |
| 319 | ATPAF1 | 4.26 | + | 6.92E-01 | 1 | 2.10E-01 | 1 | 1.19 | N |
| 320 | TBC1D20 | 4.28 | + | 6.95E-01 | 1 | 2.67E-04 | 1.34E-01 | 7.86 | N |
| 321 | BRD7 | 5.61 | + | 6.95E-01 | 1 | 9.99E-01 | 1 | -0.36 | N |
| 322 | SMAD2 | 4.17 | + | 6.99E-01 | 1 | 4.06E-03 | 1 | 5.15 | N |
| 323 | NT5C2 | 4.18 | + | 7.03E-01 | 1 | 4.35E-02 | 1 | 2.78 | N |
| 324 | LOC285033 | 4.15 | + | 7.03E-01 | 1 | 1.85E-01 | 1 | 1.34 | N |
| 325 | SUPV3L1 | 4.55 | + | 7.03E-01 | 1 | 2.27E-03 | 1 | 5.73 | N |
| 326 | ZSWIM3 | 4.22 | + | 7.05E-01 | 1 | 3.50E-02 | 1 | 3.00 | N |
| 327 | DCTN2 | 4.25 | = | 7.10E-01 | 1 | 5.52E-04 | 2.76E-01 | 7.16 | N |
| 328 | VPRBP | 4.32 | + | 7.11E-01 | 1 | 3.32E-01 | 1 | 0.76 | N |
| 329 | NUP43 | 4.29 | + | 7.11E-01 | 1 | 1.20E-01 | 1 | 1.78 | N |
| 330 | ATXN10 | 4.80 | + | 7.14E-01 | 1 | 8.17E-01 | 1 | -0.13 | N |
| 331 | CHERP | 4.59 | + | 7.16E-01 | 1 | 8.74E-04 | 4.37E-01 | 6.71 | N |
| 332 | TARDBP | 4.33 | + | 7.16E-01 | 1 | 1.45E-01 | 1 | 1.60 | N |
| 333 | RAB28 | 4.23 | + | 7.17E-01 | 1 | 3.87E-01 | 1 | 0.62 | N |
| 334 | SAPS2 | 4.16 | + | 7.18E-01 | 1 | 4.23E-02 | 1 | 2.83 | N |
| 335 | AKT2 | 4.36 | + | 7.20E-01 | 1 | 1.28E-03 | 0.640212 | 6.33 | N |
| 336 | HNRNPUL1 | 4.17 | + | 7.20E-01 | 1 | 8.67E-01 | 1 | -0.19 | N |
| 337 | PPIL2 | 4.13 | + | 7.25E-01 | 1 | 4.82E-04 | 2.41E-01 | 7.31 | N |
| 338 | ZNF3 | 4.29 | + | 7.27E-01 | 1 | 2.32E-01 | 1 | 1.14 | Y |
| 339 | PPP1R11 | 4.28 | + | 7.27E-01 | 1 | 7.16E-01 | 1 | 0.02 | N |
| 340 | ZFAND3 | 4.97 | + | 7.29E-01 | 1 | 3.19E-01 | 1 | 0.83 | N |
| 341 | RSRC2 | 4.22 | + | 7.29E-01 | 1 | 3.54E-03 | 1 | 5.33 | N |
| 342 | GORASP1 | 4.59 | + | 7.31E-01 | 1 | 9.35E-04 | 4.67E-01 | 6.66 | N |
| 343 | SRPR | 4.24 | + | 7.31E-01 | 1 | 1.35E-02 | 1 | 3.99 | N |
| 344 | ENOX2 | 4.14 | + | 7.34E-01 | 1 | 4.30E-01 | 1 | 0.54 | N |
| 345 | NGLY1 | 4.26 | + | 7.35E-01 | 1 | 8.30E-01 | 1 | -0.12 | N |
| 346 | PCID2 | 4.20 | + | 7.38E-01 | 1 | 4.25E-03 | 1 | 5.16 | N |
| 347 | ZNF576 | 4.22 | + | 7.40E-01 | 1 | 9.60E-02 | 1 | 2.04 | N |
| 348 | MARK2 | 4.68 | + | 7.44E-01 | 1 | 1.58E-01 | 1 | 1.55 | N |
| 349 | CHCHD4 | 4.22 | + | 7.45E-01 | 1 | 9.18E-01 | 1 | -0.21 | N |
| 350 | OSBPL2 | 4.75 | + | 7.46E-01 | 1 | 3.14E-01 | 1 | 0.87 | N |
| 351 | TH1L | 4.39 | + | 7.49E-01 | 1 | 2.97E-01 | 1 | 0.92 | N |
| 352 | SEC24C | 4.41 | + | 7.49E-01 | 1 | 1.14E-02 | 1 | 4.18 | N |
| 353 | MRPS5 | 4.90 | = | 7.54E-01 | 1 | 8.01E-02 | 1 | 2.24 | N |
| 354 | NCOA5 | 4.35 | + | 7.56E-01 | 1 | 6.05E-01 | 1 | 0.22 | N |
| 355 | DCTD | 4.90 | + | 7.59E-01 | 1 | 6.13E-02 | 1 | 2.52 | N |
| 356 | CHP | 4.23 | + | 7.63E-01 | 1 | 2.25E-01 | 1 | 1.22 | N |
| 357 | ECD | 4.23 | + | 7.64E-01 | 1 | 1.78E-01 | 1 | 1.46 | N |
| 358 | TNPO2 | 4.15 | + | 7.65E-01 | 1 | 9.86E-02 | 1 | 2.05 | N |
| 359 | SFT2D1 | 4.23 | + | 7.66E-01 | 1 | 4.26E-02 | 1 | 2.89 | N |
| 360 | NDUFS2 | 4.24 | + | 7.68E-01 | 1 | 4.18E-01 | 1 | 0.61 | N |
| 361 | UBTF | 4.42 | + | 7.68E-01 | 1 | 1.56E-01 | 1 | 1.60 | N |
| 362 | TMEM199 | 4.60 | + | 7.71E-01 | 1 | 7.05E-01 | 1 | 0.09 | N |
| 363 | CHMP7 | 4.18 | + | 7.71E-01 | 1 | 5.53E-01 | 1 | 0.33 | N |
| 364 | SLC25A17 | 4.78 | + | 7.71E-01 | 1 | 3.74E-01 | 1 | 0.72 | N |
| 365 | APTX | 4.17 | + | 7.72E-01 | 1 | 9.78E-02 | 1 | 2.07 | N |
| 366 | PTPN23 | 4.22 | + | 7.72E-01 | 1 | 5.44E-02 | 1 | 2.65 | N |
| 367 | FTSJ2 | 4.30 | + | 7.74E-01 | 1 | 2.65E-01 | 1 | 1.07 | N |
| 368 | DNAJC14 | 4.84 | + | 7.74E-01 | 1 | 1.35E-01 | 1 | 1.74 | N |
| 369 | ZNHIT3 | 4.15 | + | 7.76E-01 | 1 | 3.36E-02 | 1 | 3.14 | N |
| 370 | EXOC7 | 4.22 | + | 7.77E-01 | 1 | 8.38E-02 | 1 | 2.23 | N |
| 371 | SYS1 | 4.12 | + | 7.78E-01 | 1 | 5.55E-01 | 1 | 0.34 | N |
| 372 | POLR3F | 4.23 | + | 7.79E-01 | 1 | 1.99E-01 | 1 | 1.37 | N |
| 373 | EPS15L1 | 4.12 | + | 7.80E-01 | 1 | 7.96E-01 | 1 | -0.02 | N |
| 374 | PRKAG1 | 4.30 | + | 7.80E-01 | 1 | 8.81E-01 | 1 | -0.12 | N |
| 375 | WDR45L | 5.45 | + | 7.80E-01 | 1 | 9.28E-01 | 1 | -0.17 | N |
| 376 | MTX2 | 4.42 | + | 7.81E-01 | 1 | 8.63E-03 | 1 | 4.51 | N |
| 377 | EIF2B5 | 4.77 | + | 7.84E-01 | 1 | 2.99E-04 | 1.50E-01 | 7.87 | N |
| 378 | ZDHHC13 | 4.21 | + | 7.84E-01 | 1 | 1.90E-01 | 1 | 1.42 | N |
| 379 | USP47 | 4.35 | + | 7.85E-01 | 1 | 9.99E-02 | 1 | 2.06 | N |
| 380 | COX18 | 4.13 | + | 7.88E-01 | 1 | 2.73E-02 | 1 | 3.36 | N |
| 381 | GNPNAT1 | 4.17 | + | 7.89E-01 | 1 | 1.03E-01 | 1 | 2.04 | N |
| 382 | ASB3 | 4.22 | + | 7.89E-01 | 1 | 2.65E-02 | 1 | 3.39 | N |
| 383 | THUMPD3 | 4.41 | + | 7.91E-01 | 1 | 3.72E-01 | 1 | 0.76 | N |
| 384 | NUP88 | 4.69 | + | 7.92E-01 | 1 | 3.09E-01 | 1 | 0.94 | N |
| 385 | TAF4 | 4.25 | + | 8.00E-01 | 1 | 1.36E-04 | 6.81E-02 | 8.68 | N |
| 386 | RTN3 | 4.45 | + | 8.00E-01 | 1 | 2.46E-01 | 1 | 1.18 | N |
| 387 | ARFIP1 | 4.19 | + | 8.02E-01 | 1 | 2.48E-03 | 1 | 5.78 | N |
| 388 | OPA1 | 4.24 | + | 8.11E-01 | 1 | 7.67E-02 | 1 | 2.36 | N |
| 389 | BMS1 | 4.39 | + | 8.12E-01 | 1 | 3.64E-03 | 1 | 5.41 | N |
| 390 | MAPKAP1 | 4.79 | + | 8.14E-01 | 1 | 7.37E-01 | 1 | 0.10 | N |
| 391 | SS18 | 4.54 | + | 8.15E-01 | 1 | 7.11E-02 | 1 | 2.44 | N |
| 392 | ZDHHC5 | 4.96 | + | 8.15E-01 | 1 | 9.23E-01 | 1 | -0.12 | N |
| 393 | NIF3L1 | 4.42 | + | 8.16E-01 | 1 | 2.05E-01 | 1 | 1.38 | N |
| 394 | PPP4R1 | 4.13 | + | 8.17E-01 | 1 | 4.84E-01 | 1 | 0.52 | N |
| 395 | IMMT | 4.90 | + | 8.18E-01 | 1 | 8.74E-01 | 1 | -0.07 | N |
| 396 | ZNF207 | 5.32 | + | 8.20E-01 | 1 | 1.29E-05 | 6.45E-03 | 11.06 | N |
| 397 | PDHX | 4.21 | + | 8.22E-01 | 1 | 7.08E-01 | 1 | 0.15 | N |
| 398 | VPS37C | 4.31 | + | 8.22E-01 | 1 | 5.08E-01 | 1 | 0.48 | N |
| 399 | SF3A3 | 4.20 | + | 8.24E-01 | 1 | 9.85E-01 | 1 | -0.18 | N |
| 400 | VCP | 4.35 | + | 8.25E-01 | 1 | 2.04E-01 | 1 | 1.40 | N |
| 401 | THEM4 | 4.59 | + | 8.25E-01 | 1 | 3.92E-02 | 1 | 3.05 | Y |
| 402 | HKR1 | 4.17 | + | 8.26E-01 | 1 | 3.90E-02 | 1 | 3.05 | N |
| 403 | PTPRA | 5.48 | + | 8.27E-01 | 1 | 1.45E-01 | 1 | 1.74 | N |
| 404 | RAB5A | 4.15 | + | 8.29E-01 | 1 | 4.10E-01 | 1 | 0.70 | N |
| 405 | SCO1 | 4.38 | + | 8.33E-01 | 1 | 4.08E-01 | 1 | 0.71 | N |
| 406 | CSNK2A1 | 4.36 | + | 8.33E-01 | 1 | 6.09E-01 | 1 | 0.31 | N |
| 407 | RAF1 | 4.93 | + | 8.37E-01 | 1 | 2.23E-03 | 1 | 5.93 | Y |
| 408 | EIF1AD | 4.63 | + | 8.37E-01 | 1 | 3.80E-03 | 1 | 5.39 | N |
| 409 | FAM20B | 4.26 | + | 8.38E-01 | 1 | 3.78E-01 | 1 | 0.80 | N |
| 410 | MTMR1 | 4.12 | + | 8.42E-01 | 1 | 2.32E-01 | 1 | 1.29 | N |
| 411 | UNC50 | 4.69 | + | 8.43E-01 | 1 | 9.34E-01 | 1 | -0.10 | N |
| 412 | DDX52 | 4.44 | + | 8.43E-01 | 1 | 7.28E-01 | 1 | 0.15 | N |
| 413 | RAB5B | 6.00 | + | 8.44E-01 | 1 | 2.90E-01 | 1 | 1.07 | N |
| 414 | NAIF1 | 4.66 | + | 8.45E-01 | 1 | 6.92E-01 | 1 | 0.20 | N |
| 415 | C2CD3 | 4.11 | + | 8.46E-01 | 1 | 1.22E-02 | 1 | 4.24 | N |
| 416 | C22orf39 | 5.23 | + | 8.48E-01 | 1 | 2.23E-01 | 1 | 1.34 | N |
| 417 | PTBP1 | 4.23 | + | 8.49E-01 | 1 | 6.46E-04 | 3.23E-01 | 7.18 | N |
| 418 | C10orf28 | 4.41 | + | 8.51E-01 | 1 | 5.51E-01 | 1 | 0.43 | N |
| 419 | NUDT15 | 4.19 | + | 8.54E-01 | 1 | 7.21E-02 | 1 | 2.47 | N |
| 420 | FBXO18 | 4.42 | + | 8.54E-01 | 1 | 1.47E-05 | 7.34E-03 | 10.97 | N |
| 421 | PRPF18 | 5.10 | + | 8.56E-01 | 1 | 6.05E-01 | 1 | 0.35 | N |
| 422 | NUDT9 | 4.24 | + | 8.57E-01 | 1 | 8.83E-01 | 1 | -0.03 | N |
| 423 | MAPKAPK5 | 4.93 | + | 8.62E-01 | 1 | 6.44E-02 | 1 | 2.59 | Y |
| 424 | RANBP9 | 4.82 | + | 8.62E-01 | 1 | 2.80E-01 | 1 | 1.13 | N |
| 425 | PTCD3 | 4.55 | + | 8.63E-01 | 1 | 1.33E-04 | 6.65E-02 | 8.78 | N |
| 426 | SMARCE1 | 4.43 | + | 8.64E-01 | 1 | 2.47E-02 | 1 | 3.55 | N |
| 427 | FTSJD2 | 4.58 | + | 8.64E-01 | 1 | 5.86E-02 | 1 | 2.69 | N |
| 428 | ARAP1 | 4.24 | + | 8.64E-01 | 1 | 9.25E-01 | 1 | -0.07 | N |
| 429 | SMURF1 | 4.30 | + | 8.65E-01 | 1 | 5.51E-02 | 1 | 2.75 | N |
| 430 | LYRM2 | 4.28 | + | 8.67E-01 | 1 | 7.30E-01 | 1 | 0.17 | N |
| 431 | CTNS | 4.24 | + | 8.68E-01 | 1 | 6.60E-01 | 1 | 0.27 | N |
| 432 | ZNF434 | 5.02 | + | 8.70E-01 | 1 | 3.18E-03 | 1 | 5.61 | N |
| 433 | C3orf17 | 4.28 | + | 8.73E-01 | 1 | 4.38E-01 | 1 | 0.69 | N |
| 434 | TMEM127 | 5.26 | + | 8.73E-01 | 1 | 3.01E-02 | 1 | 3.37 | N |
| 435 | MKRN1 | 4.44 | + | 8.77E-01 | 1 | 2.18E-01 | 1 | 1.39 | N |
| 436 | KIAA1967 | 4.48 | + | 8.79E-01 | 1 | 3.95E-01 | 1 | 0.80 | N |
| 437 | FIG4 | 4.56 | + | 8.80E-01 | 1 | 3.22E-01 | 1 | 1.00 | N |
| 438 | AP3D1 | 4.33 | + | 8.81E-01 | 1 | 8.54E-01 | 1 | 0.03 | N |
| 439 | CRKL | 4.12 | + | 8.82E-01 | 1 | 7.74E-01 | 1 | 0.13 | N |
| 440 | ZNF197 | 4.16 | + | 8.84E-01 | 1 | 6.09E-01 | 1 | 0.37 | N |
| 441 | USP7 | 5.32 | + | 8.85E-01 | 1 | 4.00E-01 | 1 | 0.79 | N |
| 442 | DHX30 | 4.51 | + | 8.90E-01 | 1 | 2.22E-02 | 1 | 3.69 | N |
| 443 | C16orf88 | 4.19 | + | 8.90E-01 | 1 | 3.52E-02 | 1 | 3.23 | N |
| 444 | COG1 | 4.19 | + | 8.92E-01 | 1 | 2.97E-03 | 1 | 5.71 | N |
| 445 | C15orf44 | 5.51 | + | 8.92E-01 | 1 | 1.66E-01 | 1 | 1.68 | N |
| 446 | PDCD7 | 5.33 | + | 8.94E-01 | 1 | 3.67E-03 | 1 | 5.50 | Y |
| 447 | SNAPC5 | 4.48 | + | 8.95E-01 | 1 | 2.18E-01 | 1 | 1.41 | N |
| 448 | SPPL3 | 4.86 | + | 8.97E-01 | 1 | 1.92E-02 | 1 | 3.84 | N |
| 449 | DHX35 | 4.21 | + | 9.02E-01 | 1 | 2.08E-03 | 1 | 6.07 | N |
| 450 | CYB5B | 4.75 | + | 9.03E-01 | 1 | 9.24E-01 | 1 | -0.02 | N |
| 451 | EXOSC9 | 4.21 | + | 9.05E-01 | 1 | 2.81E-03 | 1 | 5.77 | N |
| 452 | ZNF271 | 4.76 | + | 9.10E-01 | 1 | 4.86E-01 | 1 | 0.63 | N |
| 453 | RNF8 | 4.70 | + | 9.11E-01 | 1 | 7.92E-01 | 1 | 0.14 | Y |
| 454 | SFRS1 | 4.28 | + | 9.12E-01 | 1 | 2.03E-02 | 1 | 3.81 | N |
| 455 | RBM45 | 5.26 | + | 9.14E-01 | 1 | 3.56E-03 | 1 | 5.55 | N |
| 456 | RNASEN | 4.29 | + | 9.14E-01 | 1 | 7.17E-01 | 1 | 0.24 | N |
| 457 | CUL2 | 4.16 | + | 9.17E-01 | 1 | 8.68E-01 | 1 | 0.05 | N |
| 458 | NOL7 | 4.51 | + | 9.19E-01 | 1 | 6.17E-02 | 1 | 2.70 | N |
| 459 | GTPBP1 | 4.55 | + | 9.21E-01 | 1 | 7.19E-03 | 1 | 4.85 | Y |
| 460 | SLTM | 4.27 | + | 9.22E-01 | 1 | 3.10E-01 | 1 | 1.09 | N |
| 461 | RNF123 | 4.35 | + | 9.22E-01 | 1 | 6.18E-02 | 1 | 2.70 | N |
| 462 | RTF1 | 4.94 | + | 9.23E-01 | 1 | 4.93E-01 | 1 | 0.63 | N |
| 463 | ANP32A | 5.14 | + | 9.24E-01 | 1 | 4.75E-01 | 1 | 0.66 | N |
| 464 | LSG1 | 4.44 | + | 9.24E-01 | 1 | 1.27E-02 | 1 | 4.29 | N |
| 465 | NUDT3 | 4.12 | + | 9.27E-01 | 1 | 1.02E-01 | 1 | 2.21 | N |
| 466 | ATG3 | 4.21 | + | 9.29E-01 | 1 | 8.14E-01 | 1 | 0.13 | N |
| 467 | FAM125B | 4.17 | + | 9.32E-01 | 1 | 4.29E-01 | 1 | 0.77 | N |
| 468 | KPNA1 | 4.26 | + | 9.35E-01 | 1 | 2.17E-01 | 1 | 1.46 | Y |
| 469 | NRF1 | 4.41 | + | 9.36E-01 | 1 | 2.79E-02 | 1 | 3.51 | N |
| 470 | ANAPC10 | 4.14 | + | 9.36E-01 | 1 | 4.64E-01 | 1 | 0.70 | N |
| 471 | PAPOLA | 4.23 | + | 9.38E-01 | 1 | 3.49E-01 | 1 | 0.99 | N |
| 472 | SDAD1 | 4.93 | + | 9.40E-01 | 1 | 8.41E-01 | 1 | 0.11 | N |
| 473 | SMARCB1 | 4.25 | + | 9.44E-01 | 1 | 5.67E-01 | 1 | 0.51 | N |
| 474 | C20orf4 | 4.91 | + | 9.45E-01 | 1 | 5.08E-02 | 1 | 2.92 | N |
| 475 | ADIPOR2 | 4.19 | + | 9.46E-01 | 1 | 4.59E-01 | 1 | 0.72 | N |
| 476 | GTF3C2 | 5.15 | + | 9.46E-01 | 1 | 3.05E-02 | 1 | 3.43 | N |
| 477 | C16orf80 | 4.13 | + | 9.48E-01 | 1 | 4.49E-01 | 1 | 0.75 | N |
| 478 | CNOT10 | 4.43 | + | 9.49E-01 | 1 | 8.33E-01 | 1 | 0.13 | N |
| 479 | RAB7A | 4.51 | + | 9.49E-01 | 1 | 6.77E-01 | 1 | 0.34 | N |
| 480 | TSPAN3 | 4.28 | + | 9.50E-01 | 1 | 9.04E-01 | 1 | 0.05 | N |
| 481 | RUNDC1 | 4.32 | + | 9.50E-01 | 1 | 5.43E-01 | 1 | 0.56 | N |
| 482 | COIL | 4.56 | + | 9.51E-01 | 1 | 1.35E-01 | 1 | 1.95 | N |
| 483 | UBIAD1 | 4.14 | + | 9.57E-01 | 1 | 9.42E-02 | 1 | 2.32 | N |
| 484 | TLK2 | 6.07 | + | 9.62E-01 | 1 | 5.01E-01 | 1 | 0.65 | N |
| 485 | TCEB3 | 4.18 | + | 9.64E-01 | 1 | 9.40E-01 | 1 | 0.03 | N |
| 486 | CARKD | 4.80 | + | 9.68E-01 | 1 | 6.18E-03 | 1 | 5.05 | N |
| 487 | ZNF74 | 4.30 | + | 9.69E-01 | 1 | 1.48E-03 | 7.42E-01 | 6.48 | N |
| 488 | ALG9 | 4.71 | + | 9.69E-01 | 1 | 3.02E-01 | 1 | 1.17 | N |
| 489 | PPP5C | 4.75 | + | 9.69E-01 | 1 | 4.49E-02 | 1 | 3.07 | N |
| 490 | EIF4ENIF1 | 4.69 | + | 9.70E-01 | 1 | 6.02E-01 | 1 | 0.48 | N |
| 491 | CIAO1 | 5.73 | + | 9.72E-01 | 1 | 2.70E-03 | 1 | 5.89 | N |
| 492 | MED28 | 4.25 | + | 9.73E-01 | 1 | 1.10E-01 | 1 | 2.18 | N |
| 493 | LARP4B | 4.58 | + | 9.78E-01 | 1 | 1.29E-02 | 1 | 4.33 | N |
| 494 | INTS9 | 4.85 | + | 9.79E-01 | 1 | 8.55E-01 | 1 | 0.14 | N |
| 495 | C7orf26 | 4.18 | + | 9.82E-01 | 1 | 9.36E-03 | 1 | 4.65 | N |
| 496 | L3MBTL2 | 4.34 | + | 9.83E-01 | 1 | 1.50E-01 | 1 | 1.88 | N |
| 497 | TRIM68 | 4.28 | + | 9.85E-01 | 1 | 7.82E-01 | 1 | 0.23 | N |
| 498 | NUDCD3 | 4.14 | + | 9.92E-01 | 1 | 9.32E-01 | 1 | 0.06 | N |
| 499 | FAF1 | 4.40 | + | 9.98E-01 | 1 | 9.63E-01 | 1 | 0.04 | N |
| 500 | WRNIP1 | 4.21 | + | 9.99E-01 | 1 | 6.92E-01 | 1 | 0.37 | N |

*Values in the column “Survival direction”: "-" = more survivors in the low-expression third. "+" = more survivors in the higher-expression third.

**Gray background – genes that are associated with the Immune system.

| **Supplementary table 4: Survival analysis results for random CVR genes group** | | | | | | | |  |  |
| --- | --- | --- | --- | --- | --- | --- | --- | --- | --- |
|  |  |  |  | **Survival (Logrank test)** | | | | **log norm** |  |
|  |  |  |  | **Normalized** | | **Un-normalized** | | **/ raw** |  |
| **#** | **Gene Name** | **CVR** | **Survival direction*** | **Nominal** | **Bonferroni correction** | **Nominal** | **Bonferroni correction** | **nominal logrank** | **Immune response**** |
| 1 | MIA2 | 1.20 | + | 9.44E-09 | 4.72E-06 | 5.47E-10 | 2.73E-07 | 2.85 | N |
| 2 | LAT | 1.47 | - | 1.15E-08 | 5.74E-06 | 6.79E-06 | 3.39E-03 | -6.38 | Y |
| 3 | C9orf109 | 1.57 | - | 4.82E-07 | 2.41E-04 | 1.62E-07 | 8.10E-05 | 1.09 | N |
| 4 | RAG1 | 1.68 | + | 4.93E-07 | 2.47E-04 | 1.11E-07 | 5.53E-05 | 1.50 | Y |
| 5 | TRPM3 | 1.32 | + | 1.11E-06 | 5.57E-04 | 2.08E-07 | 1.04E-04 | 1.68 | N |
| 6 | SLPI | 1.22 | - | 1.33E-06 | 6.65E-04 | 3.32E-07 | 1.66E-04 | 1.39 | Y |
| 7 | KCTD16 | 1.19 | + | 2.87E-06 | 1.43E-03 | 2.07E-06 | 1.03E-03 | 0.33 | N |
| 8 | GK3P | 1.93 | + | 6.76E-06 | 3.38E-03 | 3.53E-06 | 1.77E-03 | 0.65 | N |
| 9 | PODXL | 1.58 | + | 8.15E-06 | 4.07E-03 | 5.20E-10 | 2.60E-07 | 9.66 | N |
| 10 | IL20RA | 1.49 | - | 1.01E-05 | 5.07E-03 | 2.73E-06 | 1.36E-03 | 1.31 | Y |
| 11 | CHAC1 | 2.17 | - | 1.10E-05 | 5.50E-03 | 4.98E-09 | 2.49E-06 | 7.70 | N |
| 12 | PPAP2B | 1.80 | + | 1.33E-05 | 6.64E-03 | 1.74E-09 | 8.68E-07 | 8.94 | N |
| 13 | GNA15 | 0.80 | - | 1.58E-05 | 7.90E-03 | 3.06E-03 | 1 | -5.26 | N |
| 14 | HMGA1 | 0.92 | - | 1.93E-05 | 9.65E-03 | 8.30E-04 | 4.15E-01 | -3.76 | N |
| 15 | FAM180B | 0.92 | - | 2.35E-05 | 1.17E-02 | 4.38E-05 | 2.19E-02 | -0.62 | N |
| 16 | CXCL2 | 1.34 | - | 3.34E-05 | 1.67E-02 | 6.51E-06 | 3.25E-03 | 1.64 | Y |
| 17 | HERC2P2 | 1.70 | - | 4.88E-05 | 2.44E-02 | 2.12E-05 | 1.06E-02 | 0.83 | N |
| 18 | PIP5KL1 | 1.51 | - | 5.64E-05 | 2.82E-02 | 1.69E-09 | 8.45E-07 | 10.42 | N |
| 19 | TRIB3 | 1.55 | - | 1.03E-04 | 5.15E-02 | 7.28E-07 | 3.64E-04 | 4.95 | Y |
| 20 | WDR62 | 1.99 | - | 1.18E-04 | 5.88E-02 | 3.22E-08 | 1.61E-05 | 8.20 | N |
| 21 | PLEKHO1 | 1.31 | - | 1.32E-04 | 6.58E-02 | 1.17E-06 | 5.86E-04 | 4.72 | N |
| 22 | TGFBR2 | 1.80 | + | 1.43E-04 | 7.16E-02 | 2.00E-05 | 9.98E-03 | 1.97 | N |
| 23 | SERPINA7 | 1.09 | + | 1.47E-04 | 7.33E-02 | 6.79E-05 | 3.39E-02 | 0.77 | N |
| 24 | MAGED4B | 1.90 | - | 2.32E-04 | 1.16E-01 | 2.71E-06 | 1.36E-03 | 4.45 | N |
| 25 | COL6A3 | 1.07 | - | 2.41E-04 | 1.20E-01 | 5.74E-04 | 2.87E-01 | -0.87 | N |
| 26 | CEACAM22P | 2.03 | - | 2.41E-04 | 1.20E-01 | 3.80E-08 | 1.90E-05 | 8.75 | N |
| 27 | GDA | 1.26 | + | 2.51E-04 | 1.25E-01 | 4.78E-04 | 2.39E-01 | -0.64 | N |
| 28 | SIX2 | 1.01 | - | 2.67E-04 | 1.33E-01 | 2.20E-04 | 1.10E-01 | 0.19 | N |
| 29 | LDHD | 2.04 | + | 2.86E-04 | 1.43E-01 | 4.26E-04 | 2.13E-01 | -0.40 | N |
| 30 | NME1 | 2.46 | - | 3.33E-04 | 1.66E-01 | 3.56E-07 | 1.78E-04 | 6.84 | N |
| 31 | LIMD2 | 1.39 | - | 4.15E-04 | 2.07E-01 | 1.34E-05 | 6.72E-03 | 3.43 | N |
| 32 | HMBOX1 | 2.27 | + | 4.85E-04 | 2.42E-01 | 2.47E-04 | 1.23E-01 | 0.68 | N |
| 33 | THRSP | 1.31 | - | 5.77E-04 | 2.89E-01 | 9.47E-05 | 4.73E-02 | 1.81 | N |
| 34 | HIST1H2BF | 1.05 | - | 7.22E-04 | 3.61E-01 | 1.50E-04 | 7.51E-02 | 1.57 | Y |
| 35 | KLHL9 | 3.55 | + | 7.41E-04 | 3.71E-01 | 1.14E-09 | 5.71E-07 | 13.38 | N |
| 36 | ENPP4 | 2.81 | + | 7.68E-04 | 3.84E-01 | 1.73E-05 | 8.65E-03 | 3.79 | N |
| 37 | RBM43 | 1.58 | + | 7.98E-04 | 3.99E-01 | 1.24E-05 | 6.19E-03 | 4.17 | N |
| 38 | PDZK1 | 1.59 | + | 8.04E-04 | 4.02E-01 | 1.10E-05 | 5.50E-03 | 4.29 | N |
| 39 | IMPDH1 | 2.06 | - | 9.48E-04 | 4.74E-01 | 1.79E-08 | 8.96E-06 | 10.88 | N |
| 40 | RAPGEF5 | 1.59 | + | 1.12E-03 | 5.58E-01 | 1.64E-06 | 8.19E-04 | 6.52 | N |
| 41 | MPP3 | 1.83 | - | 1.16E-03 | 5.78E-01 | 1.18E-05 | 5.89E-03 | 4.59 | N |
| 42 | GALR1 | 2.56 | + | 1.18E-03 | 5.90E-01 | 1.58E-04 | 7.92E-02 | 2.01 | N |
| 43 | SFRP2 | 1.30 | - | 1.34E-03 | 6.71E-01 | 7.79E-03 | 1 | -1.76 | N |
| 44 | DNAJB14 | 2.94 | + | 1.40E-03 | 6.98E-01 | 3.47E-06 | 1.73E-03 | 6.00 | N |
| 45 | GDF7 | 1.37 | + | 1.70E-03 | 8.50E-01 | 4.07E-04 | 2.04E-01 | 1.43 | N |
| 46 | BRWD3 | 2.30 | + | 1.71E-03 | 8.55E-01 | 5.79E-04 | 2.90E-01 | 1.08 | N |
| 47 | TRABD | 2.56 | - | 1.75E-03 | 8.76E-01 | 8.03E-09 | 4.02E-06 | 12.29 | N |
| 48 | CCDC135 | 1.87 | - | 1.97E-03 | 9.85E-01 | 5.94E-03 | 1 | -1.10 | N |
| 49 | DMP1 | 1.30 | - | 2.14E-03 | 1 | 3.55E-04 | 1.77E-01 | 1.80 | N |
| 50 | SLC8A1 | 1.20 | + | 2.49E-03 | 1 | 2.54E-02 | 1 | -2.32 | N |
| 51 | SIGLEC16 | 0.70 | - | 2.61E-03 | 1 | 3.28E-04 | 1.64E-01 | 2.07 | N |
| 52 | F8 | 1.05 | + | 2.63E-03 | 1 | 1.69E-06 | 8.47E-04 | 7.35 | Y |
| 53 | PBX1 | 1.72 | + | 2.81E-03 | 1 | 3.20E-04 | 1.60E-01 | 2.17 | Y |
| 54 | HGFAC | 1.14 | - | 3.32E-03 | 1 | 4.83E-05 | 2.42E-02 | 4.23 | N |
| 55 | KPNA5 | 2.25 | + | 3.61E-03 | 1 | 1.47E-02 | 1 | -1.40 | N |
| 56 | TMPRSS5 | 1.97 | - | 3.92E-03 | 1 | 1.32E-05 | 6.58E-03 | 5.70 | N |
| 57 | GEMIN8P4 | 1.95 | - | 4.53E-03 | 1 | 2.21E-07 | 1.11E-04 | 9.93 | N |
| 58 | RAD54B | 1.65 | - | 4.58E-03 | 1 | 1.13E-06 | 5.67E-04 | 8.30 | N |
| 59 | C6orf125 | 2.42 | - | 4.70E-03 | 1 | 2.17E-06 | 1.09E-03 | 7.68 | N |
| 60 | RUNDC3A | 1.68 | - | 4.76E-03 | 1 | 5.02E-05 | 2.51E-02 | 4.55 | N |
| 61 | GJB2 | 1.03 | - | 4.96E-03 | 1 | 1.03E-03 | 5.15E-01 | 1.57 | N |
| 62 | ERN1 | 1.52 | + | 4.98E-03 | 1 | 2.25E-02 | 1 | -1.51 | N |
| 63 | ZNF695 | 2.11 | - | 5.44E-03 | 1 | 3.08E-05 | 1.54E-02 | 5.17 | N |
| 64 | SLC22A20 | 1.32 | - | 5.55E-03 | 1 | 7.15E-04 | 3.57E-01 | 2.05 | N |
| 65 | STXBP1 | 1.46 | + | 5.57E-03 | 1 | 4.13E-03 | 1 | 0.30 | N |
| 66 | SLC25A46 | 2.77 | + | 6.12E-03 | 1 | 3.20E-04 | 1.60E-01 | 2.95 | N |
| 67 | CP | 1.50 | - | 6.21E-03 | 1 | 8.38E-03 | 1 | -0.30 | Y |
| 68 | MAPK8IP1 | 1.67 | + | 6.39E-03 | 1 | 2.88E-05 | 1.44E-02 | 5.40 | N |
| 69 | RUNX1T1 | 1.41 | + | 6.61E-03 | 1 | 7.36E-03 | 1 | -0.11 | Y |
| 70 | ID2 | 2.04 | + | 6.71E-03 | 1 | 2.48E-02 | 1 | -1.31 | N |
| 71 | PTPRN2 | 1.51 | + | 6.77E-03 | 1 | 1.13E-02 | 1 | -0.51 | N |
| 72 | FABP1 | 1.51 | + | 6.94E-03 | 1 | 1.36E-02 | 1 | -0.67 | N |
| 73 | SYT5 | 0.84 | - | 7.36E-03 | 1 | 3.86E-04 | 1.93E-01 | 2.95 | N |
| 74 | PATL2 | 1.52 | - | 7.37E-03 | 1 | 2.26E-02 | 1 | -1.12 | N |
| 75 | EMILIN2 | 1.32 | - | 7.45E-03 | 1 | 2.95E-02 | 1 | -1.37 | N |
| 76 | C12orf45 | 2.24 | - | 7.59E-03 | 1 | 2.62E-07 | 1.31E-04 | 10.27 | N |
| 77 | CHRM1 | 1.12 | + | 8.04E-03 | 1 | 1.09E-02 | 1 | -0.30 | N |
| 78 | LOC100133612 | 1.88 | - | 8.50E-03 | 1 | 3.42E-04 | 1.71E-01 | 3.21 | N |
| 79 | DHRS4L1 | 1.84 | + | 8.67E-03 | 1 | 3.27E-02 | 1 | -1.33 | N |
| 80 | LOC149134 | 1.63 | - | 9.70E-03 | 1 | 2.87E-05 | 1.43E-02 | 5.82 | N |
| 81 | RAB11FIP5 | 2.29 | + | 1.01E-02 | 1 | 3.41E-03 | 1 | 1.08 | N |
| 82 | RALGAPA2 | 2.67 | + | 1.03E-02 | 1 | 6.94E-03 | 1 | 0.39 | N |
| 83 | RNF111 | 3.83 | + | 1.05E-02 | 1 | 1.08E-03 | 5.41E-01 | 2.27 | N |
| 84 | FLJ14107 | 1.36 | - | 1.07E-02 | 1 | 3.79E-05 | 1.89E-02 | 5.64 | N |
| 85 | LRRC46 | 1.61 | - | 1.08E-02 | 1 | 5.71E-05 | 2.85E-02 | 5.24 | N |
| 86 | RIT1 | 1.75 | + | 1.09E-02 | 1 | 4.07E-05 | 2.04E-02 | 5.59 | N |
| 87 | CNKSR1 | 1.85 | - | 1.11E-02 | 1 | 2.18E-02 | 1 | -0.68 | N |
| 88 | EPC2 | 3.05 | + | 1.20E-02 | 1 | 5.22E-05 | 2.61E-02 | 5.43 | N |
| 89 | ZNF827 | 2.48 | + | 1.20E-02 | 1 | 1.75E-03 | 8.73E-01 | 1.93 | N |
| 90 | IRX6 | 1.58 | - | 1.21E-02 | 1 | 3.06E-05 | 1.53E-02 | 5.98 | N |
| 91 | EIF4E3 | 2.09 | + | 1.22E-02 | 1 | 4.26E-06 | 2.13E-03 | 7.96 | N |
| 92 | UCK2 | 2.29 | - | 1.22E-02 | 1 | 1.74E-07 | 8.71E-05 | 11.16 | N |
| 93 | LOC650623 | 1.96 | + | 1.22E-02 | 1 | 3.41E-01 | 1 | -3.33 | N |
| 94 | SRD5A2 | 1.54 | - | 1.23E-02 | 1 | 1.82E-03 | 9.08E-01 | 1.91 | N |
| 95 | C8orf77 | 2.30 | - | 1.23E-02 | 1 | 1.93E-04 | 9.67E-02 | 4.16 | N |
| 96 | RAD54L2 | 2.95 | + | 1.28E-02 | 1 | 1.12E-03 | 5.60E-01 | 2.44 | N |
| 97 | SOX13 | 1.71 | + | 1.29E-02 | 1 | 8.50E-04 | 4.25E-01 | 2.72 | N |
| 98 | PP14571 | 1.23 | - | 1.38E-02 | 1 | 3.81E-03 | 1 | 1.29 | N |
| 99 | HEY1 | 2.23 | + | 1.41E-02 | 1 | 1.26E-01 | 1 | -2.19 | N |
| 100 | ENKUR | 1.08 | - | 1.44E-02 | 1 | 1.76E-03 | 8.82E-01 | 2.10 | N |
| 101 | BAI2 | 2.03 | - | 1.48E-02 | 1 | 1.65E-04 | 8.25E-02 | 4.50 | N |
| 102 | F7 | 1.14 | - | 1.50E-02 | 1 | 1.53E-03 | 7.66E-01 | 2.28 | Y |
| 103 | CIB1 | 1.86 | - | 1.57E-02 | 1 | 6.08E-05 | 3.04E-02 | 5.56 | N |
| 104 | SPTY2D1 | 3.25 | + | 1.58E-02 | 1 | 5.28E-04 | 2.64E-01 | 3.40 | N |
| 105 | LRRC37A | 2.03 | - | 1.59E-02 | 1 | 2.58E-05 | 1.29E-02 | 6.42 | N |
| 106 | SLC16A11 | 1.48 | + | 1.62E-02 | 1 | 5.16E-03 | 1 | 1.14 | N |
| 107 | PALMD | 1.67 | + | 1.64E-02 | 1 | 2.96E-04 | 1.48E-01 | 4.01 | N |
| 108 | CXorf56 | 2.99 | + | 1.70E-02 | 1 | 1.61E-03 | 8.03E-01 | 2.36 | N |
| 109 | ZWINT | 2.56 | - | 1.74E-02 | 1 | 4.19E-03 | 1 | 1.43 | N |
| 110 | BCO2 | 2.60 | - | 1.79E-02 | 1 | 1.71E-06 | 8.56E-04 | 9.25 | N |
| 111 | CIDECP | 3.18 | - | 2.01E-02 | 1 | 5.81E-08 | 2.91E-05 | 12.75 | N |
| 112 | MAPK10 | 2.41 | + | 2.10E-02 | 1 | 2.05E-02 | 1 | 0.02 | Y |
| 113 | SLC25A21 | 1.09 | + | 2.12E-02 | 1 | 2.80E-02 | 1 | -0.28 | N |
| 114 | ARPC1B | 1.79 | - | 2.14E-02 | 1 | 3.83E-07 | 1.91E-04 | 10.93 | N |
| 115 | HFE | 2.34 | + | 2.19E-02 | 1 | 3.17E-02 | 1 | -0.37 | Y |
| 116 | EIF2C1 | 2.47 | + | 2.27E-02 | 1 | 1.05E-06 | 5.24E-04 | 9.98 | Y |
| 117 | GLT25D1 | 2.25 | - | 2.32E-02 | 1 | 9.76E-11 | 4.88E-08 | 19.29 | N |
| 118 | C16orf75 | 2.54 | - | 2.40E-02 | 1 | 7.26E-03 | 1 | 1.20 | N |
| 119 | NOS1AP | 1.47 | + | 2.41E-02 | 1 | 9.03E-04 | 4.52E-01 | 3.29 | N |
| 120 | CKM | 1.74 | - | 2.46E-02 | 1 | 1.25E-02 | 1 | 0.67 | N |
| 121 | C17orf89 | 1.65 | - | 2.46E-02 | 1 | 7.21E-07 | 3.61E-04 | 10.44 | N |
| 122 | ABAT | 1.75 | + | 2.47E-02 | 1 | 7.94E-03 | 1 | 1.13 | N |
| 123 | SRXN1 | 2.44 | - | 2.51E-02 | 1 | 8.52E-04 | 4.26E-01 | 3.38 | N |
| 124 | GALNT12 | 1.91 | + | 2.52E-02 | 1 | 1.19E-04 | 5.97E-02 | 5.35 | N |
| 125 | RNASE4 | 1.53 | + | 2.56E-02 | 1 | 1.20E-04 | 6.01E-02 | 5.36 | N |
| 126 | SNX27 | 2.86 | + | 2.61E-02 | 1 | 3.24E-01 | 1 | -2.52 | N |
| 127 | E2F3 | 3.29 | - | 2.74E-02 | 1 | 2.33E-09 | 1.16E-06 | 16.28 | N |
| 128 | LOC100270710 | 2.61 | - | 2.74E-02 | 1 | 5.07E-06 | 2.53E-03 | 8.59 | N |
| 129 | SERPINB8 | 1.56 | - | 2.75E-02 | 1 | 3.18E-02 | 1 | -0.14 | N |
| 130 | CD36 | 1.53 | + | 2.87E-02 | 1 | 3.07E-02 | 1 | -0.07 | Y |
| 131 | ITGA1 | 1.51 | + | 3.01E-02 | 1 | 7.49E-03 | 1 | 1.39 | Y |
| 132 | PPP4R2 | 2.94 | + | 3.02E-02 | 1 | 9.14E-04 | 4.57E-01 | 3.50 | N |
| 133 | ZNF184 | 1.96 | + | 3.10E-02 | 1 | 1.57E-03 | 7.86E-01 | 2.98 | N |
| 134 | FAHD2B | 2.71 | - | 3.36E-02 | 1 | 3.10E-02 | 1 | 0.08 | N |
| 135 | CD84 | 0.67 | + | 3.79E-02 | 1 | 1.06E-01 | 1 | -1.03 | Y |
| 136 | PLEKHG4 | 2.35 | - | 3.84E-02 | 1 | 1.77E-04 | 8.83E-02 | 5.38 | N |
| 137 | PTPN2 | 3.25 | - | 3.89E-02 | 1 | 6.08E-05 | 3.04E-02 | 6.46 | Y |
| 138 | FOXA3 | 1.34 | - | 3.91E-02 | 1 | 2.30E-01 | 1 | -1.77 | N |
| 139 | HAAO | 1.79 | + | 3.92E-02 | 1 | 1.42E-02 | 1 | 1.02 | N |
| 140 | C6orf27 | 2.43 | + | 3.99E-02 | 1 | 1.63E-03 | 8.16E-01 | 3.20 | N |
| 141 | COPG2 | 2.75 | - | 4.16E-02 | 1 | 1.80E-04 | 9.00E-02 | 5.44 | N |
| 142 | PIP5K1B | 2.09 | + | 4.18E-02 | 1 | 4.18E-02 | 1 | 0.00 | N |
| 143 | PLGLB2 | 1.95 | - | 4.19E-02 | 1 | 1.48E-03 | 7.40E-01 | 3.34 | N |
| 144 | KIAA1210 | 1.53 | + | 4.23E-02 | 1 | 7.17E-02 | 1 | -0.53 | N |
| 145 | FXYD6 | 2.28 | + | 4.28E-02 | 1 | 2.55E-02 | 1 | 0.52 | N |
| 146 | FAM114A2 | 3.30 | + | 4.29E-02 | 1 | 7.95E-04 | 3.98E-01 | 3.99 | N |
| 147 | RPL29 | 2.60 | - | 4.43E-02 | 1 | 4.21E-06 | 2.11E-03 | 9.26 | N |
| 148 | BBS7 | 2.49 | + | 4.50E-02 | 1 | 1.14E-04 | 5.68E-02 | 5.98 | N |
| 149 | YDJC | 2.23 | - | 4.50E-02 | 1 | 4.95E-05 | 2.47E-02 | 6.81 | N |
| 150 | SOX30 | 1.44 | - | 4.63E-02 | 1 | 1.43E-03 | 7.17E-01 | 3.47 | N |
| 151 | CTSW | 0.79 | - | 5.03E-02 | 1 | 4.04E-02 | 1 | 0.22 | Y |
| 152 | EMG1 | 2.88 | - | 5.09E-02 | 1 | 5.58E-05 | 2.79E-02 | 6.82 | N |
| 153 | ARL6 | 3.49 | + | 5.17E-02 | 1 | 3.55E-05 | 1.77E-02 | 7.29 | N |
| 154 | ATP1B1 | 2.59 | + | 5.19E-02 | 1 | 3.00E-02 | 1 | 0.55 | N |
| 155 | LCAT | 1.67 | - | 5.51E-02 | 1 | 3.66E-03 | 1 | 2.71 | N |
| 156 | ZNF114 | 1.35 | - | 5.53E-02 | 1 | 2.12E-02 | 1 | 0.96 | N |
| 157 | PREPL | 3.04 | + | 5.59E-02 | 1 | 6.93E-05 | 3.46E-02 | 6.69 | N |
| 158 | ZHX1 | 3.24 | + | 5.73E-02 | 1 | 2.52E-03 | 1 | 3.12 | N |
| 159 | FAM167B | 1.34 | + | 5.73E-02 | 1 | 6.34E-02 | 1 | -0.10 | N |
| 160 | DPYS | 1.44 | + | 5.77E-02 | 1 | 7.80E-03 | 1 | 2.00 | N |
| 161 | ZSCAN12 | 2.55 | + | 5.86E-02 | 1 | 1.94E-02 | 1 | 1.10 | N |
| 162 | ZDHHC18 | 2.40 | - | 5.96E-02 | 1 | 9.36E-08 | 4.68E-05 | 13.36 | N |
| 163 | ARRB1 | 1.67 | + | 6.05E-02 | 1 | 3.13E-03 | 1 | 2.96 | Y |
| 164 | SLC35B4 | 2.96 | + | 6.08E-02 | 1 | 1.58E-03 | 7.90E-01 | 3.65 | N |
| 165 | TPD52L2 | 3.11 | - | 6.13E-02 | 1 | 9.87E-11 | 4.93E-08 | 20.25 | N |
| 166 | MMADHC | 3.03 | + | 6.19E-02 | 1 | 3.95E-04 | 1.98E-01 | 5.05 | N |
| 167 | NAT8L | 1.44 | - | 6.50E-02 | 1 | 2.59E-02 | 1 | 0.92 | N |
| 168 | GADD45A | 2.61 | + | 6.69E-02 | 1 | 1.20E-02 | 1 | 1.71 | N |
| 169 | PF4V1 | 1.26 | - | 6.78E-02 | 1 | 9.08E-02 | 1 | -0.29 | Y |
| 170 | YOD1 | 2.53 | + | 7.19E-02 | 1 | 3.54E-01 | 1 | -1.59 | N |
| 171 | C8orf76 | 3.21 | - | 7.30E-02 | 1 | 1.33E-08 | 6.66E-06 | 15.52 | N |
| 172 | BTBD18 | 1.50 | + | 7.53E-02 | 1 | 5.83E-01 | 1 | -2.05 | N |
| 173 | USH1C | 1.76 | + | 7.55E-02 | 1 | 1.01E-02 | 1 | 2.02 | N |
| 174 | ZNF627 | 3.62 | + | 7.86E-02 | 1 | 1.99E-05 | 9.95E-03 | 8.28 | N |
| 175 | ZC3H3 | 2.43 | - | 7.93E-02 | 1 | 1.60E-07 | 8.01E-05 | 13.11 | N |
| 176 | TOX2 | 1.35 | + | 8.14E-02 | 1 | 3.70E-02 | 1 | 0.79 | N |
| 177 | SETD8 | 2.95 | - | 8.16E-02 | 1 | 3.57E-07 | 1.78E-04 | 12.34 | N |
| 178 | SPRED2 | 2.72 | + | 8.35E-02 | 1 | 1.11E-03 | 5.55E-01 | 4.32 | N |
| 179 | ACSM2B | 1.50 | + | 8.38E-02 | 1 | 3.66E-02 | 1 | 0.83 | N |
| 180 | TUBB8 | 1.62 | - | 8.40E-02 | 1 | 1.04E-02 | 1 | 2.09 | N |
| 181 | KLHL29 | 1.67 | - | 8.43E-02 | 1 | 3.26E-04 | 1.63E-01 | 5.56 | N |
| 182 | LUC7L3 | 1.95 | - | 8.65E-02 | 1 | 1.87E-05 | 9.36E-03 | 8.44 | N |
| 183 | RFPL2 | 1.23 | - | 8.72E-02 | 1 | 1.86E-02 | 1 | 1.54 | N |
| 184 | DNAH14 | 2.37 | - | 8.75E-02 | 1 | 3.33E-05 | 1.67E-02 | 7.87 | N |
| 185 | KIAA1737 | 3.06 | + | 8.79E-02 | 1 | 1.19E-05 | 5.94E-03 | 8.91 | N |
| 186 | CLN5 | 3.40 | + | 8.80E-02 | 1 | 1.79E-03 | 8.95E-01 | 3.90 | N |
| 187 | EHD3 | 1.63 | + | 8.92E-02 | 1 | 6.18E-03 | 1 | 2.67 | N |
| 188 | THOC6 | 2.45 | - | 9.10E-02 | 1 | 7.41E-05 | 3.71E-02 | 7.11 | N |
| 189 | LOC100289341 | 2.24 | + | 9.41E-02 | 1 | 1.70E-01 | 1 | -0.59 | N |
| 190 | ZNF860 | 1.81 | + | 1.01E-01 | 1 | 8.23E-03 | 1 | 2.51 | N |
| 191 | LOC729603 | 1.64 | - | 1.02E-01 | 1 | 1.85E-03 | 9.24E-01 | 4.01 | N |
| 192 | RPL30 | 2.38 | - | 1.04E-01 | 1 | 1.05E-03 | 5.24E-01 | 4.60 | N |
| 193 | UBA7 | 2.26 | - | 1.05E-01 | 1 | 1.41E-04 | 7.03E-02 | 6.62 | Y |
| 194 | TTL | 3.52 | - | 1.07E-01 | 1 | 1.84E-04 | 9.21E-02 | 6.36 | N |
| 195 | GPR4 | 1.75 | + | 1.08E-01 | 1 | 2.20E-01 | 1 | -0.71 | N |
| 196 | LOC100192379 | 0.76 | - | 1.09E-01 | 1 | 2.84E-01 | 1 | -0.96 | N |
| 197 | ANXA2P1 | 1.62 | - | 1.11E-01 | 1 | 6.32E-02 | 1 | 0.56 | N |
| 198 | CROCCL2 | 1.46 | - | 1.12E-01 | 1 | 8.43E-03 | 1 | 2.59 | N |
| 199 | CDKN2AIP | 3.35 | + | 1.13E-01 | 1 | 1.64E-04 | 8.18E-02 | 6.54 | N |
| 200 | C6orf154 | 1.45 | - | 1.15E-01 | 1 | 4.17E-03 | 1 | 3.32 | N |
| 201 | B4GALT7 | 2.11 | - | 1.15E-01 | 1 | 1.51E-04 | 7.54E-02 | 6.64 | N |
| 202 | C16orf68 | 3.43 | - | 1.17E-01 | 1 | 2.04E-05 | 1.02E-02 | 8.65 | N |
| 203 | LOC285359 | 2.22 | - | 1.19E-01 | 1 | 4.94E-05 | 2.47E-02 | 7.79 | N |
| 204 | FGFR1OP | 1.59 | + | 1.20E-01 | 1 | 3.25E-01 | 1 | -1.00 | N |
| 205 | PCSK1N | 1.24 | + | 1.24E-01 | 1 | 2.98E-01 | 1 | -0.88 | N |
| 206 | LGI3 | 1.18 | - | 1.26E-01 | 1 | 3.86E-02 | 1 | 1.18 | N |
| 207 | ARID3A | 2.55 | - | 1.27E-01 | 1 | 2.75E-02 | 1 | 1.53 | N |
| 208 | EDARADD | 2.00 | + | 1.28E-01 | 1 | 1.85E-01 | 1 | -0.37 | N |
| 209 | TTC21B | 1.85 | + | 1.30E-01 | 1 | 4.23E-02 | 1 | 1.12 | N |
| 210 | GDPD4 | 1.39 | + | 1.30E-01 | 1 | 4.65E-01 | 1 | -1.27 | N |
| 211 | PLAC8L1 | 1.81 | - | 1.31E-01 | 1 | 2.33E-02 | 1 | 1.73 | N |
| 212 | FXYD7 | 1.51 | - | 1.33E-01 | 1 | 3.81E-02 | 1 | 1.25 | N |
| 213 | FAM190B | 3.33 | + | 1.33E-01 | 1 | 3.05E-03 | 1 | 3.78 | N |
| 214 | NTRK2 | 1.31 | + | 1.34E-01 | 1 | 9.95E-02 | 1 | 0.30 | N |
| 215 | UGT1A7 | 1.19 | - | 1.34E-01 | 1 | 4.52E-02 | 1 | 1.09 | N |
| 216 | TSPAN5 | 1.90 | + | 1.36E-01 | 1 | 5.07E-02 | 1 | 0.99 | N |
| 217 | MAP3K5 | 3.08 | + | 1.36E-01 | 1 | 8.65E-02 | 1 | 0.45 | Y |
| 218 | C11orf2 | 2.63 | + | 1.36E-01 | 1 | 8.93E-02 | 1 | 0.42 | N |
| 219 | ANKRD19 | 1.10 | - | 1.37E-01 | 1 | 4.93E-02 | 1 | 1.02 | N |
| 220 | PYCRL | 2.25 | - | 1.38E-01 | 1 | 1.04E-05 | 5.21E-03 | 9.49 | N |
| 221 | ESYT1 | 2.74 | + | 1.41E-01 | 1 | 8.18E-03 | 1 | 2.85 | N |
| 222 | TRAK2 | 3.61 | + | 1.44E-01 | 1 | 4.30E-05 | 2.15E-02 | 8.12 | N |
| 223 | VANGL2 | 2.13 | - | 1.45E-01 | 1 | 1.45E-02 | 1 | 2.30 | N |
| 224 | PHKB | 4.43 | + | 1.47E-01 | 1 | 1.89E-04 | 9.47E-02 | 6.65 | N |
| 225 | MKX | 1.29 | - | 1.47E-01 | 1 | 4.01E-02 | 1 | 1.30 | N |
| 226 | TEX10 | 3.02 | + | 1.47E-01 | 1 | 1.35E-02 | 1 | 2.39 | N |
| 227 | LOC100128788 | 1.08 | - | 1.48E-01 | 1 | 2.33E-05 | 1.17E-02 | 8.75 | N |
| 228 | KCTD14 | 1.57 | + | 1.50E-01 | 1 | 2.28E-02 | 1 | 1.88 | N |
| 229 | SMPD4 | 3.20 | - | 1.51E-01 | 1 | 5.69E-08 | 2.84E-05 | 14.79 | N |
| 230 | TNFRSF11B | 1.24 | + | 1.51E-01 | 1 | 1.46E-01 | 1 | 0.03 | Y |
| 231 | PCA3 | 1.32 | + | 1.51E-01 | 1 | 4.71E-01 | 1 | -1.14 | N |
| 232 | ARSK | 2.34 | + | 1.53E-01 | 1 | 1.56E-02 | 1 | 2.28 | N |
| 233 | TCEANC | 2.58 | + | 1.57E-01 | 1 | 1.51E-01 | 1 | 0.03 | N |
| 234 | CHST10 | 2.18 | - | 1.57E-01 | 1 | 1.04E-02 | 1 | 2.71 | N |
| 235 | HS3ST3B1 | 1.76 | + | 1.57E-01 | 1 | 6.86E-01 | 1 | -1.47 | N |
| 236 | GGT8P | 1.43 | + | 1.59E-01 | 1 | 6.67E-02 | 1 | 0.87 | N |
| 237 | DISP1 | 2.17 | + | 1.60E-01 | 1 | 4.77E-04 | 2.39E-01 | 5.81 | N |
| 238 | SF3B4 | 2.73 | - | 1.61E-01 | 1 | 2.22E-07 | 1.11E-04 | 13.49 | N |
| 239 | TMEM59L | 1.24 | - | 1.61E-01 | 1 | 2.42E-02 | 1 | 1.90 | N |
| 240 | N4BP2L1 | 1.85 | + | 1.63E-01 | 1 | 3.24E-01 | 1 | -0.69 | N |
| 241 | GADD45G | 1.71 | - | 1.63E-01 | 1 | 9.14E-02 | 1 | 0.58 | N |
| 242 | DSP | 2.71 | + | 1.75E-01 | 1 | 4.82E-02 | 1 | 1.29 | N |
| 243 | ARPC5 | 2.02 | - | 1.76E-01 | 1 | 5.69E-05 | 2.85E-02 | 8.04 | Y |
| 244 | BCL2L13 | 4.56 | + | 1.78E-01 | 1 | 1.40E-02 | 1 | 2.55 | N |
| 245 | CCNY | 2.70 | + | 1.79E-01 | 1 | 5.82E-02 | 1 | 1.12 | N |
| 246 | IP6K3 | 1.64 | + | 1.81E-01 | 1 | 4.83E-01 | 1 | -0.98 | N |
| 247 | CFLP1 | 2.11 | - | 1.82E-01 | 1 | 3.91E-02 | 1 | 1.54 | N |
| 248 | SRGAP3 | 2.40 | - | 1.83E-01 | 1 | 2.07E-01 | 1 | -0.12 | N |
| 249 | C1orf187 | 1.63 | - | 1.83E-01 | 1 | 3.79E-02 | 1 | 1.58 | N |
| 250 | VCAM1 | 1.26 | + | 1.84E-01 | 1 | 1.46E-01 | 1 | 0.23 | Y |
| 251 | BAZ1B | 2.95 | + | 1.84E-01 | 1 | 4.08E-01 | 1 | -0.80 | N |
| 252 | RBMS3 | 1.64 | + | 1.91E-01 | 1 | 3.41E-02 | 1 | 1.72 | N |
| 253 | RBM34 | 2.66 | - | 1.98E-01 | 1 | 4.38E-05 | 2.19E-02 | 8.42 | N |
| 254 | C3orf1 | 3.73 | - | 1.98E-01 | 1 | 9.90E-03 | 1 | 3.00 | N |
| 255 | SRPRB | 2.74 | - | 2.05E-01 | 1 | 2.85E-02 | 1 | 1.97 | N |
| 256 | SELO | 2.65 | - | 2.15E-01 | 1 | 3.38E-03 | 1 | 4.15 | N |
| 257 | CHADL | 1.99 | + | 2.16E-01 | 1 | 5.53E-01 | 1 | -0.94 | N |
| 258 | ART4 | 1.29 | + | 2.19E-01 | 1 | 3.74E-01 | 1 | -0.54 | Y |
| 259 | ARL3 | 3.12 | + | 2.19E-01 | 1 | 1.88E-02 | 1 | 2.46 | N |
| 260 | FADS1 | 2.48 | - | 2.21E-01 | 1 | 7.94E-03 | 1 | 3.33 | N |
| 261 | ZNF837 | 2.68 | - | 2.22E-01 | 1 | 9.46E-03 | 1 | 3.16 | N |
| 262 | SPCS1 | 3.33 | - | 2.22E-01 | 1 | 1.54E-01 | 1 | 0.37 | N |
| 263 | SLC41A2 | 1.48 | + | 2.23E-01 | 1 | 4.03E-01 | 1 | -0.59 | N |
| 264 | WWP2 | 3.29 | + | 2.23E-01 | 1 | 5.12E-02 | 1 | 1.47 | N |
| 265 | DND1 | 2.49 | - | 2.30E-01 | 1 | 1.16E-03 | 5.79E-01 | 5.29 | N |
| 266 | KDM6B | 1.74 | + | 2.31E-01 | 1 | 2.32E-02 | 1 | 2.30 | N |
| 267 | KIAA1267 | 4.00 | + | 2.33E-01 | 1 | 1.03E-01 | 1 | 0.82 | N |
| 268 | OR7D2 | 1.39 | - | 2.35E-01 | 1 | 4.90E-02 | 1 | 1.57 | N |
| 269 | LPIN3 | 2.13 | - | 2.36E-01 | 1 | 6.49E-04 | 3.25E-01 | 5.90 | N |
| 270 | TBCE | 2.97 | - | 2.38E-01 | 1 | 1.21E-04 | 6.07E-02 | 7.58 | N |
| 271 | S1PR5 | 2.09 | - | 2.44E-01 | 1 | 6.04E-01 | 1 | -0.91 | N |
| 272 | MOCS3 | 3.65 | + | 2.47E-01 | 1 | 6.67E-02 | 1 | 1.31 | N |
| 273 | CCNC | 3.62 | + | 2.48E-01 | 1 | 3.30E-01 | 1 | -0.28 | N |
| 274 | PARP6 | 2.40 | - | 2.50E-01 | 1 | 1.22E-06 | 6.10E-04 | 12.23 | N |
| 275 | NT5C1B | 1.68 | - | 2.52E-01 | 1 | 1.96E-02 | 1 | 2.55 | N |
| 276 | POMT1 | 3.24 | + | 2.61E-01 | 1 | 2.63E-01 | 1 | -0.01 | N |
| 277 | TESK1 | 2.75 | - | 2.61E-01 | 1 | 1.52E-03 | 7.61E-01 | 5.15 | N |
| 278 | TCTEX1D2 | 2.52 | - | 2.64E-01 | 1 | 6.58E-02 | 1 | 1.39 | N |
| 279 | TAF4B | 2.01 | + | 2.64E-01 | 1 | 1.13E-01 | 1 | 0.85 | N |
| 280 | ZNF137 | 2.24 | - | 2.68E-01 | 1 | 4.49E-03 | 1 | 4.09 | N |
| 281 | SIL1 | 1.66 | - | 2.69E-01 | 1 | 1.17E-01 | 1 | 0.84 | N |
| 282 | LOC144486 | 1.90 | + | 2.74E-01 | 1 | 1.51E-02 | 1 | 2.90 | N |
| 283 | SLC25A45 | 1.82 | - | 2.75E-01 | 1 | 5.92E-02 | 1 | 1.54 | N |
| 284 | LOC152217 | 3.09 | - | 2.76E-01 | 1 | 1.61E-04 | 8.06E-02 | 7.44 | N |
| 285 | SGTB | 3.20 | + | 2.76E-01 | 1 | 7.60E-02 | 1 | 1.29 | N |
| 286 | GSTA4 | 2.10 | + | 2.82E-01 | 1 | 1.07E-01 | 1 | 0.97 | N |
| 287 | RPS27 | 1.62 | + | 2.84E-01 | 1 | 3.45E-01 | 1 | -0.19 | N |
| 288 | DKC1 | 2.94 | - | 2.88E-01 | 1 | 3.11E-02 | 1 | 2.22 | N |
| 289 | SLC38A6 | 1.96 | - | 2.89E-01 | 1 | 1.77E-02 | 1 | 2.79 | N |
| 290 | JAZF1 | 2.84 | + | 2.90E-01 | 1 | 4.46E-01 | 1 | -0.43 | N |
| 291 | KIF22 | 2.42 | - | 2.97E-01 | 1 | 6.54E-06 | 3.27E-03 | 10.72 | N |
| 292 | CCDC115 | 3.39 | + | 3.00E-01 | 1 | 4.45E-02 | 1 | 1.91 | N |
| 293 | ASL | 2.15 | + | 3.02E-01 | 1 | 4.02E-01 | 1 | -0.29 | N |
| 294 | ZNF594 | 2.11 | + | 3.04E-01 | 1 | 6.83E-01 | 1 | -0.81 | N |
| 295 | DUSP15 | 1.32 | - | 3.07E-01 | 1 | 5.40E-01 | 1 | -0.56 | N |
| 296 | RAB8B | 1.55 | + | 3.09E-01 | 1 | 1.10E-01 | 1 | 1.03 | N |
| 297 | SLC2A12 | 1.85 | - | 3.11E-01 | 1 | 7.66E-03 | 1 | 3.70 | N |
| 298 | NCKAP1L | 0.37 | - | 3.12E-01 | 1 | 6.05E-01 | 1 | -0.66 | Y |
| 299 | ENAH | 2.18 | - | 3.14E-01 | 1 | 7.60E-02 | 1 | 1.42 | Y |
| 300 | STK33 | 2.35 | - | 3.16E-01 | 1 | 1.56E-01 | 1 | 0.71 | N |
| 301 | FAM182B | 1.59 | - | 3.21E-01 | 1 | 1.14E-03 | 5.68E-01 | 5.64 | N |
| 302 | BTBD1 | 3.65 | + | 3.21E-01 | 1 | 5.73E-01 | 1 | -0.58 | N |
| 303 | RPUSD4 | 3.32 | + | 3.29E-01 | 1 | 1.45E-01 | 1 | 0.82 | N |
| 304 | FAM19A5 | 1.59 | + | 3.32E-01 | 1 | 6.33E-01 | 1 | -0.65 | N |
| 305 | SEC14L4 | 2.23 | - | 3.38E-01 | 1 | 5.77E-02 | 1 | 1.77 | N |
| 306 | TMX1 | 2.10 | + | 3.39E-01 | 1 | 8.22E-03 | 1 | 3.72 | Y |
| 307 | CRTAM | 0.57 | - | 3.42E-01 | 1 | 4.49E-01 | 1 | -0.27 | Y |
| 308 | TMEM91 | 1.53 | - | 3.44E-01 | 1 | 1.04E-01 | 1 | 1.20 | N |
| 309 | TRIM22 | 1.25 | + | 3.45E-01 | 1 | 8.75E-01 | 1 | -0.93 | Y |
| 310 | TNFRSF10D | 1.79 | + | 3.47E-01 | 1 | 1.79E-01 | 1 | 0.66 | Y |
| 311 | PHF16 | 3.14 | + | 3.49E-01 | 1 | 9.66E-03 | 1 | 3.59 | N |
| 312 | TMEM14C | 3.21 | - | 3.50E-01 | 1 | 1.49E-02 | 1 | 3.16 | N |
| 313 | ELP2 | 3.01 | + | 3.52E-01 | 1 | 1.50E-01 | 1 | 0.85 | N |
| 314 | CNOT6 | 2.67 | + | 3.57E-01 | 1 | 1.85E-02 | 1 | 2.96 | N |
| 315 | ZNF556 | 2.01 | + | 3.58E-01 | 1 | 6.35E-01 | 1 | -0.57 | N |
| 316 | ZNF451 | 3.91 | + | 3.60E-01 | 1 | 7.42E-01 | 1 | -0.72 | N |
| 317 | TSSK3 | 1.54 | - | 3.62E-01 | 1 | 9.03E-03 | 1 | 3.69 | N |
| 318 | PPA1 | 2.69 | - | 3.75E-01 | 1 | 6.62E-02 | 1 | 1.73 | N |
| 319 | ORAI2 | 2.02 | - | 3.76E-01 | 1 | 1.55E-04 | 7.75E-02 | 7.79 | N |
| 320 | MMP10 | 1.32 | - | 3.76E-01 | 1 | 1.21E-01 | 1 | 1.13 | N |
| 321 | SYNGR1 | 2.25 | + | 3.77E-01 | 1 | 1.84E-01 | 1 | 0.72 | N |
| 322 | COX11 | 2.75 | + | 3.78E-01 | 1 | 3.74E-02 | 1 | 2.31 | N |
| 323 | RFX8 | 1.28 | - | 3.85E-01 | 1 | 2.40E-01 | 1 | 0.47 | N |
| 324 | CHST15 | 1.46 | - | 3.86E-01 | 1 | 4.04E-02 | 1 | 2.26 | N |
| 325 | CD8B | 0.71 | - | 3.88E-01 | 1 | 5.30E-01 | 1 | -0.31 | Y |
| 326 | IL32 | 1.85 | - | 3.96E-01 | 1 | 1.66E-01 | 1 | 0.87 | Y |
| 327 | MPHOSPH10 | 3.29 | - | 3.98E-01 | 1 | 8.69E-05 | 4.35E-02 | 8.43 | N |
| 328 | CXCL16 | 1.32 | + | 4.05E-01 | 1 | 6.45E-02 | 1 | 1.84 | Y |
| 329 | GLRX3 | 3.27 | - | 4.06E-01 | 1 | 5.67E-02 | 1 | 1.97 | N |
| 330 | C2orf72 | 1.62 | + | 4.11E-01 | 1 | 1.02E-01 | 1 | 1.40 | N |
| 331 | WDR74 | 2.16 | - | 4.16E-01 | 1 | 1.07E-02 | 1 | 3.66 | N |
| 332 | ALMS1 | 2.83 | + | 4.17E-01 | 1 | 1.50E-02 | 1 | 3.33 | N |
| 333 | HMG20A | 4.93 | + | 4.18E-01 | 1 | 3.47E-01 | 1 | 0.19 | N |
| 334 | GAR1 | 3.49 | - | 4.19E-01 | 1 | 7.26E-02 | 1 | 1.75 | N |
| 335 | KLF8 | 1.49 | + | 4.23E-01 | 1 | 5.79E-01 | 1 | -0.31 | N |
| 336 | RPS12 | 2.69 | + | 4.29E-01 | 1 | 1.42E-02 | 1 | 3.41 | N |
| 337 | RFPL4B | 0.96 | + | 4.30E-01 | 1 | 7.85E-01 | 1 | -0.60 | N |
| 338 | C9orf23 | 2.80 | + | 4.34E-01 | 1 | 6.60E-01 | 1 | -0.42 | N |
| 339 | HOXC6 | 2.03 | - | 4.35E-01 | 1 | 2.27E-02 | 1 | 2.95 | N |
| 340 | PFKL | 2.44 | + | 4.44E-01 | 1 | 8.37E-01 | 1 | -0.63 | N |
| 341 | SUPT16H | 3.45 | + | 4.45E-01 | 1 | 1.46E-01 | 1 | 1.11 | N |
| 342 | SLC27A1 | 2.74 | + | 4.46E-01 | 1 | 9.46E-01 | 1 | -0.75 | N |
| 343 | KCNJ2 | 1.24 | - | 4.48E-01 | 1 | 5.89E-01 | 1 | -0.27 | N |
| 344 | EFNA1 | 1.70 | + | 4.50E-01 | 1 | 9.17E-01 | 1 | -0.71 | N |
| 345 | POLR2J2 | 1.79 | - | 4.52E-01 | 1 | 1.70E-01 | 1 | 0.98 | N |
| 346 | POLH | 2.33 | + | 4.56E-01 | 1 | 9.66E-01 | 1 | -0.75 | N |
| 347 | CLK3 | 2.96 | - | 4.63E-01 | 1 | 4.63E-03 | 1 | 4.61 | N |
| 348 | SPATA17 | 2.09 | + | 4.69E-01 | 1 | 5.12E-01 | 1 | -0.09 | N |
| 349 | GOSR2 | 4.06 | + | 4.70E-01 | 1 | 6.26E-01 | 1 | -0.29 | N |
| 350 | IP6K1 | 3.88 | + | 4.71E-01 | 1 | 3.75E-02 | 1 | 2.53 | N |
| 351 | PSMC2 | 3.53 | - | 4.83E-01 | 1 | 2.31E-02 | 1 | 3.04 | Y |
| 352 | MRPS33 | 3.08 | - | 4.88E-01 | 1 | 4.42E-01 | 1 | 0.10 | N |
| 353 | TMEM161A | 2.97 | - | 4.88E-01 | 1 | 1.20E-02 | 1 | 3.71 | N |
| 354 | HMG20B | 2.75 | - | 4.98E-01 | 1 | 1.48E-03 | 7.40E-01 | 5.82 | N |
| 355 | C14orf169 | 2.07 | - | 4.99E-01 | 1 | 2.28E-01 | 1 | 0.78 | N |
| 356 | LETM1 | 3.74 | = | 4.99E-01 | 1 | 9.19E-04 | 4.59E-01 | 6.30 | N |
| 357 | IL11RA | 2.47 | + | 5.04E-01 | 1 | 2.48E-01 | 1 | 0.71 | Y |
| 358 | ATP5G2 | 2.56 | - | 5.14E-01 | 1 | 6.58E-03 | 1 | 4.36 | N |
| 359 | ADAMTSL1 | 2.11 | + | 5.15E-01 | 1 | 2.33E-01 | 1 | 0.79 | N |
| 360 | ANKRD34A | 1.95 | - | 5.17E-01 | 1 | 1.73E-03 | 8.64E-01 | 5.70 | N |
| 361 | TSKS | 1.54 | - | 5.19E-01 | 1 | 1.43E-01 | 1 | 1.29 | N |
| 362 | STIP1 | 3.73 | + | 5.22E-01 | 1 | 4.43E-02 | 1 | 2.47 | N |
| 363 | ASXL1 | 3.54 | + | 5.23E-01 | 1 | 1.35E-03 | 6.77E-01 | 5.96 | N |
| 364 | WASH2P | 2.71 | - | 5.24E-01 | 1 | 9.84E-03 | 1 | 3.97 | N |
| 365 | MRM1 | 2.77 | = | 5.26E-01 | 1 | 1.76E-03 | 8.78E-01 | 5.70 | N |
| 366 | PRMT7 | 3.18 | + | 5.30E-01 | 1 | 4.20E-02 | 1 | 2.54 | N |
| 367 | LONRF1 | 3.38 | + | 5.35E-01 | 1 | 8.60E-01 | 1 | -0.47 | N |
| 368 | TET1 | 2.21 | + | 5.38E-01 | 1 | 3.81E-01 | 1 | 0.34 | N |
| 369 | SKP2 | 3.34 | + | 5.41E-01 | 1 | 8.30E-01 | 1 | -0.43 | N |
| 370 | ALDH3A1 | 1.38 | - | 5.43E-01 | 1 | 1.53E-01 | 1 | 1.27 | N |
| 371 | WNT9A | 0.89 | + | 5.49E-01 | 1 | 3.64E-01 | 1 | 0.41 | N |
| 372 | SDHC | 2.89 | + | 5.50E-01 | 1 | 3.98E-02 | 1 | 2.63 | N |
| 373 | TATDN2 | 4.24 | + | 5.52E-01 | 1 | 6.97E-01 | 1 | -0.23 | N |
| 374 | LOC730101 | 1.28 | - | 5.52E-01 | 1 | 4.48E-01 | 1 | 0.21 | N |
| 375 | C16orf71 | 1.58 | - | 5.54E-01 | 1 | 3.30E-01 | 1 | 0.52 | N |
| 376 | ZNF7 | 3.22 | + | 5.55E-01 | 1 | 1.87E-05 | 9.34E-03 | 10.30 | N |
| 377 | ZMYND10 | 1.44 | - | 5.57E-01 | 1 | 1.04E-01 | 1 | 1.68 | N |
| 378 | SLC39A6 | 1.73 | + | 5.57E-01 | 1 | 9.61E-01 | 1 | -0.54 | N |
| 379 | DGKD | 1.89 | + | 5.58E-01 | 1 | 1.66E-01 | 1 | 1.21 | Y |
| 380 | CIB2 | 2.38 | + | 5.61E-01 | 1 | 5.90E-01 | 1 | -0.05 | N |
| 381 | RAB3C | 2.14 | - | 5.62E-01 | 1 | 9.18E-01 | 1 | -0.49 | N |
| 382 | MRAS | 1.84 | + | 5.66E-01 | 1 | 3.16E-01 | 1 | 0.58 | N |
| 383 | EEF1D | 1.96 | - | 5.69E-01 | 1 | 1.39E-03 | 6.97E-01 | 6.01 | N |
| 384 | C22orf27 | 2.11 | - | 5.70E-01 | 1 | 4.29E-02 | 1 | 2.59 | N |
| 385 | PLCD1 | 3.10 | + | 5.73E-01 | 1 | 1.10E-01 | 1 | 1.65 | N |
| 386 | HLA-DRB5 | 0.79 | - | 5.80E-01 | 1 | 5.15E-01 | 1 | 0.12 | Y |
| 387 | SNAP47 | 3.22 | + | 5.85E-01 | 1 | 8.43E-02 | 1 | 1.94 | N |
| 388 | PELO | 3.15 | + | 5.86E-01 | 1 | 6.32E-02 | 1 | 2.23 | N |
| 389 | OCEL1 | 2.04 | - | 5.91E-01 | 1 | 3.58E-02 | 1 | 2.80 | N |
| 390 | PARL | 2.90 | - | 5.92E-01 | 1 | 4.13E-02 | 1 | 2.66 | N |
| 391 | C1orf198 | 2.23 | + | 5.95E-01 | 1 | 2.08E-01 | 1 | 1.05 | N |
| 392 | RPS2P32 | 1.72 | + | 6.02E-01 | 1 | 6.75E-01 | 1 | -0.11 | N |
| 393 | RARG | 2.10 | + | 6.05E-01 | 1 | 2.28E-01 | 1 | 0.98 | N |
| 394 | EPDR1 | 2.75 | - | 6.05E-01 | 1 | 9.05E-01 | 1 | -0.40 | N |
| 395 | OSTC | 2.31 | - | 6.06E-01 | 1 | 1.93E-01 | 1 | 1.15 | N |
| 396 | RAB3A | 2.74 | + | 6.08E-01 | 1 | 5.10E-01 | 1 | 0.18 | N |
| 397 | MXD1 | 2.32 | + | 6.12E-01 | 1 | 5.03E-02 | 1 | 2.50 | Y |
| 398 | ZNF524 | 1.62 | - | 6.13E-01 | 1 | 6.32E-02 | 1 | 2.27 | N |
| 399 | BCKDK | 2.31 | - | 6.14E-01 | 1 | 1.28E-02 | 1 | 3.87 | N |
| 400 | TMEM86A | 2.06 | - | 6.17E-01 | 1 | 4.47E-01 | 1 | 0.32 | N |
| 401 | SLC6A8 | 1.50 | - | 6.17E-01 | 1 | 1.69E-01 | 1 | 1.29 | N |
| 402 | HIST2H2BE | 2.03 | - | 6.19E-01 | 1 | 5.11E-02 | 1 | 2.49 | N |
| 403 | PRSS16 | 1.80 | + | 6.20E-01 | 1 | 8.83E-01 | 1 | -0.35 | Y |
| 404 | STK19 | 1.57 | - | 6.22E-01 | 1 | 1.12E-01 | 1 | 1.72 | N |
| 405 | IKZF4 | 3.23 | + | 6.30E-01 | 1 | 3.98E-01 | 1 | 0.46 | Y |
| 406 | HCG18 | 3.80 | + | 6.31E-01 | 1 | 1.33E-01 | 1 | 1.56 | N |
| 407 | ATP8A2 | 1.88 | + | 6.33E-01 | 1 | 4.21E-01 | 1 | 0.41 | N |
| 408 | PRUNE | 2.62 | + | 6.35E-01 | 1 | 6.14E-01 | 1 | 0.03 | N |
| 409 | FIGNL2 | 1.71 | - | 6.35E-01 | 1 | 2.63E-01 | 1 | 0.88 | N |
| 410 | TNFRSF4 | 1.51 | - | 6.44E-01 | 1 | 4.30E-01 | 1 | 0.40 | Y |
| 411 | ZNF530 | 3.21 | + | 6.48E-01 | 1 | 3.21E-01 | 1 | 0.70 | N |
| 412 | CCL14-CCL15 | 1.36 | - | 6.48E-01 | 1 | 6.26E-01 | 1 | 0.04 | N |
| 413 | GCET2 | 1.41 | + | 6.49E-01 | 1 | 8.78E-01 | 1 | -0.30 | Y |
| 414 | SLFN13 | 1.59 | + | 6.52E-01 | 1 | 7.01E-01 | 1 | -0.07 | N |
| 415 | UBOX5 | 4.55 | + | 6.54E-01 | 1 | 1.13E-01 | 1 | 1.76 | N |
| 416 | C2orf29 | 2.32 | = | 6.57E-01 | 1 | 8.63E-03 | 1 | 4.33 | N |
| 417 | KCTD15 | 1.91 | + | 6.58E-01 | 1 | 1.98E-01 | 1 | 1.20 | N |
| 418 | SEC11A | 3.99 | + | 6.59E-01 | 1 | 3.59E-01 | 1 | 0.61 | N |
| 419 | SEL1L | 2.73 | + | 6.60E-01 | 1 | 1.58E-01 | 1 | 1.43 | N |
| 420 | SCMH1 | 2.76 | + | 6.61E-01 | 1 | 9.03E-01 | 1 | -0.31 | N |
| 421 | BAX | 2.73 | - | 6.63E-01 | 1 | 1.08E-01 | 1 | 1.81 | Y |
| 422 | ATP5D | 1.35 | - | 6.66E-01 | 1 | 1.34E-02 | 1 | 3.91 | N |
| 423 | MTPAP | 4.68 | + | 6.86E-01 | 1 | 8.15E-01 | 1 | -0.17 | N |
| 424 | DKFZP586I1420 | 2.55 | + | 6.87E-01 | 1 | 7.18E-01 | 1 | -0.04 | N |
| 425 | GALNT5 | 1.23 | + | 6.95E-01 | 1 | 9.09E-01 | 1 | -0.27 | N |
| 426 | ACP1 | 3.60 | + | 6.98E-01 | 1 | 1.90E-01 | 1 | 1.30 | Y |
| 427 | C3orf54 | 1.57 | + | 6.99E-01 | 1 | 9.21E-01 | 1 | -0.28 | N |
| 428 | MRPL11 | 3.12 | + | 7.04E-01 | 1 | 1.16E-01 | 1 | 1.80 | N |
| 429 | GPR17 | 1.27 | + | 7.04E-01 | 1 | 6.74E-01 | 1 | 0.04 | N |
| 430 | SLC10A4 | 0.89 | = | 7.09E-01 | 1 | 6.35E-01 | 1 | 0.11 | N |
| 431 | PIR | 1.87 | + | 7.12E-01 | 1 | 4.49E-02 | 1 | 2.76 | N |
| 432 | WDR66 | 1.22 | + | 7.15E-01 | 1 | 8.86E-01 | 1 | -0.22 | N |
| 433 | RAB28 | 4.23 | + | 7.17E-01 | 1 | 3.87E-01 | 1 | 0.62 | N |
| 434 | LOC100268168 | 1.56 | + | 7.18E-01 | 1 | 2.37E-01 | 1 | 1.11 | N |
| 435 | RAB1B | 3.91 | + | 7.20E-01 | 1 | 7.64E-01 | 1 | -0.06 | N |
| 436 | HNRNPUL1 | 4.17 | + | 7.20E-01 | 1 | 8.67E-01 | 1 | -0.19 | N |
| 437 | TES | 2.81 | + | 7.21E-01 | 1 | 3.83E-01 | 1 | 0.63 | N |
| 438 | ADHFE1 | 2.34 | + | 7.29E-01 | 1 | 8.55E-01 | 1 | -0.16 | N |
| 439 | LOC643008 | 1.48 | - | 7.31E-01 | 1 | 2.91E-01 | 1 | 0.92 | N |
| 440 | HOXA5 | 2.43 | + | 7.37E-01 | 1 | 3.16E-01 | 1 | 0.85 | N |
| 441 | BCL2L1 | 3.07 | + | 7.53E-01 | 1 | 5.37E-01 | 1 | 0.34 | Y |
| 442 | GNPDA1 | 2.55 | + | 7.62E-01 | 1 | 1.44E-01 | 1 | 1.67 | N |
| 443 | TRAPPC4 | 3.68 | + | 7.65E-01 | 1 | 7.88E-01 | 1 | -0.03 | N |
| 444 | MMP2 | 1.91 | + | 7.68E-01 | 1 | 1.14E-01 | 1 | 1.91 | N |
| 445 | SRF | 1.94 | + | 7.85E-01 | 1 | 4.77E-01 | 1 | 0.50 | N |
| 446 | DUSP1 | 1.33 | + | 7.85E-01 | 1 | 8.22E-01 | 1 | -0.05 | N |
| 447 | KIAA0467 | 2.41 | + | 7.87E-01 | 1 | 2.01E-01 | 1 | 1.37 | N |
| 448 | CHSY3 | 1.46 | + | 7.91E-01 | 1 | 8.02E-02 | 1 | 2.29 | N |
| 449 | CASP8AP2 | 2.54 | + | 7.95E-01 | 1 | 4.40E-01 | 1 | 0.59 | Y |
| 450 | FRG2C | 0.72 | + | 7.96E-01 | 1 | 9.94E-01 | 1 | -0.22 | N |
| 451 | MEIS1 | 2.35 | + | 7.98E-01 | 1 | 4.43E-01 | 1 | 0.59 | N |
| 452 | ME2 | 2.56 | + | 7.99E-01 | 1 | 9.32E-01 | 1 | -0.15 | N |
| 453 | SDC1 | 2.00 | + | 8.00E-01 | 1 | 9.42E-01 | 1 | -0.16 | N |
| 454 | HDAC3 | 3.29 | + | 8.09E-01 | 1 | 9.17E-01 | 1 | -0.13 | N |
| 455 | CEP63 | 3.96 | + | 8.11E-01 | 1 | 3.67E-01 | 1 | 0.79 | N |
| 456 | CCDC112 | 1.71 | + | 8.16E-01 | 1 | 3.12E-01 | 1 | 0.96 | N |
| 457 | CLDN16 | 1.69 | + | 8.18E-01 | 1 | 4.09E-01 | 1 | 0.69 | N |
| 458 | AQP3 | 1.25 | + | 8.24E-01 | 1 | 4.77E-02 | 1 | 2.85 | N |
| 459 | ALS2CR12 | 1.70 | + | 8.27E-01 | 1 | 6.96E-01 | 1 | 0.17 | N |
| 460 | NLRX1 | 3.50 | + | 8.32E-01 | 1 | 8.48E-01 | 1 | -0.02 | Y |
| 461 | GNPTAB | 2.63 | + | 8.33E-01 | 1 | 6.98E-01 | 1 | 0.18 | N |
| 462 | EGFL7 | 1.50 | + | 8.38E-01 | 1 | 5.43E-01 | 1 | 0.43 | N |
| 463 | LEFTY2 | 1.43 | - | 8.39E-01 | 1 | 8.31E-01 | 1 | 0.01 | N |
| 464 | CNTROB | 2.94 | + | 8.40E-01 | 1 | 1.68E-02 | 1 | 3.91 | N |
| 465 | PPP1R15A | 2.02 | + | 8.49E-01 | 1 | 5.33E-01 | 1 | 0.47 | N |
| 466 | C20orf196 | 3.83 | + | 8.55E-01 | 1 | 8.76E-01 | 1 | -0.02 | N |
| 467 | CA12 | 3.17 | - | 8.65E-01 | 1 | 1.43E-01 | 1 | 1.80 | N |
| 468 | CHIC2 | 2.31 | + | 8.73E-01 | 1 | 5.96E-01 | 1 | 0.38 | N |
| 469 | KCTD4 | 1.34 | - | 8.76E-01 | 1 | 8.65E-02 | 1 | 2.32 | N |
| 470 | MRPL19 | 3.68 | + | 8.84E-01 | 1 | 9.70E-01 | 1 | -0.09 | N |
| 471 | HIST1H2BK | 1.74 | + | 8.89E-01 | 1 | 3.87E-01 | 1 | 0.83 | N |
| 472 | RIOK1 | 3.33 | + | 8.89E-01 | 1 | 1.64E-01 | 1 | 1.69 | N |
| 473 | ZNF766 | 4.05 | + | 8.90E-01 | 1 | 7.20E-01 | 1 | 0.21 | N |
| 474 | MCM3 | 3.76 | + | 8.92E-01 | 1 | 9.80E-02 | 1 | 2.21 | N |
| 475 | CD1D | 1.51 | + | 8.94E-01 | 1 | 8.17E-01 | 1 | 0.09 | Y |
| 476 | SERHL | 3.05 | + | 8.96E-01 | 1 | 6.03E-01 | 1 | 0.40 | N |
| 477 | DCP1B | 3.28 | + | 8.98E-01 | 1 | 7.47E-02 | 1 | 2.49 | N |
| 478 | CCT7 | 3.95 | + | 8.98E-01 | 1 | 9.97E-01 | 1 | -0.10 | N |
| 479 | INTS4L1 | 2.11 | + | 9.03E-01 | 1 | 2.56E-01 | 1 | 1.26 | N |
| 480 | LOC647288 | 1.57 | - | 9.04E-01 | 1 | 1.53E-01 | 1 | 1.78 | N |
| 481 | LOC441455 | 1.82 | + | 9.05E-01 | 1 | 3.61E-01 | 1 | 0.92 | N |
| 482 | C1orf126 | 2.02 | + | 9.10E-01 | 1 | 3.33E-01 | 1 | 1.01 | N |
| 483 | SFRS1 | 4.28 | + | 9.12E-01 | 1 | 2.03E-02 | 1 | 3.81 | N |
| 484 | SNAP23 | 3.43 | + | 9.13E-01 | 1 | 1.08E-01 | 1 | 2.13 | N |
| 485 | FLJ46111 | 1.63 | + | 9.14E-01 | 1 | 1.26E-01 | 1 | 1.98 | N |
| 486 | RNPC3 | 1.69 | + | 9.16E-01 | 1 | 1.54E-01 | 1 | 1.78 | N |
| 487 | IPO9 | 3.31 | + | 9.19E-01 | 1 | 5.68E-01 | 1 | 0.48 | N |
| 488 | TCF15 | 2.24 | + | 9.19E-01 | 1 | 3.61E-01 | 1 | 0.93 | N |
| 489 | FLJ35220 | 2.63 | + | 9.21E-01 | 1 | 2.16E-01 | 1 | 1.45 | N |
| 490 | DGCR14 | 3.01 | + | 9.26E-01 | 1 | 1.25E-01 | 1 | 2.00 | N |
| 491 | LOC653113 | 1.90 | + | 9.36E-01 | 1 | 6.90E-01 | 1 | 0.31 | N |
| 492 | AKAP8 | 2.20 | + | 9.38E-01 | 1 | 3.13E-02 | 1 | 3.40 | N |
| 493 | ZNF815 | 2.56 | + | 9.40E-01 | 1 | 8.66E-01 | 1 | 0.08 | N |
| 494 | ALG11 | 3.65 | + | 9.41E-01 | 1 | 4.28E-01 | 1 | 0.79 | N |
| 495 | HOXB4 | 3.23 | + | 9.45E-01 | 1 | 4.21E-02 | 1 | 3.11 | N |
| 496 | EXOSC3 | 3.20 | + | 9.69E-01 | 1 | 7.32E-01 | 1 | 0.28 | N |
| 497 | LPGAT1 | 1.58 | + | 9.70E-01 | 1 | 2.77E-01 | 1 | 1.25 | N |
| 498 | SYPL2 | 1.53 | + | 9.79E-01 | 1 | 2.79E-01 | 1 | 1.25 | N |
| 499 | MYL6B | 2.07 | + | 9.82E-01 | 1 | 3.66E-01 | 1 | 0.99 | N |
| 500 | LARS | 3.50 | + | 9.96E-01 | 1 | 0.3649 | 1 | 1.00 | N |

*Values in the column “Survival direction”: "-" = more survivors in the low-expression third. "+" = more survivors in the higher-expression third.

**Gray background – genes that are associated with the Immune system.

**Supplementary table 6: Involvement of gender, age, and pathalogical stage for significant genes in the lowest CVR genes group**

| **#** | **Gene name** | **Third type** | **Average expression (RSEM)** | **Pathological stages*** | | | | **differences between stages 1&2 and 3&4 (chi-squared p.value)** | **Gender*** | |  |  |  |
| --- | --- | --- | --- | --- | --- | --- | --- | --- | --- | --- | --- | --- | --- |
|  |  |  |  | **1** | **2** | **3** | **4** |  | **Males** | **Females** | **Gender differences (chi-squared p.value)** | **Average age (days)** | **Age differences (t-test p.value)** |
|  |  | Lower expression | 28.7895 | 70 | 15 | 45 | 42 |  | 110 | 62 |  | -22308.28 |  |
| 1 | HLA-DRA | Higher expression | 49.7288 | 91 | 24 | 39 | 18 | 0.0015 | 118 | 54 | 0.4247 | -22603.48 | 0.5235 |
|  |  | Lower expression | 0.0086 | 104 | 25 | 33 | 10 |  | 107 | 65 |  | -22117.40 |  |
| 2 | GPR84 | Higher expression | 0.0445 | 62 | 16 | 51 | 43 | 0 | 106 | 66 | 1 | -22332.00 | 0.6505 |
|  |  | Lower expression | 0.1047 | 112 | 18 | 26 | 16 |  | 108 | 64 |  | -21841.21 |  |
| 3 | FCGR1B | Higher expression | 0.3267 | 55 | 17 | 51 | 49 | 0 | 118 | 54 | 0.3067 | -22784.35 | 0.0543 |
|  |  | Lower expression | 0.0339 | 111 | 17 | 28 | 16 |  | 112 | 60 |  | -22099.06 |  |
| 4 | FCGR1C | Higher expression | 0.1185 | 52 | 17 | 60 | 43 | 0 | 116 | 56 | 0.7322 | -22545.02 | 0.357 |
|  |  | Lower expression | 0.5372 | 93 | 26 | 39 | 14 |  | 108 | 64 |  | -22250.70 |  |
| 5 | AIF1 | Higher expression | 1.0607 | 71 | 17 | 41 | 43 | 0.001 | 115 | 57 | 0.4981 | -22430.87 | 0.7131 |
|  |  | Lower expression | 0.7054 | 92 | 21 | 36 | 23 |  | 114 | 58 |  | -22587.35 |  |
| 6 | IL10RA | Higher expression | 1.4402 | 66 | 21 | 47 | 38 | 0.0063 | 100 | 72 | 0.1483 | -22412.82 | 0.7141 |
|  |  | Lower expression | 0.0449 | 100 | 18 | 38 | 16 |  | 106 | 66 |  | -22511.35 |  |
| 7 | SP140 | Higher expression | 0.1381 | 52 | 23 | 53 | 44 | 0 | 120 | 52 | 0.1398 | -22242.05 | 0.5776 |
|  |  | Lower expression | 0.5053 | 97 | 25 | 33 | 17 |  | 107 | 65 |  | -22272.48 |  |
| 8 | LAIR1 | Higher expression | 1.0775 | 66 | 15 | 55 | 36 | 0 | 113 | 59 | 0.5745 | -22730.22 | 0.3559 |
|  |  | Lower expression | 0.1131 | 115 | 17 | 27 | 13 |  | 105 | 67 |  | -21872.79 |  |
| 9 | FCGR1A | Higher expression | 0.3562 | 51 | 21 | 55 | 45 | 0 | 117 | 55 | 0.2151 | -22758.92 | 0.0651 |
|  |  | Lower expression | 0.2677 | 100 | 22 | 32 | 18 |  | 100 | 72 |  | -22713.67 |  |
| 10 | SLAMF8 | Higher expression | 0.8402 | 71 | 17 | 41 | 43 | 0.0003 | 122 | 50 | 0.0179 | -22345.83 | 0.4315 |
|  |  | Lower expression | 0.0290 | 103 | 23 | 31 | 15 |  | 114 | 58 |  | -22180.43 |  |
| 11 | BATF | Higher expression | 0.1320 | 48 | 21 | 57 | 46 | 0 | 118 | 54 | 0.73 | -22591.46 | 0.3955 |
|  |  | Lower expression | 0.0022 | 111 | 22 | 30 | 9 |  | 98 | 74 |  | -22354.53 |  |
| 12 | HAMP | Higher expression | 0.0531 | 59 | 18 | 52 | 43 | 0 | 115 | 57 | 0.0756 | -22329.20 | 0.9566 |
|  |  | Lower expression | 0.0658 | 109 | 15 | 32 | 16 |  | 110 | 62 |  | -22178.98 |  |
| 13 | CD72 | Higher expression | 0.2228 | 57 | 18 | 49 | 48 | 0 | 117 | 55 | 0.4947 | -22628.50 | 0.3644 |
|  |  | Lower expression | 0.2391 | 92 | 21 | 40 | 19 |  | 111 | 61 |  | -22277.34 |  |
| 14 | PARVG | Higher expression | 0.7273 | 63 | 19 | 53 | 37 | 0.0011 | 110 | 62 | 1 | -22246.05 | 0.9487 |
|  |  | Lower expression | 0.1638 | 92 | 22 | 37 | 21 |  | 117 | 55 |  | -22782.41 |  |
| 15 | TRPM2 | Higher expression | 0.4586 | 74 | 17 | 44 | 37 | 0.0156 | 112 | 60 | 0.6476 | -21957.85 | 0.0871 |
|  |  | Lower expression | 0.1176 | 102 | 22 | 35 | 13 |  | 117 | 55 |  | -21906.18 |  |
| 16 | HCST | Higher expression | 0.4021 | 62 | 19 | 51 | 40 | 0 | 113 | 59 | 0.7311 | -22578.69 | 0.1642 |
|  |  | Lower expression | 0.0010 | 67 | 19 | 48 | 38 |  | 110 | 62 |  | -22834.52 |  |
| 17 | XCR1 | Higher expression | 0.0128 | 98 | 18 | 38 | 18 | 0.0015 | 119 | 53 | 0.3605 | -21817.63 | 0.039 |
|  |  | Lower expression | 2.1059 | 98 | 18 | 35 | 21 |  | 107 | 65 |  | -21986.09 |  |
| 18 | FCGR3A | Higher expression | 4.9755 | 72 | 21 | 44 | 35 | 0.0151 | 121 | 51 | 0.1382 | -23066.36 | 0.0174 |
|  |  | Lower expression | 0.1867 | 101 | 20 | 32 | 19 |  | 120 | 52 |  | -22190.30 |  |
| 19 | LILRB1 | Higher expression | 0.4457 | 66 | 20 | 53 | 33 | 0.0002 | 99 | 73 | 0.025 | -22861.24 | 0.1626 |
|  |  | Lower expression | 0.0017 | 92 | 19 | 38 | 23 |  | 121 | 51 |  | -22121.92 |  |
| 20 | ZNF80 | Higher expression | 0.0174 | 60 | 20 | 56 | 36 | 0.0011 | 100 | 72 | 0.0245 | -22656.85 | 0.2399 |
|  |  | Lower expression | 0.4887 | 92 | 21 | 39 | 20 |  | 111 | 61 |  | -22365.37 |  |
| 21 | RAC2 | Higher expression | 1.3657 | 58 | 16 | 53 | 45 | 0 | 114 | 58 | 0.8207 | -22539.44 | 0.7174 |
|  |  | Lower expression | 0.2129 | 94 | 23 | 39 | 16 |  | 110 | 62 |  | -22673.93 |  |
| 22 | WAS | Higher expression | 0.4981 | 60 | 17 | 52 | 43 | 0 | 107 | 65 | 0.8232 | -22385.16 | 0.5474 |
|  |  | Lower expression | 0.3261 | 89 | 25 | 33 | 25 |  | 122 | 50 |  | -22174.85 |  |
| 23 | CYTH4 | Higher expression | 0.7650 | 69 | 15 | 49 | 39 | 0.0016 | 99 | 73 | 0.0133 | -22705.67 | 0.2735 |
|  |  | Lower expression | 0.4199 | 95 | 25 | 37 | 15 |  | 111 | 61 |  | -22032.78 |  |
| 24 | SPI1 | Higher expression | 1.0048 | 67 | 16 | 54 | 35 | 0.0001 | 105 | 67 | 0.577 | -22322.35 | 0.5536 |
|  |  | Lower expression | 0.0579 | 102 | 24 | 31 | 15 |  | 98 | 74 |  | -22480.54 |  |
| 25 | TNFSF13B | Higher expression | 0.1708 | 54 | 18 | 56 | 44 | 0 | 127 | 45 | 0.0015 | -22775.53 | 0.5334 |
|  |  | Lower expression | 0.4558 | 92 | 23 | 34 | 23 |  | 121 | 51 |  | -22204.98 |  |
| 26 | MYO1F | Higher expression | 1.0383 | 69 | 19 | 49 | 35 | 0.0044 | 105 | 67 | 0.0885 | -22203.16 | 0.997 |
|  |  | Lower expression | 0.0870 | 81 | 28 | 40 | 23 |  | 113 | 59 |  | -22685.88 |  |
| 27 | GNA15 | Higher expression | 0.2404 | 75 | 14 | 45 | 38 | 0.0382 | 107 | 65 | 0.5745 | -22087.41 | 0.2141 |
|  |  | Lower expression | 0.0066 | 75 | 17 | 47 | 33 |  | 105 | 67 |  | -22602.59 |  |
| 28 | LILRA4 | Higher expression | 0.0552 | 90 | 25 | 38 | 19 | 0.0154 | 126 | 46 | 0.0217 | -22367.81 | 0.6233 |
|  |  | Lower expression | 0.0084 | 95 | 20 | 42 | 15 |  | 113 | 59 |  | -21825.77 |  |
| 29 | CTLA4 | Higher expression | 0.0641 | 62 | 19 | 47 | 44 | 0.0003 | 112 | 60 | 1 | -22108.48 | 0.546 |
|  |  | Lower expression | 0.5881 | 100 | 22 | 35 | 15 |  | 115 | 57 |  | -22243.53 |  |
| 30 | CCL5 | Higher expression | 2.8872 | 55 | 21 | 50 | 46 | 0 | 115 | 57 | 1 | -22506.96 | 0.5918 |
|  |  | Lower expression | 0.0017 | 104 | 17 | 31 | 20 |  | 116 | 56 |  | -21761.68 |  |
| 31 | IFNG | Higher expression | 0.0312 | 54 | 22 | 49 | 47 | 0 | 108 | 64 | 0.4284 | -22833.90 | 0.0198 |
|  |  | Lower expression | 0.0090 | 114 | 14 | 33 | 11 |  | 115 | 57 |  | -22049.44 |  |
| 32 | CXCL13 | Higher expression | 0.3126 | 54 | 18 | 55 | 45 | 0 | 116 | 56 | 1 | -22259.48 | 0.6564 |
|  |  | Lower expression | 0.4076 | 87 | 25 | 37 | 23 |  | 112 | 60 |  | -22565.39 |  |
| 33 | FERMT3 | Higher expression | 0.8446 | 72 | 14 | 53 | 33 | 0.0064 | 109 | 63 | 0.822 | -22223.37 | 0.4734 |
|  |  | Lower expression | 2.8231 | 89 | 25 | 38 | 20 |  | 112 | 60 |  | -22314.17 |  |
| 34 | C1QA | Higher expression | 6.9728 | 67 | 17 | 48 | 40 | 0.0016 | 116 | 56 | 0.7322 | -22506.29 | 0.6871 |
|  |  | Lower expression | 0.3646 | 105 | 20 | 31 | 16 |  | 124 | 48 |  | -22099.53 |  |
| 35 | IL2RG | Higher expression | 1.1681 | 62 | 15 | 53 | 42 | 0 | 109 | 63 | 0.1064 | -22412.97 | 0.5083 |
|  |  | Lower expression | 0.0093 | 92 | 16 | 43 | 21 |  | 112 | 60 |  | -22484.30 |  |
| 36 | CD80 | Higher expression | 0.0343 | 68 | 21 | 40 | 43 | 0.0498 | 111 | 61 | 1 | -22201.78 | 0.5454 |
|  |  | Lower expression | 3.0231 | 95 | 21 | 34 | 22 |  | 103 | 69 |  | -22615.12 |  |
| 37 | C1QB | Higher expression | 7.1365 | 68 | 19 | 46 | 39 | 0.0021 | 112 | 60 | 0.373 | -22509.38 | 0.8274 |
|  |  | Lower expression | 0.1141 | 106 | 19 | 30 | 17 |  | 112 | 60 |  | -22013.24 |  |
| 38 | PTPN7 | Higher expression | 0.4118 | 52 | 21 | 53 | 46 | 0 | 114 | 58 | 0.9096 | -22348.44 | 0.4888 |
|  |  | Lower expression | 0.5700 | 101 | 25 | 34 | 12 |  | 105 | 67 |  | -22333.56 |  |
| 39 | FCER1G | Higher expression | 1.2510 | 63 | 17 | 47 | 45 | 0 | 122 | 50 | 0.0686 | -21908.63 | 0.3736 |
|  |  | Lower expression | 0.1356 | 99 | 21 | 34 | 18 |  | 116 | 56 |  | -22610.49 |  |
| 40 | ARHGAP9 | Higher expression | 0.4368 | 68 | 17 | 51 | 36 | 0.0002 | 96 | 76 | 0.0352 | -22149.56 | 0.3313 |
|  |  | Lower expression | 0.1848 | 86 | 20 | 43 | 23 |  | 118 | 54 |  | -22436.17 |  |
| 41 | LILRB2 | Higher expression | 0.4279 | 68 | 21 | 48 | 35 | 0.0817 | 105 | 67 | 0.1754 | -22120.23 | 0.5153 |
|  |  | Lower expression | 0.0527 | 97 | 22 | 30 | 23 |  | 116 | 56 |  | -21972.11 |  |
| 42 | DERL3 | Higher expression | 0.4951 | 60 | 19 | 59 | 34 | 0 | 104 | 68 | 0.2167 | -22749.11 | 0.112 |
|  |  | Lower expression | 0.0651 | 98 | 19 | 36 | 19 |  | 118 | 54 |  | -22262.62 |  |
| 43 | LILRB3 | Higher expression | 0.2131 | 60 | 20 | 53 | 39 | 0.0001 | 106 | 66 | 0.2134 | -22419.62 | 0.7446 |
|  |  | Lower expression | 0.0007 | 100 | 22 | 37 | 13 |  | 114 | 58 |  | -21960.20 |  |
| 44 | JSRP1 | Higher expression | 0.0692 | 60 | 18 | 53 | 41 | 0 | 108 | 64 | 0.5731 | -22733.03 | 0.1147 |

*The counts represent the distribution of patients who were diagnosed with the mentioned pathological stages \ gender.
